# Census of the longhorn beetles (Coleoptera, Cerambycidae and Vesperidae) of the Macau SAR, China

**DOI:** 10.3897/zookeys.1049.65558

**Published:** 2021-07-22

**Authors:** Mei-Ying Lin, Renzo Perissinotto, Lynette Clennell

**Affiliations:** 1 Key Laboratory of Zoological Systematics and Evolution, Institute of Zoology, Chinese Academy of Sciences, 1–5 Beichen West Road, Chaoyang Dist., Beijing, 100101, China Institute of Zoology, Chinese Academy of Sciences Beijing China; 2 Institute for Coastal & Marine Research (CMR), Nelson Mandela University, P.O. Box 77000, Gqeberha 6031, South Africa Nelson Mandela University Gqeberha South Africa; 3 Macau Anglican College, 109-117 Avenida Padre Tomas Pereira, Taipa, Macau SAR, China Macau Anglican College Macau China

**Keywords:** Biodiversity, Cerambycidae, China, new records, Palaearctic Region, Vesperidae

## Abstract

An intensive census, extended over a period of approximately three and a half years, October 2017– May 2021, was conducted in the remaining green areas of the Macau SAR in order to provide an updated status of the biodiversity of longhorn beetles in this region. This insect group includes more than 36,000 species worldwide, subdivided into four families of mainly xylophagous or saproxylic insects, the Vesperidae, Oxypeltidae, Disteniidae, and Cerambycidae. They are of key importance in agricultural and forestry science, and are often used as an indicator of forest habitat health. A total of 52 species was recorded during this census, 2.6 times more than previously reported in the literature for this area. However, recorded abundances and frequency of occurrence for the various species were remarkably low, and of the 20 species previously reported for the region, some prominent ones remained unaccounted for. Among others, these include *Batocera horsfieldii* (Hope, 1839), *Apriona rugicollis* Chevrolat, 1852 [previously incorrectly reported as *Apriona germarii* (Hope, 1831)], *Aristobia reticulator* (Fabricius, 1781) [previously reported as *Aristobia testudo* (Voet, 1778)] and *Imantocera penicillata* (Hope, 1831). It is hypothesised that this may be related to the ongoing manipulation of the natural vegetation of the Macau SAR, which is rapidly being converted to plantations, city parks, and gardens. In particular, dead or dying trees and lower tree branches are systematically removed in order to improve the aesthetic appearance of these green areas. However, this process is also depriving xylophagous and saproxylic species of their essential habitats.

## Introduction

Longhorn beetles represent one of the largest groupings of extant insects with more than 36,000 species currently described worldwide (Leschen and Beutel 2014; Monné et al. 2017). The four families currently recognized within this grouping (Vesperidae, Oxypeltidae, Disteniidae, Cerambycidae) are part of the superfamily Chrysomeloidea. They have been often regarded as sufficiently distinct to possibly form a separate superfamily of Cerambycoidea, but this is not supported by results of mitochondrial genomic analysis (Nie et al. 2021). The predominantly xylophagous and saproxylic habits of their larval stages make them one of the most important groups of insects in the forestry and agricultural sciences. While crepuscular and nocturnal adult longhorn beetles are generally dull and sombre-coloured in their body aspect, diurnal species are mostly ornamented to brightly coloured species that use either Batesian mimicry or aposematism to protect themselves against potential predators (Švácha and Lawrence 2014). Also, with the exception of some Lamiinae, nocturnal species generally do not feed at the adult stage, while diurnal ones often seek high energy nutrition from either flowers, leaves, bark, fermenting fruits, or sap flows. Longhorn beetles are, therefore, important as pollinators but above all as recyclers of dead wood, and their diversity and abundance are used as indicators of forest habitat health (Švácha and Lawrence 2014).

Records of longhorn beetles from the Macau Special Administrative Region (SAR) of China are historically very scarce and, consequently, this territory normally does not feature in either the regional or global revisions of this insect group (e.g., Löbl and Smetana 2010; Danilevsky 2020). This sharply contrasts with the nearby Hong Kong SAR, where several comprehensive and dedicated publications have been produced to date on this insect group (e.g., Yiu 2009; Yiu and Yip 2011). To our knowledge, so far only two species have been described using Macau types and six published accounts have reported information on the species diversity of longhorn beetles in Macau. These include the earliest Gressitt’s (1951) monograph, the early 1990’s series by Easton (1991, 1992, 1993), the later general manual by Pun and Batalha (1997) and the recent catalogue by Lin and Yang (2019). Collectively, two species with type locality from Macau were included in Gressitt (1951), i.e., “*Chlorophorus macaumensis* (Chevrolat) and *Pterolophia annulata* (Chevrolat)”, 10 species were then reported in the three works of Easton (1991–1993), namely: “*Anoplophora chinensis* (Forster), *Batocera rubus* (L.), *Imantocera penicillata* (Hope), *Olenecamptus bilobus*, *Aeolesthes induta* (Newman), *Aristobia approximator* (Thomson), *Pyrestes haematica* Pascoe, *Chelidonium sinense* (Hope), *Chlorophorus annularis* (Fabricius) and *Xystrocera globosa* (Olivier)”. Pun and Batalha (1997), on the other hand, listed a total of 13 species, adding six new species on top of those already reported by Gressitt (1951) and Easton (1991–1993), namely: “*Apriona germari* (Hope), *Batocera horsfieldi* (Hope), *Glenea cantor* (Fabricius), *Megopis marginalis* (Fairmaire), *Oberea ferruginea* Thunberg and *Pothyne rugifrons* Gressitt”. Two more species were finally added in the catalogue by Lin and Yang (2019), i.e., “Pterolophia (Pterolophia) crassipes (Wiedemann) and *Purpuricenus temminckii sinensis* White”. Thus, the current total diversity formally reported in the literature for this group from Macau is 20 species.

The Macau SAR has a special local government structure within the “One Country – Two Systems” dispensation of 1999. It is a very prosperous region with per-capita incomes among the highest in the world. It is, however, also one of the most densely populated places on the planet and, consequently, under enormous residential and developmental pressure (Leong et al. 2017). Despite the massive urban development that the SAR has experienced over the last few decades, some pockets of natural vegetation still occur throughout its territory, albeit in a very fragmented manner and often encroached upon by alien species. These are mainly focused around 18 areas, where remnants of subtropical forest are currently administered as city parks and gardens, or in the largest cases as country parks. The ecological conditions of these areas are currently being assessed and biodiversity records are an essential component of this process, particularly in the field of terrestrial invertebrates for which there are still insufficient data available (cf. Direcção dos Serviços de Protecção Ambiental 2020). The main objective of this study is, therefore, to provide an updated account of the longhorn beetles of the Macau SAR, based on extended and frequent field surveys, comprehensive observation gathering methods and updated identification approaches using local and global expertise. Only three other similar studies have recently been completed for this region, on the ants (Hymenoptera, Formicidae) (Leong et al. 2017), the butterflies (Lepidoptera, Rhopalocera) (Department of Green Areas and Gardens, Municipal Affairs Bureau of Macao Special Administrative Region, Guangdong Institute of Applied Biological Resources 2019) and the fruit and flower chafers (Scarabaeidae, Cetoniinae) (Perissinotto and Clennell 2021), respectively. These will hopefully stimulate further research initiatives in the region and provide the local authorities with supporting information towards their ongoing environmental management and biodiversity conservation programmes. A recent survey undertaken by the authorities has shown that the overwhelming majority of the Macau population (i.e., 79% of questionnaire returns) regards as a priority the maintenance of the ecological integrity and biodiversity of its green areas (Direcção dos Serviços de Protecção Ambiental 2020).

## Materials and methods

The Macau SAR of China is biogeographically part of the Palaearctic Region, but is characterised by a subtropical climate and is close to the interface with the Oriental Region. Thus, many species that occur within its boundaries are actually also found further south and are shared with the latter region. Although the area has undergone extreme urban transformation during the last few decades, some pockets of its natural terrestrial vegetation still remain. Their plant assemblages include five vegetation types, namely coniferous forest, coniferous and broad-leaved mixed forest, evergreen broad-leaved forest, evergreen and deciduous broad-leaved mixed forest, and shrub (Peng et al. 2014; Direcção dos Serviços de Protecção Ambiental 2020).

**Figure 1.**
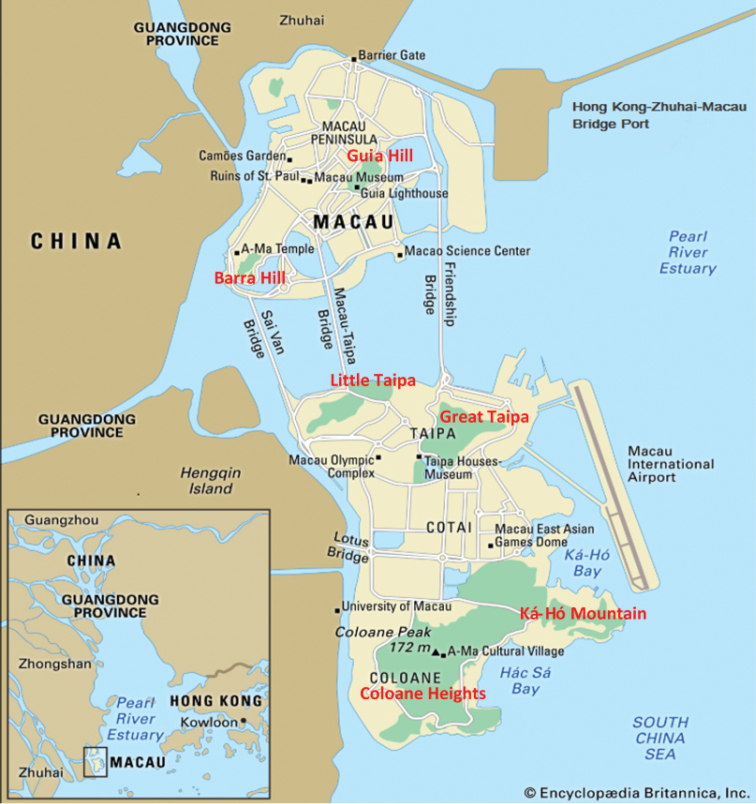
Map of the Macau SAR showing its various components including the Peninsula, the islands of Taipa and Coloane, as well as the reclaimed lands of Cotai, the International Airport, and the Hong Kong – Zhuhai – Macau Bridge Port (adapted from https://www.britannica.com; used with permission).

**Figure 2.**
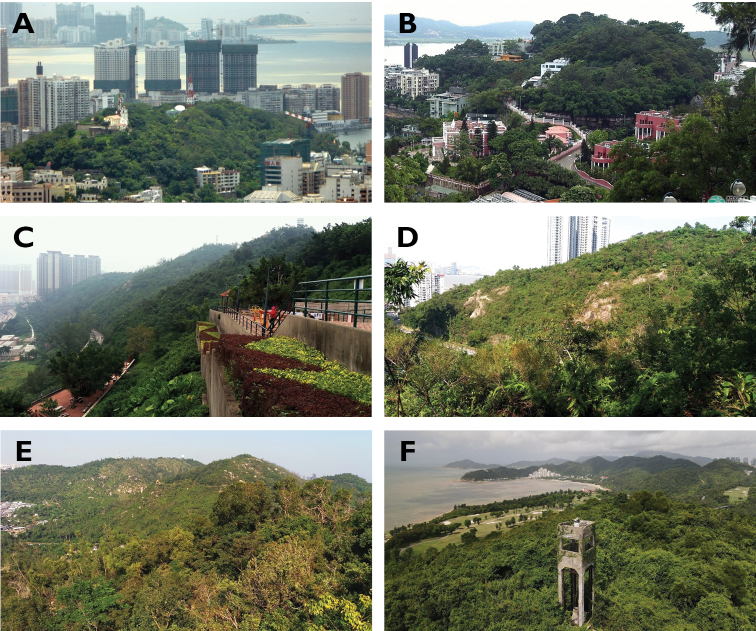
Examples of remaining pockets of subtropical evergreen forest in Macau **A** Guia Hill (Macau Peninsula) **B** Barra Hill (Macau Peninsula) **C** Great Taipa (Taipa) **D** Little Taipa (Taipa) **E** Coloane Heights (Coloane) **F** Ká-Hó Mountain (Coloane). Photographs: **A**Wikiwand.com**C** JTM.co.mo **B** culturalheritage.mo **C–E** LC **F** Hio Lou Chang.

Physically, the Macau SAR occupies a total area of ca. 30 km^2^ (Leong et al. 2017), which includes the Macau Peninsula, linked directly to the mainland province of Guangdong, and one larger island resulting from the merger of the two previously separated islands of Taipa (Cantonese: Tam Chai) and Coloane (Cantonese: Lou Wan) through the land reclaimed area of Cotai (Cantonese: Lou Tam) (Fig. 1). Land reclamation is an ongoing activity in the SAR, and since 1995 both the International Airport and the Hong Kong-Zhuhai-Macau Bridge Port have been added through this process to the Taipa-Coloane island complex and the Peninsula, respectively (Fig. 1). The remaining pockets of semi-natural landscape are often encroached upon by alien vegetation (Leong et al. 2017). They consist mainly of densely forested hilly outcrops intersected by networks of hiking trails, service roads and recreational facilities. The largest among the 18 areas identified are located in the Coloane area (e.g., Alto de Coloane, Barragem de Ká-Hó, and Monte de Ká-Hó) and Taipa (Taipa Grande and Taipa Pequena), but there are lesser pockets in the Peninsula as well (e.g., Colina da Guia, Colina da Barra, Parque Municipal de Mong Há, and Ilha Verde) (Figs 1, 2; cf. Direcção dos Serviços de Protecção Ambiental 2020). All these sites were visited on a regular basis during the census period, in order to provide an areal cover as comprehensive as possible of the potential habitats for longhorn beetles within the SAR.

Considering the exclusive either diurnal or nocturnal activity of most adult longhorn beetles, observations were undertaken during both daylight hours and at night. Flowers, dead trees and freshly cut branches were inspected during the hottest part of the day, in order to maximise potential encounters with beetles during their peak period of diurnal activity. At night, searches were limited to particularly brightly illuminated areas at the periphery of town, including street lights, public ablution blocks and monument spot-lights. Observations were made on an opportunistic basis from October 2017 and virtually on a daily basis during the period October 2018–May 2021. This generally involved non-manipulative methods, with photographs taken in situ as much as possible. No light traps were used, but the UV-based electric mosquito traps mounted by the authorities in each public ablution block were regularly inspected during the census. Where possible, electrocuted beetles were removed from the traps and preserved as voucher specimens for reference and identification verification purposes, along with other specimens retrieved already dead or moribund in the field. All specimens were analysed in detail and identified at the Key Laboratory of Zoological Systematics and Evolution of the Chinese Academy of Sciences in Beijing. Most of these specimens are now deposited in the National Zoological Museum of China, Institute of Zoology, Chinese Academy of Sciences (**IZCAS**, Beijing), while smaller collections are also housed in the Macau Anglican College (**MACT**, Taipa). Specimens from older collections housed in the Library of the University of Macau (Easton Collection, **UMEC**), at the Macau Municipal Affairs Bureau (“Collection de Instituto para os Assuntos Municipais” **CIAM**, Coloane) and at the Sun Yat-Sen University (**SYSU**, Guangzhou) were also included in the analysis.

Observations and data records were also obtained from the citizen science platform iNaturalist (https://www.inaturalist.org) and the following literature references: Easton (1991, 1992, 1993) and Pun and Batalha (1997).

As far as possible, photos of specimen dorsal and lateral habitus were taken in situ using a Nikon CoolPix S9700 digital camera with macro setting. However, when this was not practical in the field, specimens were photographed and measured under controlled conditions. Also, on rare occasions visual disturbances were removed from the photos using Microsoft Word 2010 (Picture Tools), in order to increase clarity and resolution of the images. All the species recorded during the census in the Macau SAR are illustrated with photos of live specimens in their natural or reconstructed setting, highlighting their key dorsal and, where possible, lateral characters. Specimen body length and maximum width were measured using a Vernier caliper, from the anterior margin of the mandibles to the apex of the pygidium and at the widest point of the elytra or pronotum, respectively. All measurements were approximated to the closest 0.5 mm. Within the text, only the original name, the essential synonyms and the currently recognised names are listed under each taxon, while for a comprehensive list of synonyms the reader is referred to the Catalogue of Chinese Coleoptera Volume IX, by Lin and Yang (2019), and the latest revision of the Catalogue of the Palaearctic Coleoptera, Volume 6/1, by Danilevsky (2020). The taxonomic structure used in these two catalogues is also followed in this work whenever an unresolved or controversial tribal or generic position exists, either in the literature or in the experts’ discussion forums. Type Locality (**TL**) and Type Depository (**TD**) are reported for each species along with their known distribution range, information on host plants, and other biological notes when available.

Public collections depositories of historical type material are abbreviated as follows:

**AMNH**American Museum of Natural History, New York, USA;

**NHMUK**Natural History Museum, London, United Kingdom;

**BPBM**Bernice Pauahi Bishop Museum, Honolulu, USA;

**CASF**California Academy of Sciences, San Francisco, USA;

**EMHU**Entomological Museum of Hokkaido University, Sapporo, Japan;

**LSLU**Linnean Society of London, London, United Kingdom;

**MNHN**Muséum national d’Histoire naturelle, Paris, France;

**MNLI**Museum für Naturkunde am Leibniz Institut für Evolutions und Biodiversitätsforschung, Berlin, Germany;

**NHRS**Naturhistoriska Riksmuseet, Stockholm, Sweden;

**NSMT**National Science Museum, Tokyo, Japan;

**OXUM** Hope Entomological Collections, University Museum, Oxford, United Kingdom;

**SFNF**Senckenberg Forschungsinstitut und Naturmuseum, Frankfurt, Germany;

**UZIU**Universitets Zoologiska Institutionen, Uppsala, Sweden;

**ZMUC**Zoologisk Museum Københavns Universitet, Copenhagen, Denmark.

## Results

### Historical and updated checklists

#### Gressitt (1951): 2 species

1) *Chlorophorus macaumensis* (Chevrolat)

2) Pterolophia (Hylobrotus) annulata (Chevrolat)

#### Easton (1991–1993): 10 species

1) *Anoplophora chinensis* (Forster)

2) *Batocera rubus* (Linnaeus)

3) *Imantocera penicillata* (Hope)

4) *Olenecamptus bilobus* (Fabricius)

5) *Aeolesthes induta* (Newman)

6) *Aristobia approximator* (Thomson)

7) *Pyrestes haematica* Pascoe

8) *Chelidonium sinense* (Hope)

9) *Chlorophorus annularis* (Fabricius)

10) *Xystrocera globosa* (Olivier)

#### Pun and Batalha (1997): 13 species

1) *Anoplophora chinensis* (Forster)

2) *Apriona germari* (Hope)

3) *Batocera horsfieldi* (Hope)

4) *Batocera rubus* (Linnaeus)

5) *Chlorophorus annularis* (Fabricius)

6) *Glenea cantor* (Fabricius)

7) *Imantocera penicillata* (Hope)

8) *Megopis marginalis* (Fairmaire)

9) *Oberea ferruginea* Thunberg

10) *Olenecamptus bilobus tonkinus* Dillon & Dillon

11) *Pothyne rugifrons* Gressitt

12) *Pterolophia annulata* (Chevrolat)

13) *Xystrocera globosa* (Olivier)

#### Lin and Yang (2019): 5 species

1) *Chlorophorus macaumensis* (Chevrolat)

2) Glenea (Stiroglenea) cantor (Fabricius)

3) *Pterolophia crassipes* (Wiedemann)

4) Pterolophia (Hylobrotus) annulata (Chevrolat)

5) *Purpuricenus temminckii sinensis* White

#### This Study, 2017–2021: 52 species

1) *Philus antennatus* (Gyllenhal, 1817)

2) *Philus pallescens pallescens* Bates, 1866

3) *Aegolipton marginale* (Fabricius, 1775)

4) *Cephalallus unicolor unicolor* (Gahan, 1906)

5) *Chelidonium argentatum* (Dalman, 1817)

6) *Embrikstrandia unifasciata* (Ritsema, 1896)

7) *Polyzonus sinensis* Hope, 1842

8) *Ceresium elongatum elongatum* Matsushita, 1933

9) *Ceresium longicorne* Pic, 1926

10) *Ceresium sinicum ornaticolle* Pic, 1907

11) *Ceresium zeylanicum* Yokoi, 2015

12) *Trirachys indutus* (Newman, 1842)

13) *Rhytidodera integra* Kolbe, 1886

14) *Chlorophorus annularis* (Fabricius, 1787)

15) *Chlorophorus macaumensis macaumensis* (Chevrolat, 1845)

16) *Demonax bimaculicollis* (Schwarzer, 1925)

17) *Perissus indistinctus* Gressitt, 1940

18) *Stromatium longicorne* (Newman, 1842)

19) *Kuegleria annulicornis* (Pic, 1935)

20) *Nysina rufescens asiatica* (Schwarzer, 1925)

21) *Pyrestes haematicus* Pascoe, 1857

22) *Purpuricenus temminckii sinensis* White, 1853

23) *Xystrocera globosa* (Olivier, 1795)

24) *Rondibilis undulata* (Pic, 1922)

25) *Apomecyna longicollis longicollis* Pic, 1926

26) *Apomecyna saltator* (Fabricius, 1787)

27) *Ropica dorsalis* Schwarzer, 1925

28) *Sybra marmorea* Breuning, 1939

29) *Sybra posticalis* (Pascoe, 1858)

30) *Batocera rubus rubus* (Linnaeus, 1758)

31) *Pseudoterinaea bicoloripes* (Pic, 1926)

32) *Sophronica apicalis* (Pic, 1922)

33) *Zotalemimon ciliatum* (Gressitt, 1942)

34) *Olenecamptus taiwanus* L.S. Dillon & D.S Dillon, 1948

35) *Exocentrus alboguttatus subconjunctus* Gressitt, 1940

36) *Exocentrus formosofasciolatus* Kusama & Tahira, 1978

37) *Bumetopia oscitans* Pascoe, 1858

38) *Coptops licheneus* (Pascoe, 1865)

39) *Anoplophora chinensis chinensis* (Forster, 1771)

40) *Blepephaeus subcruciatus* (White, 1858)

41) *Blepephaeus succinctor* (Chevrolat, 1852)

42) *Eutaenia tanoni* Breuning, 1962

43) *Monochamus alternatus alternatus* Hope, 1842

44) *Desisa subfasciata* (Pascoe, 1862)

45) *Mispila tholana* (Gressitt, 1940)

46) *Prosoplus bankii* (Fabricius, 1775)

47) *Pterolophia kaleea inflexa* Gressitt, 1940

48) *Pterolophia consularis* (Pascoe, 1866)

49) Pterolophia (Hylobrotus) annulata (Chevrolat, 1845)

50) Glenea (Stiroglenea) cantor cantor (Fabricius, 1787)

51) *Oberea ferruginea* (Thunberg, 1787)

52) *Oberea walkeri* Gahan, 1894

### Taxonomic account

#### Family VESPERIDAE Mulsant, 1839

**Subfamily Philinae J. Thomson, 1861**

##### Tribe Philini J. Thomson, 1861

###### Genus Philus

Taxon classificationAnimaliaColeopteraVesperidae

Saunders, 1853: 110.

B430FE89-AB51-5BBD-A6E0-CA5BABA139B0

####### Type species.

*Philus inconspicuus* Saunders, 1853 (= *Stenochorus antennatus* Gyllenhal, 1817).

###### Philus antennatus

Taxon classificationAnimaliaColeopteraVesperidae

(Gyllenhal, 1817)

39441A91-6394-5665-9EC9-8D018D1DFED9

Fig. 3

Stenochorus antennatus Gyllenhal, 1817: 180. TL: India (“orientali”); TD: NHRS

####### Distribution.

Palaearctic Region: China (Anhui, Fujian, Guangdong, Guangxi, Guizhou, Hainan, Hebei, Henan, Hong Kong, Hubei, Hunan, Jiangxi, Shaanxi, Shandong, Taiwan, Zhejiang) (Yiu 2009; Danilevsky 2020). Oriental Region: India (eastern) (Gressitt 1951; Švácha et al. 1997).

**Figure 3.**
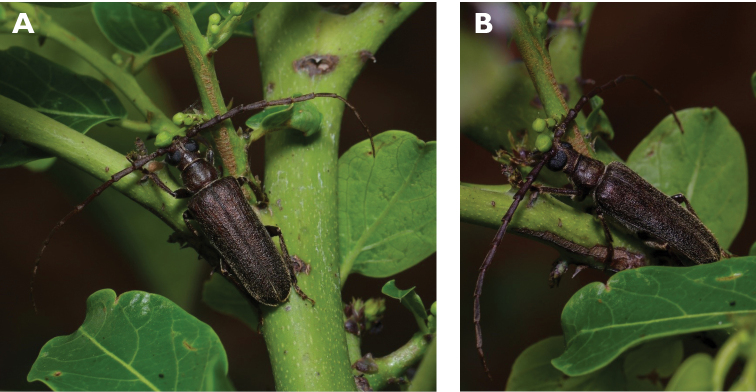
*Philus antennatus* (Gyllenhal, 1817): dorsal (**A**) and lateral (**B**) views of specimen observed in the Ká-Hó area of Coloane, on 1 May 2021 (photographs: Kisu Wong).

####### Macau records.

São Francisco Xavier, Ilhas [Coloane], 1 May 2021 8:37, Kisu Wong (https://www.inaturalist.org/observations/76970773); Coloane, Barragem de Ká-Hó, 1 May 2021 8:30, Wai Chan (https://www.inaturalist.org/observations/76102979).

####### Remarks.

Only two observations of male specimens from Macau could be found in the citizen science platform iNaturalist, but unfortunately the accompanying data did not contain any information about their size or habits. A third observation of a female specimen could not be confirmed due to the poor resolution of the photograph (https://www.inaturalist.org/observations/78005663). On Plate IV of Hua et al. (2009), the male represented in Fig. 40 is actually that of *Philus pallescens*, while the females in Fig. 40 and Fig. 41 represent the same specimen of *P. antennatus*. Unfortunately, their figure legends on p. 4 reflect this mistake. In Hong Kong, specimens attain a total length within the range of 24–31 mm (Yiu 2009). The mature larva of this species has been comprehensively described by Švácha et al. (1997) and feeds on the roots of a range of plants, including cultivated species such as *Citrus* spp., *Morus alba*, *Pinus elliottii*, and *P. taeda*, to which it can cause serious damage and death in young trees (Gressitt 1951; Chen et al. 1959; Švácha et al. 1997).

###### Philus pallescens pallescens

Taxon classificationAnimaliaColeopteraVesperidae

Bates, 1866

EC53CB31-BA8C-5EED-A70D-537389DE5A3C

Fig. 4

Philus pallescens Bates, 1866: 350. TL: China (Taiwan); TD: MNHN
Philus cantonensis Pic, 1930: 14. TL: China (“Canton”); TD: MNHN

####### Distribution.

Palaearctic Region: China (Fujian, Guangdong, Guangxi, Guizhou, Henan, Hong Kong, Hubei, Hunan, Inner Mongolia, Jiangsu, Jiangxi, Shaanxi, Sichuan, Taiwan, Zhejiang) (Lin and Yang 2019; Danilevsky 2020).

**Figure 4.**
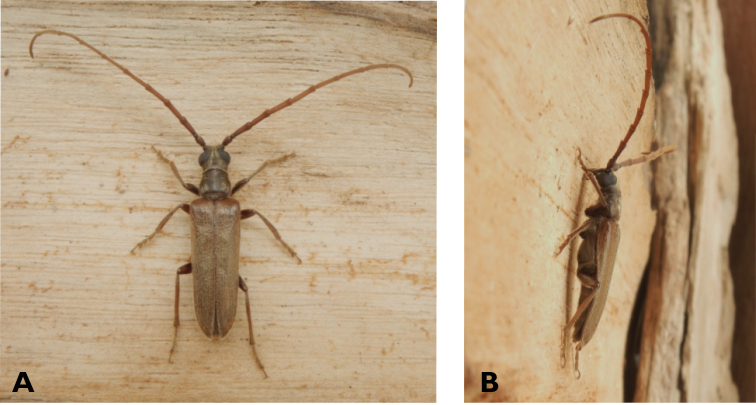
*Philus pallescens pallescens* Bates, 1866: dorsal (**A**) and lateral (**B**) views of specimen observed at Hác-Sá, Coloane, on 1 Jun 2020 (photographs: LC).

####### Macau records.

Coloane, Hác-Sá, crushed under street light, 5 May 2018, R Perissinotto & L Clennell; ibidem 10 May 2019, electrocuted inside UV mosquito trap of ablution block, R Perissinotto (IZCAS); ibidem 1 Jun 2020 outside ablution block, R Perissinotto.

####### Remarks.

The size of this species in Macau ranges 18–23 mm in total length and 5–7 mm in maximum width. In the Macau SAR, this species is very scarce and has only been recorded in late spring and always in the Hác-Sá area of Coloane. Like in all Vesperidae, the larvae are presumably subterranean, feeding on root sapwood and pupating within the soil (Švácha and Lawrence 2014). According to Hua (2002), host plants for the species include *Citrus* and *Saccharum sinensis*.

#### Family CERAMBYCIDAE Latreille, 1802

**Subfamily Prioninae Latreille, 1802**

##### Tribe Aegosomatini J. Thomson, 1861

###### Genus Aegolipton

Taxon classificationAnimaliaColeopteraCerambycidae

Gressitt, 1940: 22.

5A22F95A-1FBA-562C-9A71-726233C529DF

####### Type species.

*Cerambyx marginalis* Fabricius, 1775.

###### Aegolipton marginale

Taxon classificationAnimaliaColeopteraCerambycidae

(Fabricius, 1775)

87BAD658-74DA-517E-AADA-864D5EF70752

Fig. 5

Cerambyx marginalis Fabricius, 1775: 169. TL: “Cap Bonae Spei”; TD: NHMUK

####### Distribution.

Palaearctic Region: China (Anhui, Fujian, Guangdong, Guangxi, Guizhou, Hainan, Hong Kong, Hunan, Jiangsu, Jiangxi, Sichuan, Taiwan, Yunnan) (Yiu 2009; Lin and Yang 2019; Danilevsky 2020). Oriental Region: India; Indonesia (Java, Sumatra, Borneo-Kalimantan, Sulawesi, Ambon); Laos; Myanmar; Thailand; Vietnam (Kumawat et al. 2015).

**Figure 5.**
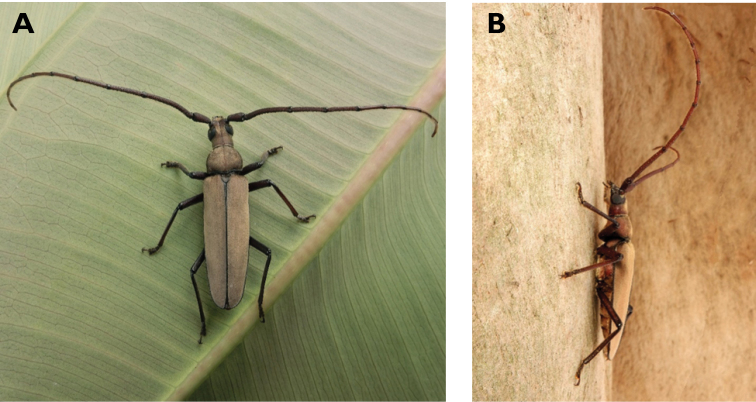
*Aegolipton marginale* (Fabricius, 1775): dorsal (**A**) and lateral (**B**) views of specimens observed on Guia Hill (12 May 2019) and Coloane Village (14 May 2020), respectively (photographs: LC).

####### Macau records.

1♂, Taipa, 1 Jun 1988, WW Pun, *Megopis marginalis* (CIAM); 1♀, Coloane, 26 Jul 1989, WW Pun, *Megopis marginalis* (CIAM); No data, “*Megopis marginalis* (Fairmaire), 毛角薄翅天牛28 mm” (Pun and Batalha 1997: 65, fig. 101); Taipa Grande, 2 May 2018, under street light, R Perissinotto & L Clennell; Macau, Guia Hill, 12 May 2019, under light in ablution block, R Perissinotto & L Clennell (IZCAS); Coloane Village, 14 May 2020, under light outside ablution block, R Perissinotto & L Clennell (MACT); Coloane, Caminho do Quartel de Hác-Sá, 5 May 2019 16:16, Jay Airosa (https://www.inaturalist.org/observations/28923060); Coloane, A-Má Goddess Statue, 16 May 2020 21:06, keanu83225 (https://www.inaturalist.org/observations/47820472); St. Francis Xavier’s Parish, [Coloane], 16 May 2020 21:20, Kisu Wong (https://www.inaturalist.org/observations/53851807); Our Lady of Carmel’s Parish [Great Taipa], 12 May 2021, Lynette Clennell (https://www.inaturalist.org/observations/78523372).

####### Remarks.

The size of this species in Macau ranges 28–38 mm in total length and 8–11 mm in maximum width. Like most prionines, this is an exclusively crepuscular and nocturnal species with activity in Macau restricted to the spring months. It is promptly attracted to artificial light, under which it often remains hidden throughout the daytime. Hua (2002) reported as host plants for this species *Casuarina equisetifolia*, *Cryptomeria fortunei*, *Eucalyptus exserta*, *Morus alba*, *Paulownia* sp., *Pinus* sp. and *Vernicia fordii*.

##### Subfamily Spondylidinae Audinet-Serville, 1832

###### Tribe Asemini J. Thomson, 1861

####### Genus Cephalallus

Taxon classificationAnimaliaColeopteraCerambycidae

Sharp, 1905: 148.

7F179EAF-4010-5497-A344-624CEA0FE7EC

######## Type species.

*Cephalallus oberthueri* Sharp, 1905.

####### Cephalallus unicolor unicolor

Taxon classificationAnimaliaColeopteraCerambycidae

(Gahan, 1906)

AB937A74-02E9-5D92-97F4-1757C5E5FF05

Fig. 6

Criocephalus unicolor Gahan, 1906: 97. TL: India (North Khasi Hills); TD: NHMUK.

######## Distribution.

Palaearctic Region: China (Fujian, Guangdong, Guizhou, Hainan, Henan, Hong Kong, Hubei, Hunan, Jiangsu, Jiangxi, Jilin, Sichuan, Taiwan, Yunnan, Zhejiang); Japan; Mongolia; North and South Korea (Yiu 2009; Lin and Yang 2019; Danilevsky 2020). Oriental Region: India (Assam); Laos; Myanmar (Kariyanna et al. 2017).

**Figure 6.**
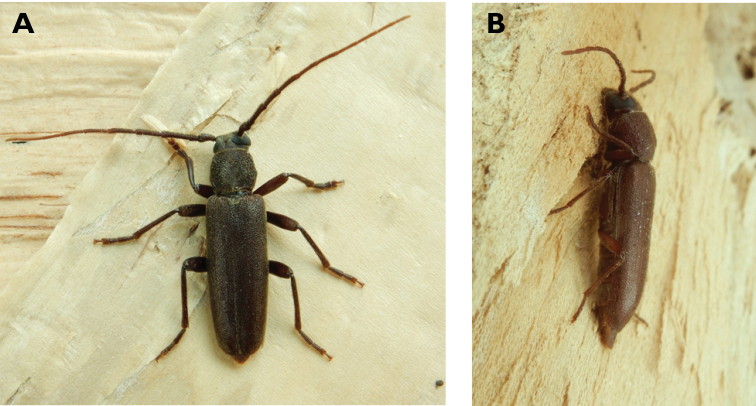
*Cephalallus unicolor unicolor* (Gahan, 1906): dorsal (**A**) and lateral (**B**) views of specimens observed on Great Taipa (11 Apr 2019) and Coloane Village (1 Apr 2020), respectively (photographs: LC).

######## Macau records.

Macau, University of East Asia, Block I, 17 Aug 1989, ER Easton leg (UMEC); ibidem Block F, 25 Apr 1991, ER Easton leg (UMEC); ibidem [no data], ER Easton leg (UMEC); Great Taipa, 11 Apr 2019, R Perissinotto; Coloane Heights, A-Má Statue, 1 Apr 2020, R Perissinotto; ibidem 9 Nov 2020, under spotlight, R Perissinotto (IZCAS); ibidem, A-Má Cultural Village, 17 May 2020, R Perissinotto & L Clennell (MACT); Coloane Village, 14 May 2020, under light in ablution block, R Perissinotto & L Clennell (MACT); [Taipa] Our Lady of Carmel’s Parish, 4 Apr 2020 22:59, Kit Chang (https://www.inaturalist.org/observations/41601849); ibidem 4 Apr 2020 21:15, Kisu Wong (https://www.inaturalist.org/observations/49550023); ibidem 23 Jul 2020 1:24, Kit Chang (https://www.inaturalist.org/observations/53971681); ibidem 3 Apr 2021 16:27, Lynette Clennell (https://www.inaturalist.org/observations/72763759); [Coloane] St. Francis Xavier’s Parish, 26 Apr 2020 23:52, Kisu Wong (https://www.inaturalist.org/observations/43868265); ibidem Apr 27 2020 12:45, Kit Chang (https://www.inaturalist.org/observations/43868614); ibidem 30 May 2020 1:22, Kit Chang (https://www.inaturalist.org/observations/47765485).

######## Remarks.

This species varies remarkably in size, from 12–21 mm in total length, to 3–5 mm in maximum width. In Macau, adults are active throughout the warmer parts of the year, from April till November. Larvae are reported to develop in pine trees, *Pinus* spp. (Yiu 2009; Lim et al. 2014) but have not been reported as causing damage to plantations or becoming invasive.

##### Subfamily Cerambycinae Latreille, 1802

###### Tribe Callichromatini Swainson & Shuckard, 1840

####### Genus Chelidonium

Taxon classificationAnimaliaColeopteraCerambycidae

J. Thomson, 1864: 175.

E01C6D75-75D3-5CD9-812E-8EFD3CA44E8C

######## Type species.

*Cerambyx argentatus* Dalman, 1817.

####### Chelidonium argentatum

Taxon classificationAnimaliaColeopteraCerambycidae

(Dalman, 1817)

26562AEB-2CCB-5CCB-888C-D9845489F785

Fig. 7

Cerambyx argentatus Dalman, 1817: 151. TL: Unknown; TD: NHRS

######## Distribution.

Palaearctic Region: China (Anhui, Chongqing, Fujian, Gansu, Guangdong, Guangxi, Hainan, Henan, Hong Kong, Hubei, Hunan, Jiangsu, Jiangxi, Ningxia, Sichuan, Shaanxi, Taiwan, Yunnan, Zhejiang); India (Sikkim) (Yiu 2009; Lin and Yang 2019; Danilevsky 2020). Oriental Region: India; Laos; Myanmar; Sri Lanka; Vietnam (Kariyanna et al. 2017).

**Figure 7.**
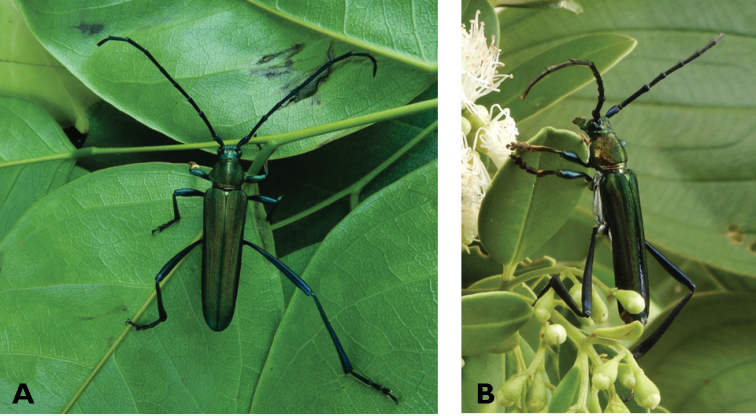
*Chelidonium argentatum* (Dalman, 1817): dorsal (**A**) and lateral (**B**) views of specimens observed on Coloane Heights (22 May 2019) and Coloane Village (22 May 2020), respectively (photographs: LC).

######## Macau records.

Taipa, University of East Asia Campus, 28 May 1992, on wall of Tai Fung building and 30 May 1992 outside classroom CLG 401, “*Chelidonium sinense* (Hope)” (Easton 1992: 35; Easton 1993: 47); Coloane, 30 May 2000, ML Lei (CIAM); Coloane, Cheoc-Van, 15 May 2019, on coastal vegetation, R Perissinotto; Coloane Heights, 22 May 2019, dead on path, R Perissinotto (IZCAS); ibidem 17 Jun 2020, on flowers of *Acronychia pedunculata*, R Perissinotto; ibidem 29 Jun 2020, R Perissinotto & L Clennell; Coloane Village, 22 May 2020, on flowers of *Psychotria serpens*, R Perissinotto (IZCAS); Coloane, Ká-Hó coast, 26 May 2020, on flowers of *Syzigium buxifolium*, R Perissinotto (MACT); ibidem 3 Jun 2020, R Perissinotto; Estrada do Alto de Coloane, 2 May 2021 9:45, jbsandsmacau (https://www.inaturalist.org/observations/77236125); St. Francis Xavier’s Parish [Coloane], 5 May 2021, Lynette Clennell (https://www.inaturalist.org/observations/77700701).

######## Remarks.

The size of this species in Macau ranges 24–31 mm in total length and 5–7 mm in maximum width. Adults are active during the hottest part of the day during May–June and have been observed while feeding on flowers of *Acronychia pedunculata*, *Psychotria serpens*, *Dalbergia benthamii*, and *Syzigium buxifolium* (RP pers. obs.). In nearby Hong Kong, larvae of this species have been recorded boring into the wood of citrus plants (Yiu 2009). More specifically, host plants include *Citrus aurantifolia*, *C. aurantium*, *C. limonia*, *C. microcarpa*, *C. reticulata* and *Fortunella margarita* (Duffy 1968; Makihara et al. 2008).

####### Genus Embrikstrandia

Taxon classificationAnimaliaColeopteraCerambycidae

Plavilstshikov, 1931: 278.

F9695D9D-7CA8-51EF-9AE6-5B037E6ED474

######## Type species.

*Callichroma bimaculatum* White, 1853.

####### Embrikstrandia unifasciata

Taxon classificationAnimaliaColeopteraCerambycidae

(Ritsema, 1896)

694A04BC-9170-5C9F-ABC4-8B0CA9C024A6

Fig. 8

Zonopterus unifasciatus Ritsema, 1896: 376. TL: Vietnam (Annam); TD: MNHN.

######## Distribution.

Palaearctic Region: China (Anhui, Fujian, Gansu, Guangdong, Guangxi, Hainan, Henan, Hong Kong, Hubei, Hunan, Jiangxi, Sichuan, Shanxi, Taiwan, Zhejiang); India (Sikkim) (Yiu 2009; Lin and Yang 2019; Danilevsky 2020). Oriental Region: India (Assam); Laos; Vietnam (Huang et al. 2006).

######## Macau records.

Coloane, Ká-Hó near lighthouse, 1 Jun 2020, on flowers of *Syzigium buxifolium*, R Perissinotto; Coloane, A-Mà Cultural Village, 17 Jun 2020, on flowers of *Acronychia pedunculata*, R Perissinotto (IZCAS); St. Francis Xavier’s Parish [Coloane], 20 May 2021, Lynette Clennell (https://www.inaturalist.org/observations/79506657).

######## Remarks.

**Figure 8.**
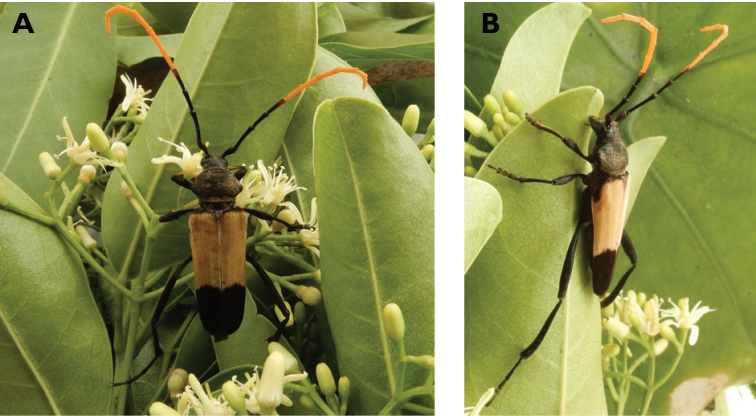
*Embrikstrandia unifasciata* (Ritsema, 1896): dorsal (**A**) and lateral (**B**) views of specimen observed on Coloane Heights on17 Jun 2020 (photographs: LC).

In Macau, this species has a total length of 21–27 mm and a maximum width of 6–8.5 mm. Three out of a total of four specimens observed during the study exhibit an expanded pale-yellow band across the elytra reaching all the way to the basal margin (Fig. 8), in a similar fashion to that shown by *Embrikstrandia vivesi* Bentanachs, 2005. However, the antennal segments 1–4 and all the legs are entirely black as is typical of *E. unifasciata*. Adult specimens appear to be active only in May–June and feed on flowers of *Syzigium buxifolium*, *Dalbergia benthamii* and *Acronychia pedunculata* during the hottest part of the day. In nearby Hong Kong, where this species has been erroneously reported as the related species *E. bimaculata* (White, 1853) (cf. Huang et al. 2006; Yiu 2009; Yiu and Yip 2011), larvae have been reported to bore into wood of *Zanthoxylum* sp. and *Tetradium glabrifolium* (Yiu 2009).

####### Genus Polyzonus

Taxon classificationAnimaliaColeopteraCerambycidae

Dejean, 1835: 324.

9DB71FBE-DFA0-5DDE-9AE4-26D94623EC57

######## Type species.

*Saperda fasciata* Fabricius, 1781

####### Polyzonus sinensis

Taxon classificationAnimaliaColeopteraCerambycidae

(Hope, 1842)

8F950DBE-8A47-51A2-9623-EBFA78122BC6

Fig. 9

Promeces sinensis Hope, 1842: 63. TL: China (Guangdong); TD: MNHN.

######## Distribution.

Palaearctic Region: China (Chongqing, Fujian, Guangdong, Guangxi, Guizhou, Hainan, Hong Kong, Hunan, Jiangxi, Jilin, Liaoning, Sichuan, Taiwan, Yunnan); India (Sikkim) (Lin and Yang 2019; Danilevsky 2020). Oriental Region: Laos; Myanmar; Thailand; Vietnam (Lin and Yang 2019).

######## Macau records.

Coloane, 20 May 1994, MW Ng (CIAM); Parque Natural de Taipa Grande, 24 May 2020 5:36, Wai Chan (https://www.inaturalist.org/observations/70479773); ibidem 8 May 2021 17:07, Kit Chang (https://www.inaturalist.org/observations/77868888); Great Taipa, 8 May 2021, perched on leaves on road margin, R Perissinotto & Lynette Clennell (IZCAS).

######## Remarks.

Easton (1993) reported this species as “*Chelidonium sinense* (Hope)” but it seems most likely that the main species involved in his observations was actually *C. argentatum* and not *Polyzonus sinensis*, given the laterally expanded metatibia and short tarsal segments exhibited by the typical specimen illustrated in his work (Easton 1993: 47). During the current census, *P. sinensis* was only observed on three occasions and always on Great Taipa Hill. The total length of these specimens varies between 23 and 26 mm, while their maximum width lies in the range of 5–6 mm. Adult specimens appear to have their peak of activity in May and have so far only been observed feeding on flowers of *Schima superba* during the hottest part of the day. Yiu (2009) reported that in Hong Kong the larvae of this species bore into *Citrus* plants and Hua (2002) also listed *Acacia* spp. as host plants in its broader distribution range.

**Figure 9.**
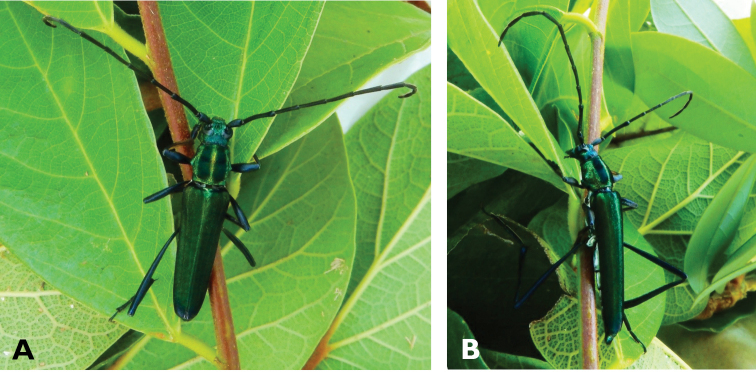
*Polyzonus sinensis* (Hope, 1842): dorsal (**A**) and lateral (**B**) views of specimen observed on Great Taipa Hill on 8 May 2021 (photographs: LC).

###### Tribe Callidiopini Lacordaire, 1868

####### Genus Ceresium

Taxon classificationAnimaliaColeopteraCerambycidae

Newman, 1842a: 322.

3BC52C99-A518-5C0D-BA55-2EEBA4C0DD47

######## Type species.

*Ceresium raripilum* Newman, 1842.

####### Ceresium elongatum elongatum

Taxon classificationAnimaliaColeopteraCerambycidae

Matsushita, 1933

695CF35F-0B21-51C4-9512-62153081623A

Fig. 10

Ceresium elongatum Matsushita, 1933: 301. TL: Japan (Okinawa); TD: EMHU

######## Distribution.

Palaearctic Region: China (Hong Kong, Taiwan); Japan (Ryukyus) (Yiu 2009; Lin and Yang 2019; Danilevsky 2020).

######## Macau records.

Great Taipa, 13 May 2019, under light in ablution block, R Perissinotto & L Clennell; Coloane Village, 19 May 2019, among flowers of *Psychotria serpens*, R Perissinotto & L Clennell (IZCAS); [Guia Hill] St. Lazarus’ Parish, 1 Jun 2020 22:36, Kit Chang (https://www.inaturalist.org/observations/48249964); ibidem 2 Jun 2020, Benny Kuok (https://www.inaturalist.org/observations/48308902); [Coloane] St. Francis Xavier’s Parish, 16 May 2020 21:25, Kit Chang (https://www.inaturalist.org/observations/46100618); ibidem 24 May 2020 23:14, Kit Chang (https://www.inaturalist.org/observations/47149856).

######## Remarks.

This species varies in the range of 10–14 mm in total length and 2–3 mm in maximum width. In Macau, adults appear to be active mainly during late spring, in May–June, and like those of the other species in this genus they are promptly attracted to artificial light during night-time, but are also occasionally seen during the day, hidden, and possibly feeding inside thick inflorescences. In Hong Kong, the larval stages of this species are known to develop within the wood of *Citrus* spp. and *Morus alba* (Yiu 2009).

**Figure 10.**
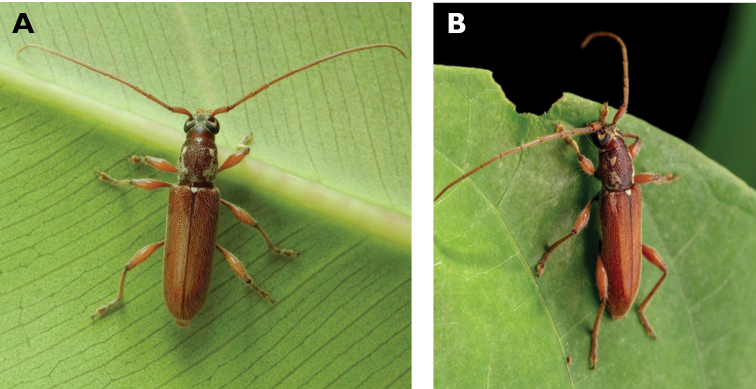
*Ceresium elongatum elongatum* Matsushita, 1933: dorsal (**A**) and lateral (**B**) views of specimens observed on Great Taipa Hill (13 May 2019) and on Guia Hill (2 Jun 2020), respectively (photographs: **A** LC **B** Benny Kuok).

####### Ceresium longicorne

Taxon classificationAnimaliaColeopteraCerambycidae

Pic, 1926

7697823A-A1E0-56E3-9A24-AD167C7EDA17

Fig. 11

Ceresium longicorne Pic, 1926: 24. TL: China (Taiwan); TD: MNHN

######## Distribution.

Palaearctic Region: China (Hong Kong, Hubei, Jiangxi, Taiwan); Japan; South Korea (Yiu 2009; Lin and Yang 2019; Danilevsky 2020).

######## Macau records.

1♀, Coloane, 11 Aug 1993, *Melia azedarach*, WW Pun (CIAM); Great Taipa, 1 Apr 2019, under light in ablution block, R Perissinotto & L Clennell (IZCAS); 1♀, Coloane Village, 19 May 2019, at light in ablution block, R Perissinotto & L Clennell (IZCAS); Coloane Heights, 24 May 2019, R Perissinotto; Coloane Village, 13 Jun 2020, under light in ablution block, R Perissinotto & L Clennell; [Coloane] St. Francis Xavier’s Parish, 2 May 2020 1:06, Kit Chang (https://www.inaturalist.org/observations/44572787); ibidem 24 May 2020 21:05, Kisu Wong (https://www.inaturalist.org/observations/54388846); ibidem 30 May 2021, Lynette Clennell (https://www.inaturalist.org/observations/81141600).

######## Remarks.

In Macau, adults of this species are active throughout the spring and are generally found in proximity to artificial lights at night. Their range in total length is 9–11 mm, and 1.5–3 in maximum width. In nearby Hong Kong, larvae of this species bore into *Citrus* spp. plants (Yiu 2009). Other larval host plants include *Diospyros kaki*, *Mallotus japonicas*, *Quercus acuta* (Lim et al. 2014), and in Japan even cultivated *Prunus salicina* (Kusigemati 1985).

**Figure 11.**
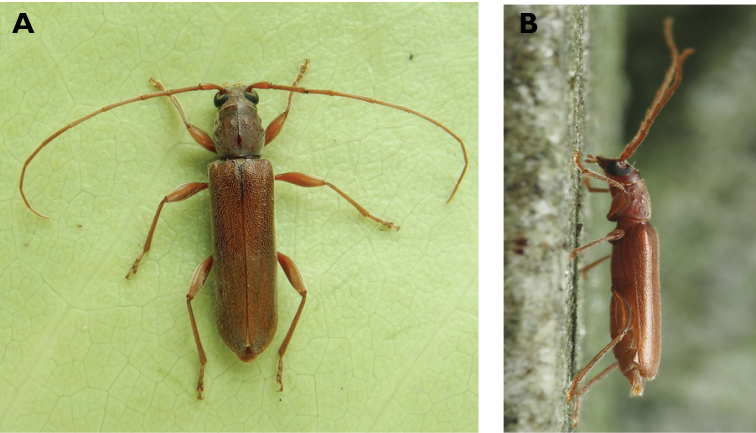
*Ceresium longicorne* Pic, 1926: dorsal (**A**) and lateral (**B**) views of specimens observed on Coloane Heights on 24 May 2019 and 2 May 2020, respectively (photographs: **A** LC **B** Kit Chang).

####### Ceresium sinicum ornaticolle

Taxon classificationAnimaliaColeopteraCerambycidae

Pic, 1907

38FDEAA1-22EF-5CF4-A8DA-D52AFE41078B

Fig. 12

Ceresium ornaticolle Pic, 1907: 20. TL: China (Yunnan); TD: MNHN.

######## Distribution.

Palaearctic Region: China (Fujian, Guangdong, Guangxi, Guizhou, Hong Kong, Hubei, Hunan, Jiangsu, Jiangxi, Shaanxi, Shanxi, Sichuan, Xizang, Yunnan, Zhejiang) (Yiu 2009; Lin and Yang 2019; Danilevsky 2020). Oriental Region: Laos; Vietnam (Gressitt and Rondon 1970).

**Figure 12.**
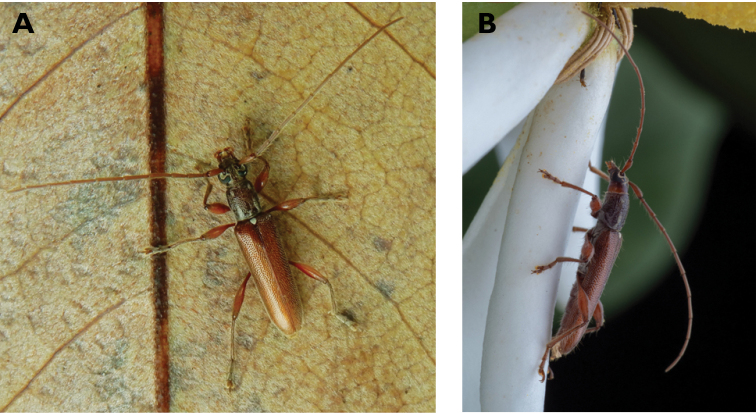
*Ceresium sinicum ornaticolle* Pic, 1907: dorsal (**A**) and lateral (**B**) views of specimens observed on Great Taipa (3 Mar 2019) and at the Hác-Sá Dam in Coloane (12 Apr 2020), respectively (photographs: **A** LC **B** Kit Chang).

######## Macau records.

Great Taipa, 3 Mar 2019, at light in ablution block, R Perissinotto & L Clennell; ibidem16 Mar 2019, R Perissinotto; ibidem 13 Jun 2019, R Perissinotto & L Clennell (IZCAS); Coloane Heights, 28 Apr 2019, at light in ablution block, R Perissinotto & L Clennell (IZCAS); Coloane Hác-Sá, 8 Apr 2020, on flowers of *Ligustrum sinense*, R Perissinotto & L Clennell); Coloane Ká-Hó, 22 May 2020, dead on tree trunk, R Perissinotto & L Clennell (IZCAS, MACT); Mong-Há Hill Municipal Park, 30 Apr 2019 22 :47, Eric Kwan (https://www.inaturalist.org/observations/24195774); [Coloane] Hác-Sá Dam, 21 Apr 2019 15:29, Kit Chang (https://www.inaturalist.org/observations/23059827); [Coloane] St. Francis Xavier’s Parish, 12 Apr 2020 21:33, Kit Chang (https://www.inaturalist.org/observations/48646082); ibidem 13 Apr 2020 21:50, Kisu Wong (https://www.inaturalist.org/observations/49577131); Taipa Grande, 12 May 2021, Lynette Clennell (https://www.inaturalist.org/observations/78523364).

######## Remarks.

In Macau, this species ranges 11–13 mm in total length and 2.5–3 mm in maximum width. During the current census it has been observed mainly at night under artificial lights, however on one occasion it was found during daytime feeding on flowers of *Ligustrum sinense*. In nearby Hong Kong, larvae have been documented to bore into wood of *Cinnamomum camphora*, *Citrus* spp., and *Melia azedarach* (Yiu 2009). Liu (1992) reported them as serious pests of *Punica granatum* in Sichuan, but also more generally of *Malus domestica*, *Pyrus* sp. and *Ricinus communis*.

####### Ceresium zeylanicum

Taxon classificationAnimaliaColeopteraCerambycidae

Yokoi, 2015

CC2F23D2-038E-563A-96AB-9A1C5150C8C2

Fig. 13

Ceresium zeylanicum Yokoi, 2015: 198. TL: Sri Lanka; TD: NHMUK.

######## Distribution.

Palaearctic Region: China (Hong Kong) (Yiu 2009; Lin and Yang 2019; Danilevsky 2020). Oriental Region: India; Myanmar; Philippines; Sri Lanka; Thailand; Laos; Vietnam (Kariyanna et al. 2017; Lin and Yang 2019).

######## Macau records.

Great Taipa, 6 May 2019, under light in ablution block, R Perissinotto (IZCAS); ibidem 16 May 2019, on flowers of *Lonicera japonica*, R Perissinotto; Coloane Village, 19 May 2019, at light in ablution block, R Perissinotto (IZCAS); ibidem 12 May 2020, R Perissinotto (MACT); ibidem 22 May 2020, R Perissinotto & L Clennell (IZCAS); [Coloane] St. Francis Xavier’s Parish, 10 May 2019 20:57, Hannah Leung (https://www.inaturalist.org/observations/27731651); ibidem 24 May 2020 23:22, Kit Chang (https://www.inaturalist.org/observations/47149883); ibidem 24 May 2020 22:20, Kisu Wong (https://www.inaturalist.org/observations/54388793); ibidem 23 Apr 2021, Lynette Clennell (https://www.inaturalist.org/observations/75004745); Coloane, Hác-Sá Reservoir, 1 May 2020 22:32, Eric Kwan (https://www.inaturalist.org/observations/44495900).

######## Remarks.

In Macau, adults are active only in the spring and range in total length 9.5–15 mm and 2–4 mm in maximum width. Although they have been found mainly around artificial lights at night, they have also been observed feeding on flowers of *Lonicera japonica* and *Gardenia jasminoides* during daytime (RP & LC pers. obs.). Larval host plants include *Artidesma tetrandrum*, *Bauhinia malabarica*, *Careya arborea*, *Heritiera minor*, *Lagerstroemia parviflora* and *Shorea robusta* (Duffy 1968; Makihara et al. 2008).

**Figure 13.**
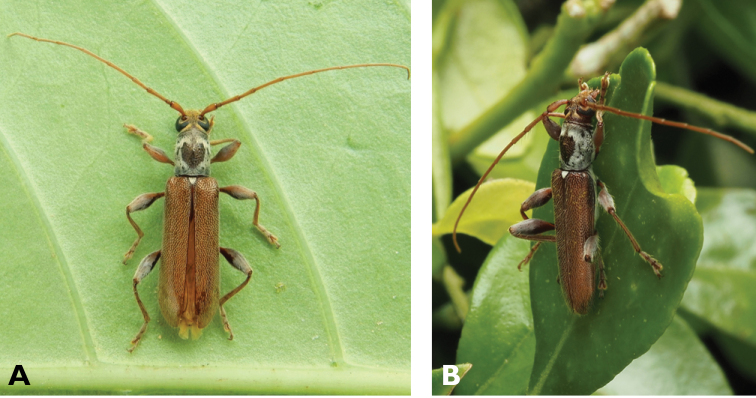
*Ceresium zeylanicum* Yokoi, 2015: dorsal (**A**) and lateral (**B**) views of specimens observed on Great Taipa (6 May 2019) and on Coloane Heights (24 May 2020), respectively (photographs: **A** LC **B** Kit Chang).

###### Tribe Cerambycini Latreille, 1802

####### Genus Trirachys

Taxon classificationAnimaliaColeopteraCerambycidae

Hope, 1843: 63.

F5391442-85F3-5F3F-9158-878ABA0EA9E8

######## Type species.

*Trirachys orientalis* Hope, 1843

####### Trirachys indutus

Taxon classificationAnimaliaColeopteraCerambycidae

(Newman, 1842)

C388B4CC-4C48-518E-8C8F-90CA757B3E38

Fig. 14

Hammaticherus indutus Newman, 1842b: 245. TL: Philippines (Luzon); TD: NHMUK

######## Distribution.

Palaearctic Region: China (Anhui, Fujian, Guangdong, Guangxi, Guizhou, Hainan, Hong Kong, Jiangxi, Taiwan, Zhejiang) (Yiu 2009; Lin and Yang 2019; Danilevsky 2020). Oriental Region: India; Indonesia (Sumatra, Java, Kalimantan); Laos; Malaysia; Myanmar; Philippines; Thailand; Sri Lanka; Vietnam (Makihara et al. 2008; Nga et al. 2014).

######## Macau records.

Taipa, University of East Asia Campus, 5 Apr 1992, on outside wall of Block I building (Easton 1992: 34); Macau, University of East Asia [no data], ER Easton leg (UMEC); ibidem [no data], ER Easton leg (UMEC); 1♀, Coloane, 16 Apr 1994, WW Tong (CIAM); 1♂, Coloane Village, 27 Apr 2020, under street light at night, R Perissinotto (IZCAS); 1♀, ibidem 19 Mar 2021, Lynette Clennell (https://www.inaturalist.org/observations/71677935).

######## Remarks.

This species was recorded only twice during the census and the specimens exhibited a total length of 30–37 mm and a maximum width of 8–10.5 mm. It has been reported previously from the region and from Hong Kong as *Aeolesthes induta* (Newman, 1842) (Easton 1992; Yiu 2009; Yiu and Yip 2011). Larval host plants include *Camellia thea*, *Chloroxylon swietenia*, *Delonix regia*, *Dracontomelon dao*, *Eugenia operculata*, *Hymenodictyon excelsum*, *Melia azedarach*, *M. japonica*, *Parashorea malayanonan*, *Pinus* sp., *Sapium sebiferum* and *Theobroma* sp. (Duffy 1968; Makihara et al. 2008; Yiu 2009).

**Figure 14.**
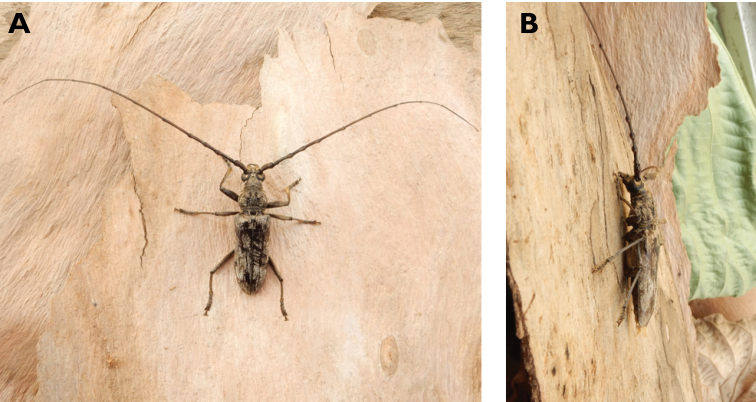
*Trirachys indutus* (Newman, 1842): dorsal (**A**) and lateral (**B**) views of specimen observed at Coloane Village on 27 Apr 2020 (photographs: LC).

####### Genus Rhytidodera

Taxon classificationAnimaliaColeopteraCerambycidae

White, 1853: 132.

4BADB53E-BA41-5274-A486-4A8D8048813D

######## Type species.

*Rhytidodera bowringii* White, 1853

####### Rhytidodera integra

Taxon classificationAnimaliaColeopteraCerambycidae

Kolbe, 1886

2CE068E6-7AE6-5493-B449-A4DF894923E1

Fig. 15

Rhytidodera integra Kolbe, 1886: 237. TL: Korea; TD: MNLI

######## Distribution.

Palaearctic Region: China (Fujian, Guangdong, Guangxi, Guizhou, Hainan, Henan, Hong Kong, Hubei, Hunan, Sichuan, Taiwan, Yunnan); South Korea (Yiu 2009; Lin and Yang 2019; Danilevsky 2020). Oriental Region: Laos; Myanmar; Thailand; Vietnam (Nga et al. 2014).

######## Macau records.

1♀, Coloane, 3 Jul 2000, ML Lei (CIAM); [Coloane] St. Francis Xavier’s Parish, 18 Jun 2020 21:53, Kit Chang (https://www.inaturalist.org/observations/50057385); Taipa, “Our Lady of Hope” Bay Wetland, 18 Jun 2020 23:36, Eric Kwan (https://www.inaturalist.org/observations/50069409); Macao Peninsula, Escola Luso-Chinesa Técnico-Profissional, 25 May 5:47, Wai Chan (https://www.inaturalist.org/observations/80195805).

######## Remarks.

The only specimen available in Macau collections exhibits a total length of 26 mm and a maximum width of 6 mm. Three other observations of this species from Macau were obtained from the citizen science platform iNaturalist, but unfortunately the accompanying data did not contain any information about their size or habits. In nearby Hong Kong, adults may attain a total length of 22–34 mm (Yiu 2009; Yiu and Yip 2011). Larvae are known to bore into wood of *Mangifera indica*, *Ficus microcarpa* and *F. retusa* (Yiu 2009; Lim et al. 2014).

**Figure 15.**
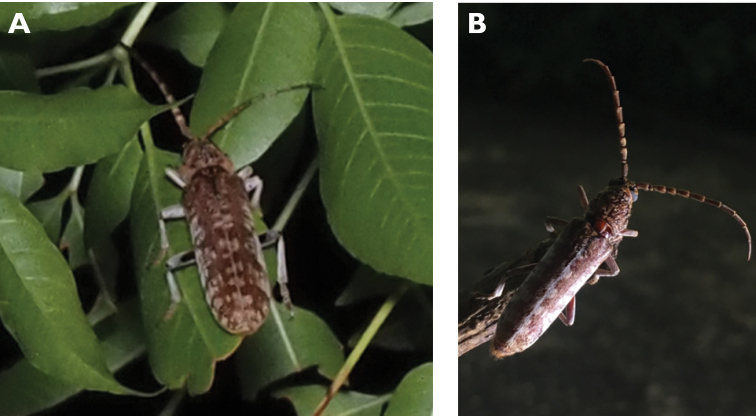
*Rhytidodera integra* Kolbe, 1886: Dorsal aspect of the two specimens observed at Coloane Heights and on the Taipa Bay Wetland on 18 Jun 2020 (photographs: **A** Kit Chang **B** Eric Kwan).

###### Tribe Clytini Mulsant, 1839

####### Genus Chlorophorus

Taxon classificationAnimaliaColeopteraCerambycidae

Chevrolat, 1863: 290.

1CEEA254-0385-546B-9045-C493455C01EA

######## Type species.

*Callidium annulare* Fabricius, 1787

####### Chlorophorus annularis

Taxon classificationAnimaliaColeopteraCerambycidae

(Fabricius, 1787)

07CF0D8A-9161-5CCA-B8D0-16366ECA221A

Fig. 16

Callidium annularis Fabricius, 1787: 156. TL: Thailand (“Siam”); TD: NHMUK.

######## Distribution.

Palaearctic Region: China (Anhui, Chongqing, Fujian, Guangdong, Guangxi, Guizhou, Hainan, Hebei, Henan, Hong Kong, Hubei, Hunan, Jiangsu, Jiangxi, Jilin, Liaoning, Shaanxi, Shanghai, Sichuan, Taiwan, Xizang, Yunnan, Zhejiang); Japan; Nepal; South Korea (Yiu 2009; Lin and Yang 2019; Danilevsky 2020). Oriental Region: Cambodia; India; Indonesia; Laos; Malaysia; Myanmar; Philippines; Sri Lanka; Thailand; Vietnam. Australian Region: Papua New Guinea; Australia. Pacific Region: Micronesia; USA (Hawaii). Also, widely introduced into Nearctic, Neotropical and Afrotropical regions (Makihara et al. 2008; Kariyanna et al. 2017; Danilevsky 2020).

######## Macau records.

Taipa, University of East Asia Campus, 28 May 1992 on outside wall of Tai Fung Building and 18 Jun 1992 near Library (Easton 1992: 35); no data, “*Chlorophorus annularis* (Fabricius), 竹綠虎天牛10 mm” (Pun and Batalha 1997: 64, fig. 98); 1 ♂, Cotai Ecological Zone, 2^nd^ zone, 6–7 Apr 2013, leg. Feng-Long Jia & Wei-Cai Xie (SYSU); Macau, Barra, 1 May 2019, on building wall, R Perissinotto & L Clennell (IZCAS); Coloane Heights, 30 May 2020, on flowers of *Acronychia pedunculata*, R Perissinotto & L Clennell; Coloane Village, 5 Jul 2020, on house window, R Perissinotto & L Clennell (MACT); Macau Cultural Centre, 12 Jun 2020 14:49, Eric Kwan (https://www.inaturalist.org/observations/49306503); University of Macau Campus, 28 Apr 2021 19:38, SS23 (https://www.inaturalist.org/observations/75876080); Coloane, 1 May 2021, Lynette Clennell (https://www.inaturalist.org/observations/76374764).

######## Remarks.

According to Easton (1992), *C. annularis*, or bamboo longhorn, was very common in Macau in the early 1990s, particularly during 1990 when it was suggested that it may have emerged from the numerous bamboo poles used in the scaffolding of new buildings that were being constructed next to the university campus. However, during this census the species was a rare occurrence in Macau, where adults were active in spring and summer and ranged 10–14 mm in total length and 2–3.5 mm in maximum width. *Chlorophorus annularis* is primarily a borer of dry bamboo species belonging to several genera, but it also attacks cultivated crops and wild plant species (Friedman et al. 2008). Both larvae and adults have been introduced into several European, Middle East, African, American and Oceanian countries through bamboo canes and their derived products imported from south-east Asian countries, especially China (Suma and Bella 2018).

The main larval host plants for the species include *Bambusa* spp., *Chimonobambusa tumidissinoda*, *Dendrocalamus strictus*, *Dipterocarpus tuberculatus*, *Cassia fistula*, *Gossypium* sp., *Indosasa crassiflora*, *Phyllostachys reticulata*, *Saccharum officinarum*, *Sinocalamus* spp., *Vitis* spp., and *Zea mays* (Friedman et al. 2008; Suma and Bella 2018). Other plants utilised to a lesser extent are *Albizia* spp., *Betula* spp., *Citrus* spp., *Derris microphylla*, *Liquidambar formosana*, *Malus sylvestris*, *Pyrus malus*, *Shorea robusta*, *Sinobambusa gibbosa*, *Spondias* sp. and *Tectona grandis* (Duffy 1968; Makihara et al. 2008; Yiu 2009; Lim et al. 2014; Suma and Bella 2018).

**Figure 16.**
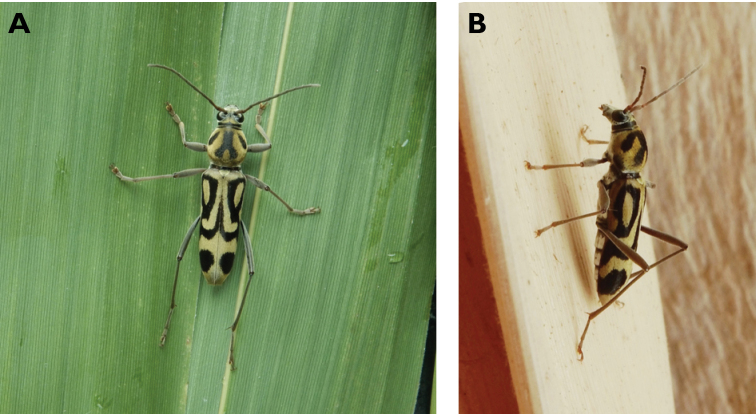
*Chlorophorus annularis* (Fabricius, 1787): dorsal (**A**) and lateral (**B**) views of specimens observed at Macau, Barra (1 May 2019) and Coloane Village (30 May 2020), respectively (photographs: LC).

####### Chlorophorus macaumensis macaumensis

Taxon classificationAnimaliaColeopteraCerambycidae

(Chevrolat, 1845)

137E0AE2-1BF5-5898-823F-D8A079DF4818

Fig. 17

Clytus macaumensis Chevrolat, 1845: 98. TL: China (Macau); TD: NHMUK.

######## Distribution.

Palaearctic Region: China (Guangdong, Guangxi, Hainan, Hong Kong, Hubei, Hunan, Shaanxi, Sichuan, Yunnan) (Yiu 2009; Lin and Yang 2019; Danilevsky 2020).

######## Macau records.

1♀, Coloane, 21 Jul 1988, Bambú, *Chlorophorus annularis*, WW Pun (CIAM); 1♀, ibidem 3 Jun 1994, *Chlorophorus annularis*, WW Pun (CIAM); 1♂, ibidem 21 May 1999, ML Lei (CIAM); 1♀, ibidem 14 Jun 2001, ML Lei (CIAM); Coloane Village, Jun 2018, L Clennell (MACT); Little Taipa, 28 Sep 2018, on roadside vegetation, R Perissinotto & L Clennell (MACT); Coloane Village, 28 Apr 2019, R Perissinotto & Clennell (IZCAS × 2); ibidem 20 Jun 2018, L Clennell; ibidem 1 Jul 2018, L Clennell (MACT × 2); ibidem 22 Jun 2019, L Clennell; Coloane Heights, 7 May 2020, R Perissinotto; ibidem 21 May 2020, R Perissinotto & L Clennell; ibidem 12 Jun 2020, numerous on flowers of *Acronychia pedunculata*, R Perissinotto & L Clennell (IZCAS); Macau, 27 May 2019, Kit Chang; ibidem 5 Jun 2019, Hannah Leung; St. Francis Xavier’s Parish [Coloane], 4 May 2019 10:51, Kit Chang (https://www.inaturalist.org/observations/24501575); ibidem 11 May 2019 15:28, Kit Chang (https://www.inaturalist.org/observations/24924416); ibidem 16 Jun 2019 15:59, Hannah Leung (https://www.inaturalist.org/observations/27731211); ibidem 24 May 2020 10:00, Kit Chang (https://www.inaturalist.org/observations/47084089); ibidem 20 Jun 2020 10:44, Kit Chang (https://www.inaturalist.org/observations/50238923); ibidem 9 May 2020 8:40, Kisu Wong (https://www.inaturalist.org/observations/52141600); ibidem 24 May 2020 11:54, Kisu Wong (https://www.inaturalist.org/observations/54257986); ibidem 21 Jun 2020 11:40, Kisu Wong (https://www.inaturalist.org/observations/56481171); ibidem 28 Jun 2020 9:30, Kisu Wong (https://www.inaturalist.org/observations/56944974); ibidem 19 Jul 2020 8:45, Kisu Wong (https://www.inaturalist.org/observations/58154540); ibidem 24 Apr 2021 11:52, Kit Chang (https://www.inaturalist.org/observations/75019281); ibidem 1 May 2021 13:12, Lynette Clennell (https://www.inaturalist.org/observations/76100053); Coloane Village, 9 May 2020 7:39, Lynette Clennell (https://www.inaturalist.org/observations/55370837); Coloane, Hác-Sá Dam, 31 May 2020 8:46, Annie Lao (https://www.inaturalist.org/observations/47961012); Taipa Pequena, 18 May 2021 11:47, Annie Lao (https://www.inaturalist.org/observations/79262176).

######## Remarks.

This is the only species that was found in reasonable abundance during the census, as shown by the extensive list of records above. Yet, remarkably it was not reported in the previous surveys by either Easton (1991, 1992, 1993) or Pun and Batalha (1997), despite Macau representing the type locality of the original description of the species by Chevrolat (1845). In Macau, adults are active during the hottest part of the day from spring till early autumn and range in total length 11–16 mm and 2.5–4 mm in maximum width. They feed on a wide variety of flowers, including *Acronychia pedunculata*, *Elaeocarpus sylvestris*, *Litsea glutinosa*, *Mallotus paniculatus*, *Paliurus spina-christi*, *Psychotria serpens*, *Dalbergia benthamii* and *Syzigium buxifolium* (RP & LC pers. obs.). Hua (2002) reported as host plants for this species *Acacia mearnsii*, *Bambusa textilis*, *Bauhinia variegata*, *Coffea* sp., *Cunninghamia lanceolata*, *Pinus* sp. (branches), *Populus* sp. and *Salix* sp.

**Figure 17.**
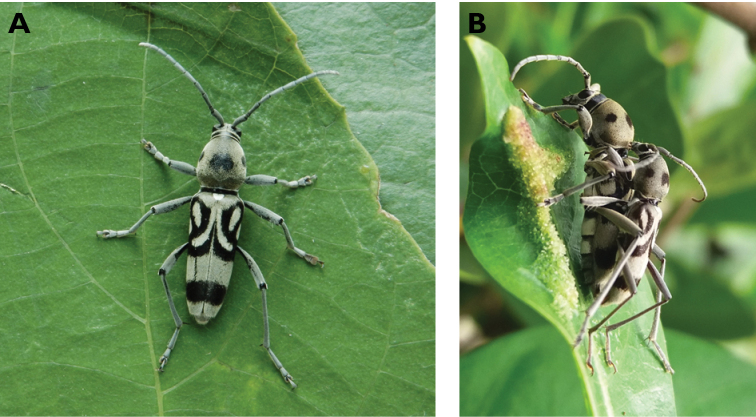
*Chlorophorus macaumensis macaumensis* (Chevrolat, 1845): dorsal (**A**) and lateral (**B**) views of specimens observed on Coloane Heights on 28 Apr 2019 and 21 May 2020, respectively (photographs: LC).

####### Genus Demonax

Taxon classificationAnimaliaColeopteraCerambycidae

J. Thomson, 1861: 226.

F1A9E508-A144-5FC2-8455-A4843B17800E

######## Type species.

*Demonax nigrofasciatus* J. Thomson, 1861

####### Demonax bimaculicollis

Taxon classificationAnimaliaColeopteraCerambycidae

(Schwarzer, 1925)

4FCB0790-7BD6-552D-98CC-9D94B358C89D

Fig. 18

Chlorophorus bimaculicollis Schwarzer, 1925a: 28. TL: China (Taiwan); TD: SFNF.

######## Distribution.

Palaearctic Region: China (Hainan, Taiwan) (Lin and Yang 2019; Danilevsky 2020).

######## Macau records.

Little Taipa, 4 Mar 2019, on flowers of *Ligustrum sinense*, R Perissinotto; ibidem 13 Mar 2019, on dead tree trunk by roadside, R Perissinotto (MACT); ibidem 25 Apr 2019, on flowers of *Mangifera* sp. by roadside, R Perissinotto & L Clennell (IZCAS).

######## Remarks.

In Macau, this species has so far only been recorded from Little Taipa Hill during March–April 1999 and ranges 8–11 mm in total length and 2–3 mm in maximum width. Adults appear to be active in daytime only during the early spring and have been observed mainly feeding on flowers of *Mangifera* sp. and occasionally also of *Ligustrum sinense*. Chou (2004, 2008) reported that adults visit flowers and leaves of *Acer cinnamomifolium*. Mating pairs and individuals near exit holes have also been found repeatedly on dead trunks of *Zanthoxylum avicennae*, indicating that this is possibly one of the host plants for larval development (RP pers. obs.).

**Figure 18.**
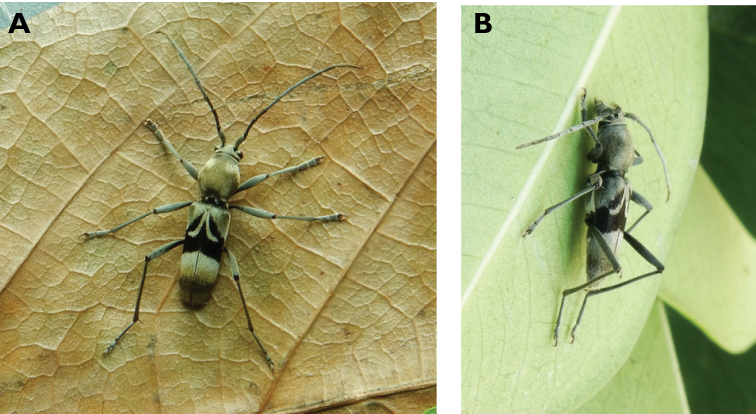
*Demonax bimaculicollis* (Schwarzer, 1925): dorsal (**A**) and lateral (**B**) views of specimens observed on Little Taipa Hill on 4 and 13 Mar 2019, respectively (photographs: LC).

####### Genus Perissus

Taxon classificationAnimaliaColeopteraCerambycidae

Chevrolat, 1863: 262.

99F2C6A8-CAC8-5A2C-B512-D28FEFF00EAA

######## Type species.

*Perissus x-littera* Chevrolat, 1863

####### Perissus indistinctus

Taxon classificationAnimaliaColeopteraCerambycidae

Gressitt, 1940

6E3B6736-686C-591C-A2F8-E1ACB5AD5B8F

Fig. 19

Perissus indistinctus Gressitt, 1940a: 72. TL: China (Hainan); TD: SYSU.

######## Distribution.

Palaearctic Region: China (Hainan, Hong Kong) (Lin and Yang 2019; Danilevsky 2020).

######## Macau records.

Coloane Heights, A-Mà Cultural Village, 12 Jul 2020, on dead tree trunk, R Perissinotto & L Clennell (IZCAS); ibidem 19 Nov 2020, Lynette Clennell (https://www.inaturalist.org/observations/65209287) (MACT); St. Francis Xavier’s Parish [Coloane], 15 Nov, 2020 15:48, Kit Chang (https://www.inaturalist.org/observations/64929674); ibidem 1 Apr 2021, Lynette Clennell (https://www.inaturalist.org/observations/72595959); ibidem 21 May 2021, Lynette Clennell (https://www.inaturalist.org/observations/79722351).

######## Remarks.

In Macau, adult activity has been recorded from early spring till late autumn. Specimens range 7–10.5 mm in total length and 1.5–3 mm in maximum width. Adults are active during the hottest part of the day and are generally observed on dead tree branches or roots, where they crawl back and forth with extreme rapidity searching for mates and areas suitable for egg deposition. No information seems to be available in the literature on the larval food plants.

**Figure 19.**
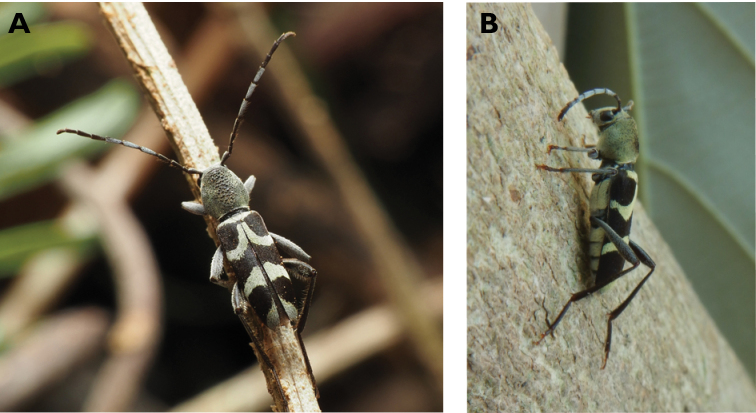
*Perissus indistinctus* Gressitt, 1940: dorsal (**A**) and lateral (**B**) views of specimens observed on Coloane Heights on 15 Nov 2020 and 12 Jul 2020, respectively (photographs: **A** Kit Chang **B** LC).

###### Tribe Hesperophanini Mulsant, 1839

####### Genus Stromatium

Taxon classificationAnimaliaColeopteraCerambycidae

Audinet-Serville, 1834: 80.

4173422D-BE5A-5963-B25F-1792B40407E6

######## Type species.

*Callidium barbatum* Fabricius, 1775

####### Stromatium longicorne

Taxon classificationAnimaliaColeopteraCerambycidae

(Newman, 1842)

AD6A8B3A-4326-51A7-98DF-C8970A7C805C

Fig. 20

Arhopalus longicornis Newman, 1842a: 246. TL: Philippines (Manila); TD: NHMUK
Stromatium asperulum White, 1855: 300. TL: China (Hong Kong); TD: NHMUK

######## Distribution.

Palaearctic Region: China (Fujian, Guangdong, Guangxi, Guidzou, Hainan, Hong Kong, Inner Mongolia, Jiangxi, Jilin, Liaoning, Shandong, Taiwan, Yunnan, Zhejiang); India (north); Japan; Nepal; (Yiu 2009; Lin and Yang 2019; Danilevsky 2020). Oriental Region: India (Assam); Indonesia (Kalimantan, Sunda Islands); Laos; Malaysia; Myanmar; Thailand; Vietnam (Hua 2002; Nga et al. 2014). Nearctic Region: USA (intercepted) (Monné and Giesbert 1994).

######## Macau records.

Great Taipa, 6 Jun 2019, on floor in ablution block, R Perissinotto & L Clennell (IZCAS); Coloane Village, 29 May 2020, on mosquito trap of ablution block, R Perissinotto & L Clennell (IZCAS); ibidem 1 Jun 2020, R Perissinotto & L Clennell (MACT); Taipa, Minho Str., 23 May 2020 19:58, Eric Kwan (https://www.inaturalist.org/observations/46988050); Taipa, Pac On Road, 28 May 2020 22:20, Eric Kwan (https://www.inaturalist.org/observations/47699525); Coloane Village, 20 May 2021, Lynette Clennell (https://www.inaturalist.org/observations/79509103).

######## Remarks.

In Macau, adults appear to be active only in late spring and range in total length 23–28 mm and 6.5–8 mm in maximum width. The species is exclusively nocturnal and readily attracted to artificial lights. The larvae apparently bore into lumber and a variety of trees, such as *Machilus* spp., *Morus alba* and oaks (Yiu 2009). The species was introduced into Australia already in the 1960s (Duffy 1963) and adult specimens emerging from wood furniture and other processed timber have recently been intercepted in several European countries (Cocquempot et al. 2014).

**Figure 20.**
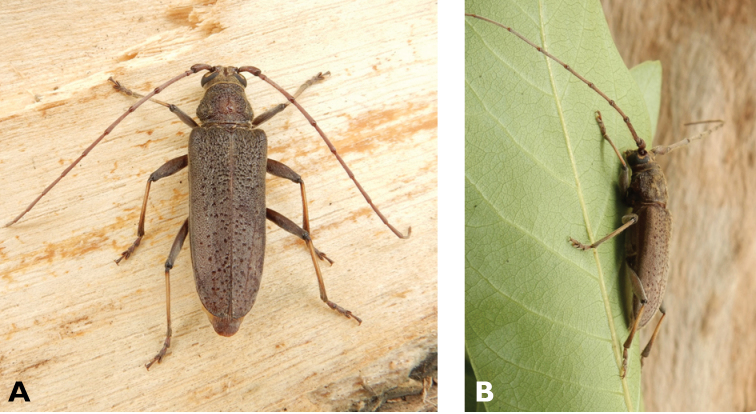
*Stromatium longicorne* (Newman, 1842): female dorsal (**A**) and male lateral (**B**) views of specimens observed at Coloane Village on 29 May 2020 (photographs: LC).

###### Tribe Obriini Mulsant, 1839

####### Genus Kuegleria

Taxon classificationAnimaliaColeopteraCerambycidae

Holzschuh, 2017: 13.

94D5A9F4-BAFB-5EF6-A9DD-5081C8F59F7C

######## Type species.

*Obrium atricolor* Pic, 1953.

####### Kuegleria annulicornis

Taxon classificationAnimaliaColeopteraCerambycidae

(Pic, 1935)

C7B5FB31-35D1-5965-BEAF-E79432E27EF5

Fig. 21

Falsobrium annulicorne Pic, 1935: 13. TL: Vietnam (Tonkin); TD: MNHN

######## Distribution.

Palaearctic Region: China [Hong Kong, new record: 1♂, Shing Mun, 24 May 2010, Atwood Chiu (on loan to IZCAS by V Yiu, Accession No. CO160601)]. Oriental Region: Laos; Vietnam (Holzschuh 2017).

######## Macau records.

Great Taipa, 9 May 2019, on pile of dead wood in barbeque area, R Perissinotto & L Clennell (IZCAS); ibidem 7 May 2021, [in ablution block], Lynette Clennell (https://www.inaturalist.org/observations/77993530).

######## Remarks.

This species represents a new record for China and the broader Palaearctic Region. The two specimens recorded during this survey exhibit a total length of 6–7 mm and a maximum width of 1–1.5 mm. One specimen was active during daytime, flying above a pile of dead wood, while the second specimen was recovered from an ablution block, where it had likely been attracted by artificial lights during the night.

**Figure 21.**
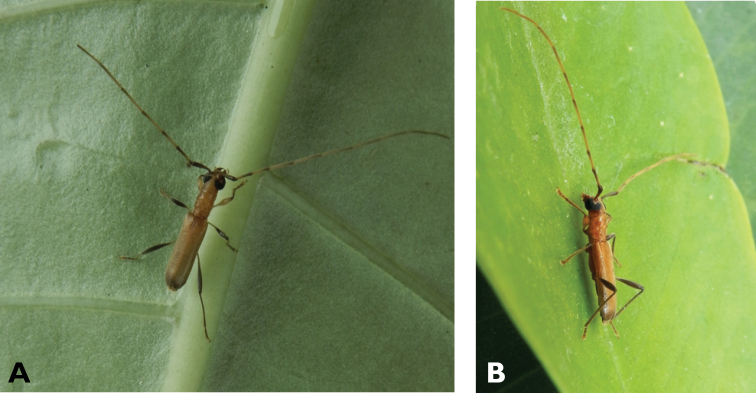
*Kuegleria annulicornis* (Pic, 1935): dorsal (**A**) and lateral (**B**) views of specimen observed on Great Taipa Hill on 9 May 2019 (photographs: LC).

###### Tribe Phoracanthini Newman, 1840

####### Genus Nysina

Taxon classificationAnimaliaColeopteraCerambycidae

Gahan, 1906: 153.

1EDC7626-475C-53A0-BE2A-12F07F196F5B

######## Type species.

*Sphaerion orientale* White, 1853.

####### Nysina rufescens asiatica

Taxon classificationAnimaliaColeopteraCerambycidae

(Schwarzer, 1925)

589C215E-8383-59BA-A050-6D4CBD8ECBF6

Fig. 22

Neosphaerion asiaticum Schwarzer, 1925a: 22. TL: China (Taiwan); TD: SFNF

######## Distribution.

Palaearctic Region: China (Fujian, Guangxi, Hainan, Hong Kong, Taiwan, Zhejiang) (Yiu 2009; Lin and Yang 2019; Danilevsky 2020). Oriental Region: Vietnam (Lin and Yang 2019).

######## Macau records.

Coloane Heights, 5 Jul 2019, feeding on unidentified flower in garden, R Perissinotto & L Clennell (IZCAS); ibidem 10 May 2020, R Perissinotto & L Clennell (IZCAS); Coloane Village, 28 Jun 2020, on mosquito trap in ablution block, R Perissinotto & L Clennell (IZCAS); Great Taipa, 21 Mar 2019, inside mosquito trap, R Perissinotto (MACT); ibidem 1 Mar 2020, Kit Chang; Guia Hill, 7 Mar 2020, Kit Chang; St. Francis Xavier’s Parish [Coloane], 27 Apr 2020 12:24, Kit Chang (https://www.inaturalist.org/observations/43868608); ibidem 19 Apr 2020 23:45, Kisu Wong (https://www.inaturalist.org/observations/51105296); ibidem 22 Mar 2021, Lynette Clennell (https://www.inaturalist.org/observations/71851567); Our Lady of Carmel’s Parish [Little Taipa], 1 Mar 2020 23:30, Kit Chang (https://www.inaturalist.org/observations/48545850; ibidem 29 Mar 2021, Lynette Clennell (https://www.inaturalist.org/observations/72418305).

######## Remarks.

In Macau, adults are active throughout the spring and summer, both during the day feeding on flowers and at night when they are attracted to artificial lights. They range in total length 10.5–13 mm and 2.5–3 mm in maximum width. There appears to be no information available in the literature on the larval food plant(s) of this species.

**Figure 22.**
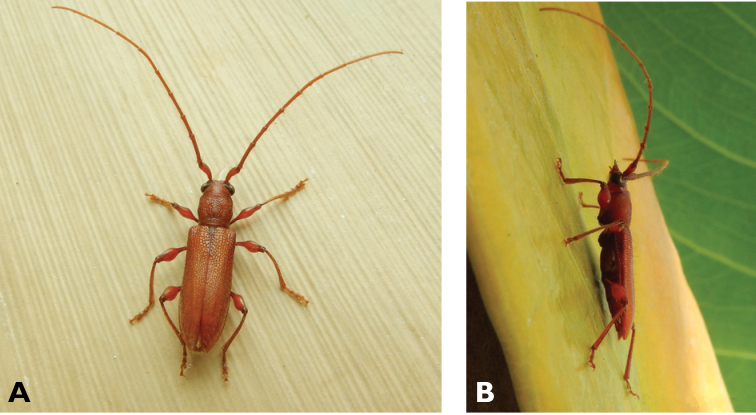
*Nysina rufescens asiatica* (Schwarzer, 1925): dorsal (**A**) and lateral (**B**) views of specimens observed on Coloane Heights (5 Jul 2019) and at Coloane Village (28 Jun 2020), respectively (photographs: LC).

###### Tribe Pyrestini Lacordaire, 1868

####### Genus Pyrestes

Taxon classificationAnimaliaColeopteraCerambycidae

Pascoe, 1857: 96.

E32A33C3-BEC1-57C7-B129-5D592F45CA84

######## Type species.

*Pyrestes haematicus* Pascoe, 1857.

####### Pyrestes haematicus

Taxon classificationAnimaliaColeopteraCerambycidae

Pascoe, 1857

2E13D4E3-1574-5058-BE7D-0FBEFADC81B2

Fig. 23

Pyrestes haematicus Pascoe, 1857: 97. TL: China (North); TD: NHMUK
Pyrestes cardinalis Pascoe, 1863: 50. TL: China (Hong Kong); TD: NHMUK. Synonymised by Gressitt 1939: 31.

######## Distribution.

Palaearctic Region: China (Anhui, Fujian, Guangdong, Guizhou, Hainan, Henan, Hong Kong, Hubei, Hunan, Jiangsu, Jangxi, Shaanxi, Taiwan, Yunnan, Zhejiang); North & South Korea (Yiu and Yip 2011; Lin and Yang 2019; Danilevsky 2020).

######## Macau records.

Taipa, University of East Asia Campus, 28 March & 24 April on wall of Block I building, “*Pyrestes haematica* Pascoe” (Easton 1992: 34); Coloane, Cheoc Van, 16 May 2019, perched on coastal vegetation, R Perissinotto & L Clennell (IZCAS); Coloane Heights, 18 Jun 2020, on flowers of *Acronychia pedunculata* in garden, R Perissinotto & L Clennell (MACT, IZCAS); ibidem 3 Jul 2020, on flowers of *Elaeocarpus sylvestris*, R Perissinotto; Coloane, 20 May 2021, Lynette Clennell (https://www.inaturalist.org/observations/79506655).

######## Remarks.

In Macau, adults are active only during late spring to early summer and specimens range in total length 10–16 mm and 2–4 mm in maximum width. They feed on a variety of flowers during the hottest part of the day, including *Acronychia pedunculata*, *Dalbergia benthamii* and *Elaeocarpus sylvestris*. Known larval food plants include *Cinnamomum camphora*, *Machilus* spp. and *Pueraria lobata* (Lim et al. 2014).

**Figure 23.**
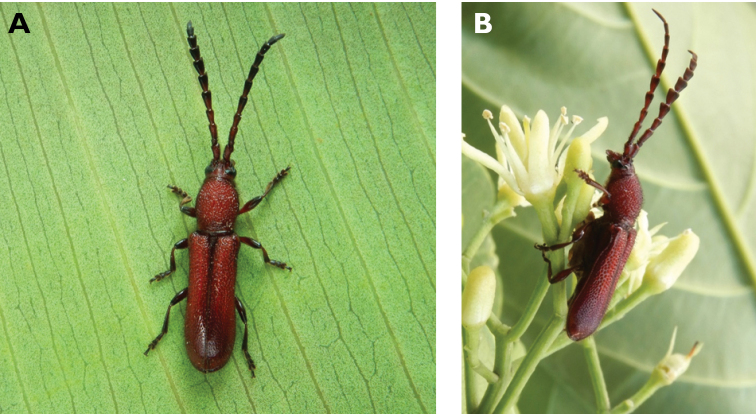
*Pyrestes haematicus* Pascoe, 1857: dorsal (**A**) and lateral (**B**) views of specimens observed at Coloane, Cheoc-Van (16 May 2019) and on Coloane Heights (18 Jun 2020), respectively (photographs: LC).

###### Tribe Trachyderini Dupont, 1836

####### Genus Purpuricenus

Taxon classificationAnimaliaColeopteraCerambycidae

Dejean, 1821: 105.

7070027E-A5B2-5EB7-97E0-285EFA1C0AA5

######## Type species.

*Cerambyx kaehleri* Linnaeus, 1758

####### Purpuricenus temminckii sinensis

Taxon classificationAnimaliaColeopteraCerambycidae

White, 1853

5E5BE4FC-F7CD-517F-9109-841D5A4A85E1

**Figure 24.**
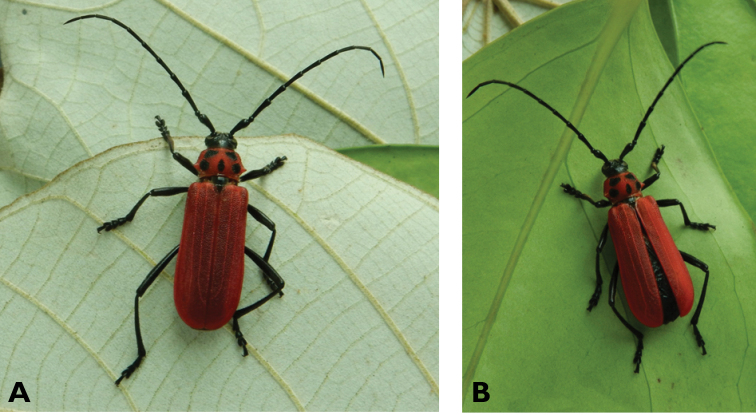
*Purpuricenus temminckii sinensis* White, 1853: Dorsal views of specimen observed at Taipa on 19 Mar 2019 (photographs: LC).

Fig. 24
Purpuricenus sinensis White, 1853: 139. TL: China (Shanghai); TD: NHMUK.

######## Distribution.

Palaearctic Region: China (Fujian, Guangdong, Guangxi, Guizhou, Hainan, Hebei, Henan, Hong Kong, Hubei, Hunan, Jiangsu, Jiangxi, Liaoning, Shaanxi, Shandong, Shanghai, Shanxi, Sichuan, Taiwan, Yunnan, Zhejiang); India (Arunachal Pradesh); South Korea (Yiu 2009; Lin and Yang 2019; Danilevsky 2020). Oriental Region: Laos; Vietnam (Ambrus and Tichý 2017).

######## Macau records.

Taipa Central, 19 Mar 2019, flying near bus stop, R Perissinotto.

######## Remarks.

The only specimen observed in Macau had a total length of 16.5 mm and a maximum width of 5.5 mm. Yiu (2009) reported that the larval stages develop in bamboo canes and jujube trees. This is supported by Hua (2002), who listed *Bambusa* and *Ziziphus sativa* as host plants of this species.

###### Tribe Xystrocerini Blanchard, 1845

####### Genus Xystrocera

Taxon classificationAnimaliaColeopteraCerambycidae

Audinet-Serville, 1834: 69.

574DA1A1-A5E2-58F8-8AD6-CF6C51EACDEE

######## Type species.

*Cerambyx globosus* Olivier, 1795; designated by Thomson 1864: 247.

####### Xystrocera globosa

Taxon classificationAnimaliaColeopteraCerambycidae

(Olivier, 1795)

3D574F65-2ED2-575F-BB97-96BD111698CC

Fig. 25

Cerambyx globosus Olivier, 1795: 27, pl. XII, fig. 81. TL: Indonesia (“Batavia”); TD: Unknown.

######## Distribution.

Palaearctic Region: Bhutan; China (Anhui, Chongqing, Fujian, Gansu, Guangdong, Guangxi, Guizhou, Hainan, Hebei, Henan, Hong Kong, Hubei, Hunan, Jiangsu, Jiangxi, Taiwan, Shaanxi, Shandong, Sichuan, Yunnan, Zhejiang); Egypt; India (Arunachal Pradesh, Sikkim, Uttarakhand); Israel (introduced); Japan; Nepal; North & South Korea; Pakistan (Yiu 2009; Lin and Yang 2019; Danilevsky 2020). Oriental Region: Bangladesh; Cambodia; India; Indonesia; Laos; Malaysia; Myanmar; Philippines; Sri Lanka; Thailand; Vietnam (Hua 2002; Kariyanna et al. 2019). Also widely distributed in Afrotropical Region (Africa), Nearctic Region (North America) as well as Australian and Pacific regions (Oceania) (Danilevsky 2020).

######## Macau records.

Taipa, University of East Asia Campus, 9 Apr & 5 May 1992, on outside wall of Block I building, (Easton 1992: 35); Macau, University of East Asia, Taipa [no date], ER Easton leg (UMEC); ibidem Block F, 9 Apr 1990, ER Easton leg (UMEC); no data, “*Xystrocera globosa* (Olivier), 合歡雙條天牛27 mm” (Pun and Batalha 1997: 65, fig. 106); 1♀, Coloane, 2 Apr 1993, *Albizia chinensis*, WM Ng, *Xystrocera globosa* (CIAM); 1♂, ibidem 16 Oct 1993, *Xystrocera globosa*, WW Pun (CIAM); Taipa Central, 18 Mar 2019, on building wall, R Perissinotto; Little Taipa, 28 Apr 2019, under monument spotlight, R Perissinotto & L Clennell (IZCAS); ibidem 1 Sep 2019, R Perissinotto & L Clennell (MACT); Coloane Village, 6 Oct 2019, L Clennell; Coloane Heights 7 Mar 2020, on trunk of *Albizia lebbeck*, R Perissinotto; ibidem 26 Mar 2020, R Perissinotto; ibidem 11 Apr 2020, R Perissinotto & L Clennell; ibidem 21 Apr 2020, on trunk of *Albizia lebbeck*, R Perissinotto; ibidem 28 Aug 2020, under spotlight, R Perissinotto & L Clennell (IZCAS); ibidem 4 Sep 2019, R Perissinotto & L Clennell (MACT); Great Taipa, 4 Apr 2020 20:57, Eric Kwan (https://www.inaturalist.org/observations/41422999); ibidem 4 Apr 2020 20:55, Kisu Wong (https://www.inaturalist.org/observations/49550012); ibidem 4 Apr 2020 21:02, Kit Chang (https://www.inaturalist.org/observations/48643480); ibidem 12 Mar 2021, Lynette Clennell (https://www.inaturalist.org/observations/71056521); Coloane, Tin Hau Temple, 26 Apr 2020 23:55, Eric Kwan (https://www.inaturalist.org/observations/43840228); St. Francis Xavier’s Parish [Coloane], 5 Oct 2019 16:24, Lynette Clennell (https://www.inaturalist.org/observations/56122495); ibidem 26 Apr 2020 23:32, Kisu Wong (https://www.inaturalist.org/observations/43868252); ibidem 4 Apr 2021 13:49, Lynette Clennell (https://www.inaturalist.org/observations/72875264); [Macau Peninsula] Jardim de Lou Lim Loc, 5 Apr 2021 10:46, Eric Kwan (https://www.inaturalist.org/observations/72982387).

######## Remarks.

In Macau, adults are active from early spring till mid-autumn and range in total length 24–30.5 mm and 5–8 mm in maximum width. In the Coloane area, adults have repeatedly been observed while emerging from exit holes on dead or moribund trunks of *Albizia lebbeck*. In nearby Hong Kong, host plants for this species include *Acacia confusa*, *Albizia lebbeck*, *Bauhinia* spp. and *Bombax ceiba* (Yiu 2009). Elsewhere, the following additional species have also been recorded: *Acacia arabica*, *A. catechu*, *A. chinensis*, *A. cordifolia*, *A. modesta*, *A. auriculoformis*, *A. mangium*, *Acrocarpus fraxinifolius*, *Adenanthera pavonina*, *Albizia julibrissin*, *A. lucida*, *A. odoratissima*, *A. procera*, *A. stipulata*, *Bauhinia acuminata*, *Cassia glauca*, *Chamaecrista* spp., *Duabanga sonneratioides*, *Grewia parviflora*, *G. tiliaefolia*, *Haematoxylon campechianum*, *Paraserianthes falcataria*, *Parkia speciosa*, *Prunus persica*, *Salmalia malabarica*, *Samanea samon*, *Theobroma* spp., *Xylia dolabriformis* and *X. xylocarpa* (Duffy 1968; Matsumoto et al. 2000; Makihara et al. 2008; Lim et al. 2014).

**Figure 25.**
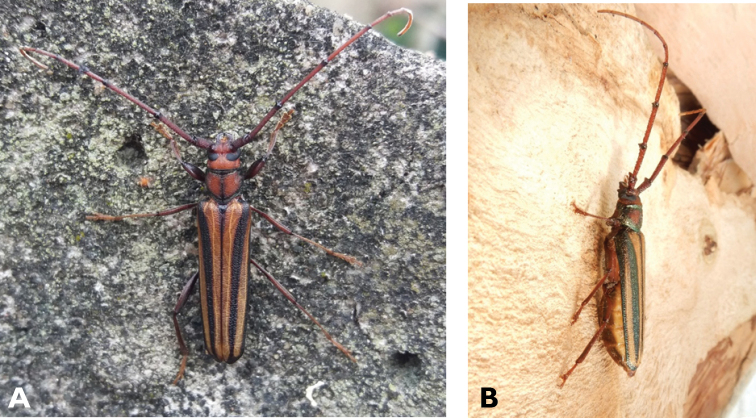
*Xystrocera globosa* (Olivier, 1795): dorsal (**A**) and lateral (**B**) views of specimens observed on Coloane Heights on 27 Mar 2020 and 11 Apr 2020, respectively (photographs: LC).

##### Subfamily Lamiinae Latreille, 1825

###### Tribe Acanthocinini Blanchard, 1845

####### Genus Rondibilis

Taxon classificationAnimaliaColeopteraCerambycidae

Thomson, 1857b: 306.

FB0DBF22-9295-5BE6-A64C-58C7F23DB034

######## Type species.

*Rondibilis bispinosa* Thomson, 1857.

####### Rondibilis undulata

Taxon classificationAnimaliaColeopteraCerambycidae

(Pic, 1922)

5A96D305-99D5-549E-BC1D-99050C3A79BA

Fig. 26

Erysamena [sic] undulata Pic, 1922: 14. TL: Vietnam (Tonkin); TD: MNHN.
Rondibilis multinotatus Gressitt, 1939: 83. TL: China (Guangdong); TD: SYSU.

######## Distribution.

Palaearctic Region: China (Guangdong, Hainan); South Korea (Lin and Yang 2019; Danilevsky 2020). Oriental Region: Vietnam (Lin and Yang 2019).

######## Macau records.

Great Taipa, 26 Apr 2019, in mosquito trap, R Perissinotto & L Clennell (IZCAS × 2); ibidem 9 May 2019, on dead tree branch, R Perissinotto (IZCAS); Coloane Village, 26 May 2020, under light in ablution block, R Perissinotto & L Clennell; ibidem 1 Jun 2020, R Perissinotto (MACT); St. Francis Xavier’s Parish [Coloane], 25 May 2020 12:10, Kit Chang (https://www.inaturalist.org/observations/47149980); ibidem 24 May 2020 19:33, Kisu Wong (https://www.inaturalist.org/observations/54388818); Taipa Grande, 30 Apr 2021, Lynette Clennell (https://www.inaturalist.org/observations/76032776).

######## Remarks.

In Macau, adults are active only in late spring and range in total length 6.5–8 mm and 1.5–2 mm in maximum width. They are strictly nocturnal and readily attracted to artificial lights. No information is available in the literature on their larval host plant(s).

**Figure 26.**
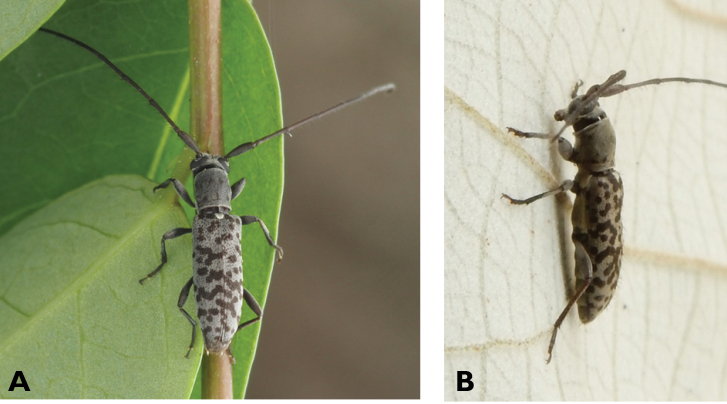
*Rondibilis undulata* (Pic, 1922): dorsal (**A**) and lateral (**B**) views of specimens observed on Coloane Heights (25 May 2020) and Coloane Village (26 May 2020), respectively (photographs: **A** Kit Chang **B** LC).

###### Tribe Apomecynini J. Thomson, 1860

####### Genus Apomecyna

Taxon classificationAnimaliaColeopteraCerambycidae

Dejean, 1821: 108.

41B71931-CCA6-5F53-A69B-7D9133D34BDA

######## Type species.

*Saperda alboguttata* Megerle, 1802 (= *Lamia histrio* Fabricius, 1793).

####### Apomecyna longicollis longicollis

Taxon classificationAnimaliaColeopteraCerambycidae

Pic, 1926

0D529434-A07B-5218-B862-A3A3E23A4578

Fig. 27

Apomecyna longicollis Pic, 1926: 28. TL: Vietnam (Tonkin); TD: MNHN

######## Distribution.

Palaearctic Region: China (Guizhou, Hong Kong, Jiangxi, Yunnan) (Yiu 2009; Lin and Yang 2019; Danilevsky 2020). Oriental Region: Laos; Thailand; Vietnam (Hua 2002).

######## Macau records.

Great Taipa, 26 Apr 2019, in ablution block, R Perissinotto & L Clennell (IZCAS); Little Taipa, 28 Apr 2019, crushed in ablution block, R Perissinotto (IZCAS); ibidem 26 Apr 2019, under monument spotlight (IZCAS × 2); Coloane Village, 25 Apr 2020 under light in ablution block, R Perissinotto & L Clennell (MACT); ibidem 31 Aug 2020, R Perissinotto & L Clennell; Taipa, Minho Str., 23 May 2020 8:04, Eric Kwan (https://www.inaturalist.org/observations/46988120); St. Francis Xavier’s Parish [Coloane], 24 May 2020 1:50, Kit Chang (https://www.inaturalist.org/observations/47082158); ibidem 12 Jun 2020 2:13, Kit Chang (https://www.inaturalist.org/observations/49251842); ibidem 24 May 2020 22:19, Kisu Wong (https://www.inaturalist.org/observations/54388789); ibidem 6 May 2021, Lynette Clennell (https://www.inaturalist.org/observations/77832643); Our Lady of Carmel’s Parish [Great Taipa], 31 Aug 2020 16:10, Lynette Clennell (https://www.inaturalist.org/observations/58131197).

######## Remarks.

In Macau, adults are active throughout spring and summer, ranging in total length 7–10 mm and 1.5–3 mm in maximum width. Like the other species of this genus, *A. l. longicollis* is nocturnal and readily attracted to artificial lights. There is no published information on its larval host plant(s).

**Figure 27.**
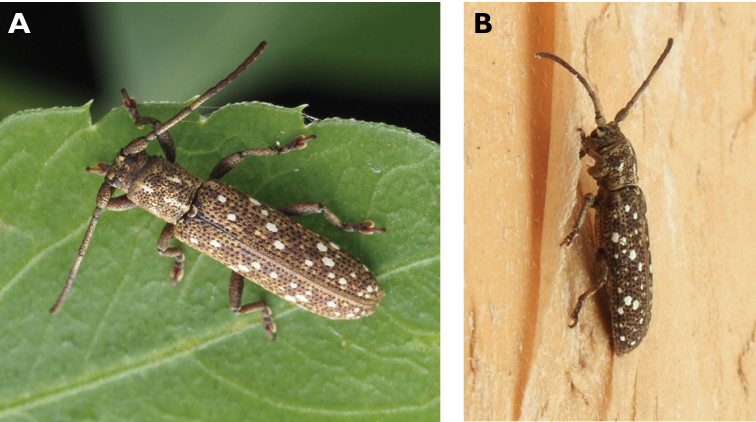
*Apomecyna longicollis longicollis* Pic, 1926: dorsal (**A**) and lateral (**B**) views of specimens observed on Coloane Heights (12 Jun 2020) and Coloane Village (25 Apr 2020), respectively (photographs: **A** Kit Chang **B** LC).

####### Apomecyna saltator

Taxon classificationAnimaliaColeopteraCerambycidae

(Fabricius, 1787)

982301B3-F0D8-55A6-ACA4-97E83FB665C2

Fig. 28

Lamia saltator Fabricius, 1787: 141. TL: Unknown; TD: ZMUC.

######## Distribution.

Palaearctic Region: China (Fujian, Guangdong, Guangxi, Guizhou, Hainan, Hong Kong, Hubei, Hunan, Jangsu, Jiangxi, Shaanxi, Sichuan, Taiwan, Yunnan, Zhejiang); India (Arunachal Pradesh, Himachal Pradesh); Pakistan; Nepal (Yiu 2009; Lin and Yang 2019; Danilevsky 2020). Oriental Region: India; Laos; Vietnam (Hua 2002; Kumawat et al. 2015).

######## Macau records.

Great Taipa, 29 Apr 2019, on mosquito trap, R Perissinotto & L Clennell (IZCAS); Coloane Village, 31 May 2020, under light in ablution block, R Perissinotto & Lynette Clennell; St. Francis Xavier’s Parish [Coloane], 9 Jun 2020 2:38, Kit Chang (https://www.inaturalist.org/observations/49012866); ibidem 12 Jun 2020 23:50, Kisu Wong (https://www.inaturalist.org/observations/55504513).

######## Remarks.

In Macau, adults have so far been recorded only in late spring and range in total length 10–12 mm and 3–4.5 mm in maximum width. In nearby Hong Kong, however, they have been observed throughout the summer and there larval food plants include *Cucurbita moschata*, *Benincasa hispida*, *Luffa acutangula* and *Lagenaria siceraria* (Yiu 2009). Elsewhere, larvae have also been found boring into stems of *Coccinia indica*, *Luffa aegyptiaca* and *Trichosanthes cucumerina* (Beeson 1941; Nair 1975; David and Ramamurthy 2012; Kumawat et al. 2015).

**Figure 28.**
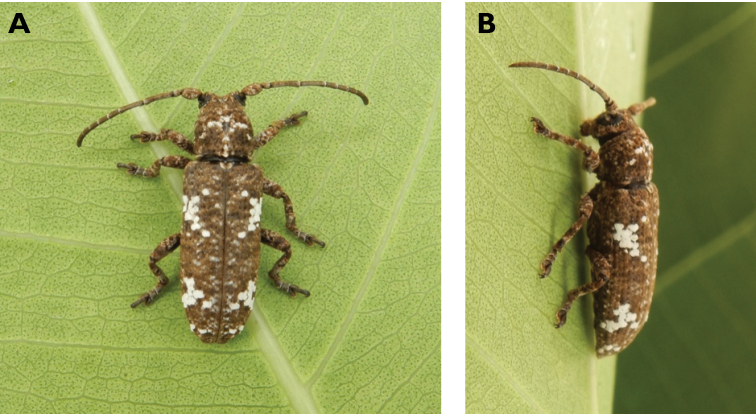
*Apomecyna saltator* (Fabricius, 1787): dorsal (**A**) and lateral (**B**) views of specimen observed at Coloane Village on 31 May 2020 (photographs: LC).

####### Genus Ropica

Taxon classificationAnimaliaColeopteraCerambycidae

Pascoe, 1858: 247.

C221091E-2E36-5724-A119-BA78130FF582

######## Type species.

*Ropica piperata* Pascoe, 1858.

####### Ropica dorsalis

Taxon classificationAnimaliaColeopteraCerambycidae

Schwarzer, 1925

B6C46FDC-A478-5822-BDB1-F8014FB8407A

Fig. 29

Ropica formosana var. dorsalis Schwarzer, 1925b: 145. TL: China (Taiwan); TD: SFNF

######## Distribution.

Palaearctic Region: China (Guangdong, Guangxi, Hainan, Hong Kong, Hunan, Jiangsu, Shanghai, Taiwan, Zhejiang); Japan, Nepal (Hayashi 1972, 1982; Lazarev and Murzin 2019; Lin and Yang 2019). Oriental Region: India; Laos; Vietnam (Lazarev 2019; Lin and Yang 2019).

######## Macau records.

1♀, Cotai Ecological Zone, 1^st^ zone, 14 Oct 2015, leg. Feng-Long Jia (SYSU); 1♂, ibidem 7 Apr 2018, leg. Wei-Cai Xie (SYSU); Great Taipa, 7 May 2019, on wall in ablution block, R Perissinotto & L Clennell (IZCAS); ibidem 12 May 2021, Lynette Clennell (https://www.inaturalist.org/observations/78523371); St. Francis Xavier’s Parish [Coloane], 30 Apr 2021, Lynette Clennell (https://www.inaturalist.org/observations/76108157).

######## Remarks.

Macau specimens exhibit a total length of 6.0–6.5 mm and a maximum width of 2.0–2.5 mm. The species is nocturnal and attracted to artificial lights. In the past, it has been misidentified and confused with *R. honesta* (Hua 2002; Chou 2004, 2008; Yiu 2009), with consequent mix up of their respective distribution records (Hua 2002; Lin and Yang 2019). According to Gressitt (1951), *Cucumis sativus* is among its host plants and in Hong Kong larvae have been found boring into stems of *Cucumis melo* (Yiu 2009).

**Figure 29.**
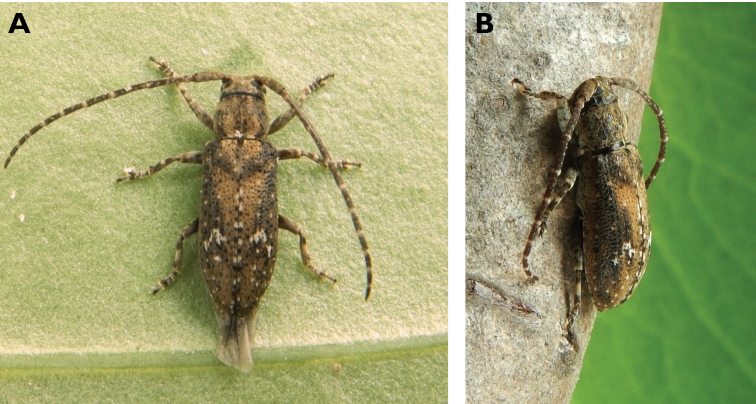
*Ropica dorsalis* Schwarzer, 1925: dorsal (**A**) and lateral (**B**) views of specimens observed on Great Taipa Hill on 7 May 2019 and at Coloane Village on 30 Apr 2021, respectively (photographs: LC).

####### Genus Sybra

Taxon classificationAnimaliaColeopteraCerambycidae

Pascoe, 1865: 141.

91D3A3E1-DD17-56A7-A10D-09415E83F3D7

######## Type species.

*Ropica stigmatica* Pascoe, 1859.

####### Sybra marmorea

Taxon classificationAnimaliaColeopteraCerambycidae

Breuning, 1939

A5BF2BE9-A7F8-5511-87C2-37783DB9B5CC

Fig. 30

Sybra marmorea Breuning, 1939: 264. TL: China; TD: NHMUK.

######## Distribution.

Palaearctic Region: China (Lin and Yang 2019; Danilevsky 2020). Oriental Region: Vietnam (Lin and Yang 2019).

######## Macau records.

Coloane Village, 20 May 2021, on building wall under street light, R Perissinotto & L Clennell (IZCAS; https://www.inaturalist.org/observations/79725419).

######## Remarks.

This is a highly significant record, as the type locality of this species was only vaguely reported as “China” in the original description of Breuning (1939), without reference to specific region or place. The only specimen observed in Macau so far exhibits a total length of 9 mm and a maximum width of 2.5 mm. The species is obviously nocturnal and attracted to artificial lights. Nothing appears to be known about the larval host plant(s) and general biology of this species.

**Figure 30.**
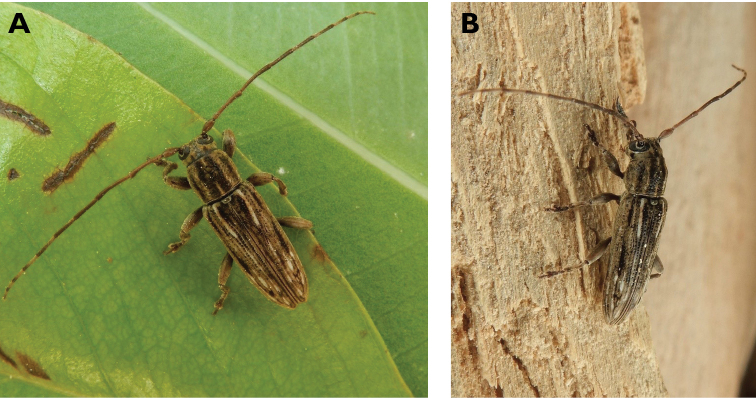
*Sybra marmorea* Breuning, 1939: dorsal (**A**) and lateral (**B**) views of specimen observed at Coloane Village on 20 May 2021 (photographs: LC).

####### Sybra posticalis

Taxon classificationAnimaliaColeopteraCerambycidae

(Pascoe, 1858)

3687B79F-9728-5604-AE72-F320C6FB933B

Fig. 31

Ropica posticalis Pascoe, 1858: 248. TL: China (Hong Kong); TD: NHMUK.

######## Distribution.

Palaearctic Region: China (Hainan, Hong Kong, Taiwan) (Yiu 2009; Lin and Yang 2019; Danilevsky 2020).

######## Macau records.

1♂, Coloane, 16 Apr 1994, PF Cheong (CIAM); 2♂, 2♀, Cotai Ecological Zone, 1^st^ zone, 4–5 Apr 2013, leg. Feng-Long Jia & Wei-Cai Xie (SYSU); Great Taipa, 22 Apr 2019, in mosquito trap, R Perissinotto & L Clennell (IZCAS × 2); ibidem 13 May 2019, on wall in ablution block, R Perissinotto & L Clennell (MACT): ibidem 13 Jun 2019, on floor in ablution block, R Perissinotto & L Clennell (IZCAS); Coloane Village, 22 Jun 2019, on wall in ablution block, R Perissinotto & L Clennell (IZCAS); St. Francis Xavier’s Parish [Coloane], 24 May 2020 2:14, Kit Chang (https://www.inaturalist.org/observations/47082176); Nossa Senhora do Carmo, Ilhas [Little Taipa], 9 May 2021 13:27, Kit Chang (https://www.inaturalist.org/observations/78034724).

######## Remarks.

In Macau, adults seem to be active only in late spring and range in total length 5.5–8 mm and 1.5–3 mm in maximum width. Activity is mainly nocturnal and individuals are readily attracted to artificial lights. In Hong Kong, a larva was reared successfully in captivity to adulthood when fed a mixture of soft dead woods (Yiu 2009).

**Figure 31.**
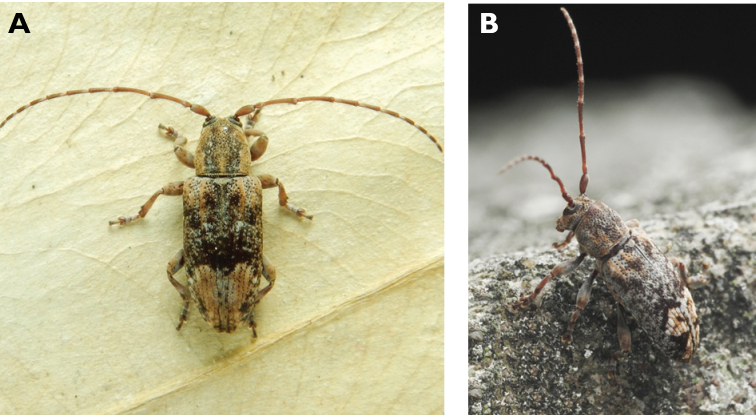
*Sybra posticalis* (Pascoe, 1858): dorsal (**A**) and lateral (**B**) views of specimens observed at Coloane Village (22 Jun 2019) and on Coloane Heights (24 May 2020), respectively (photographs: **A** LC **B** Kit Chang).

###### Tribe Batocerini J. Thomson, 1864

####### Genus Batocera

Taxon classificationAnimaliaColeopteraCerambycidae

Dejean, 1835: 341.

3767349D-1F49-50D1-A96F-90A9289D5904

######## Type species.

*Cerambyx rubus* Linnaeus, 1758.

####### Batocera rubus rubus

Taxon classificationAnimaliaColeopteraCerambycidae

(Linnaeus, 1758)

3565921E-7568-5C4C-87AD-C10D5FFE56DF

Fig. 32

Cerambyx rubus Linnaeus, 1758: 390. TL: India; TD: Unknown

######## Distribution.

Palaearctic Region: China (Fujian, Guangdong, Guangxi, Guizhou, Hainan, Hong Kong, Shaanxi, Shanxi, Sichuan, Taiwan, Yunnan, Zhejiang); India (Arunachal Pradesh, Uttarakhand); Japan (Ryukyus); Nepal; Pakistan; Saudi Arabia; Turkey (Yiu 2009; Lin and Yang 2019; Danilevsky 2020). Oriental Region: India; Indonesia (Lesser Sunda Islands, Borneo-Kalimantan, Sumatra); Laos; Malaysia (Malayan Peninsula, Sarawak, Sabah); Myanmar; Thailand, Vietnam (Kumawat et al. 2015).

**Figure 32.**
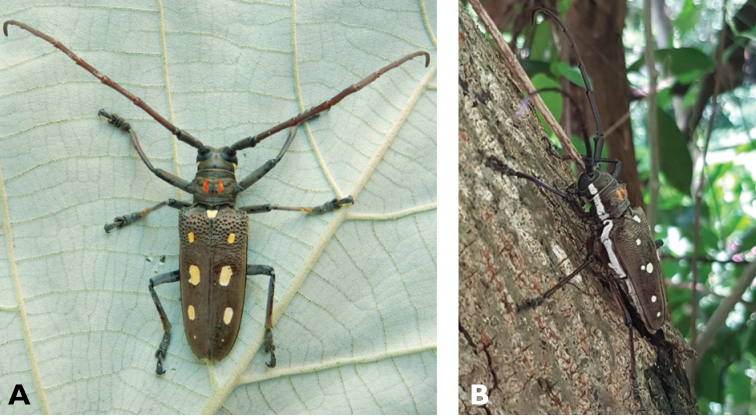
*Batocera rubus rubus* (Linnaeus, 1758): dorsal (**A**) and lateral (**B**) views of specimens observed on Little Taipa Hill (23 Apr 2019) and Coloane Village (2 Jul 2020), respectively (photographs: LC).

######## Macau records.

Taipa, University of East Asia Campus, 6 July 1991, on Block I (Easton 1991: 110; 1993: 46); Macau, University of East Asia, no data, ER Easton leg (UMEC); no data, “*Batocera rubus* (Linnaeus), 榕八星天牛32 mm ” (Pun and Batalha 1997: 64, fig. 97); 1♂, Coloane, 3 Sep 1993, F Macedo, *Batocera rubus* (CIAM); 1♂, ibidem 31 May 2000, CN Chan (CIAM); 1♀, ibidem 11 Oct 2002, KW Ho (CIAM); 1♀, Macau, 7 Sep 1997, SV Lam (CIAM); 1♂, No data, *Batocera rubus* (CIAM); Little Taipa, 23 Apr 2019, at monument spotlight, R Perissinotto & L Clennell (IZCAS); ibidem 26 Apr 2019, R Perissinotto; ibidem 13 May 2019, R Perissinotto & L Clennell (IZCAS); Great Taipa, 8 May 2019, under spotlight, R Perissinotto; Macau, 11 May 2019 (Daisy Li); Coloane Village, 24 Apr 2020, under spotlight outside prison building, R Perissinotto; ibidem 18 May 2020, on trunk of *Ficus rumphii*, R Perissinotto; ibidem 12 Jun 2020, R Perissinotto; ibidem 7 Jul 2020, R Perissinotto; Coloane, Cheoc Van, 6 Jul 2019, dead on ground, Lynette Clennell (MACT); Great Taipa, 28 Aug 2019, R Perissinotto & L Clennell (MACT); Guia Hill Municipal Park, 31 Jul 2017 22:41, Eric Kwan (https://www.inaturalist.org/observations/23090665); Macau, St Lazarus Parish, 5 Nov 2016 21:09, Kisu Wong (https://www.inaturalist.org/observations/23851435); Little Taipa Hill, 3 May 2019 10:11, Eric Kwan (https://www.inaturalist.org/observations/24446612); Taipa, Northeast Road, 23 May 2020 22:53, Eric Kwan (https://www.inaturalist.org/observations/47008047); Taipa, Qitan Highway, 28 May 2020 21:52, Eric Kwan (https://www.inaturalist.org/observations/47699538); St. Francis Xavier’s Parish [Coloane], 30 May 2020 1:21, Kit Chang (https://www.inaturalist.org/observations/47765481); ibidem 7 Jun 2020 20:58, Kisu Wong (https://www.inaturalist.org/observations/55385398); Coloane, Tin Hau Temple, 13 Jun 2020 00:35, Benny Kuok (https://www.inaturalist.org/observations/49416193); Coloane Heights Road, 18 Jun 2020 21:53, Kelvin Joshua Che (https://www.inaturalist.org/observations/50051088).

######## Remarks.

This is the largest longhorn beetle encountered in the Macau SAR during the current census, attaining a total length of 24–36 mm and a maximum width of 8–11 mm. Adults are active from late spring till mid-autumn, both during the day and night. The larvae burrow in a wide variety of forest trees, including *Artocarpus heterophyllus*, *Careya arborea*, *Ficus* spp. and *Mangifera* spp., from India through southeast Asia and south China, including Hong Kong (Easton 1991; Kumawat et al. 2015).

###### Tribe Desmiphorini Thomson, 1860

####### Genus Pseudoterinaea

Taxon classificationAnimaliaColeopteraCerambycidae

Breuning, 1940: 178.

49EBB8CC-94C6-5A79-9662-A0F942EAB596

######## Type species.

*Pseudanaesthetis bicoloripes* Pic, 1926.

####### Pseudoterinaea bicoloripes

Taxon classificationAnimaliaColeopteraCerambycidae

(Pic, 1926)

7802DD7B-1CF2-5831-9C0A-55FD97D62025

Fig. 33

Pseudanaesthetis bicoloripes Pic, 1926: 26. TL: Vietnam (Tonkin); TD: MNHN

######## Distribution.

Palaearctic Region: China (Fujian, Guangdong, Guangxi, Hainan, Hong Kong, Yunnan) (Yiu 2009; Lin and Yang 2019; Danilevsky 2020). Oriental Region: Laos; Vietnam (Hua 2002).

**Figure 33.**
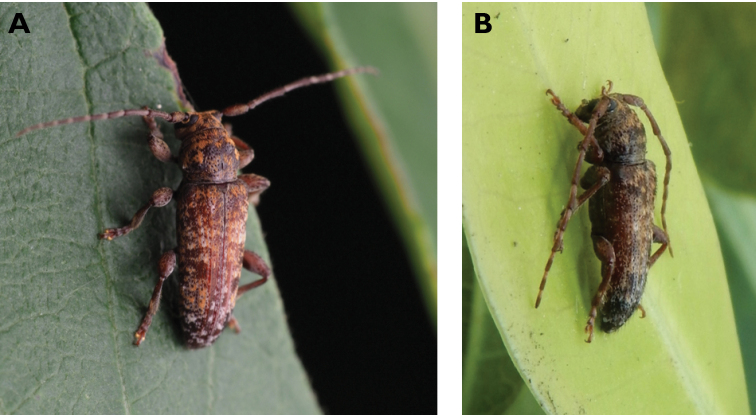
*Pseudoterinaea bicoloripes* (Pic, 1926): dorsal (**A**) and lateral (**B**) views of specimens observed on Little Taipa (3 May 2019) and Great Taipa (15 May 2019), respectively (photographs: **A** Kit Chang **B** LC).

######## Macau records.

1♀, Coloane, 9 May 1994, WW Pun (CIAM); Great Taipa, 9 Apr 2019, under light in ablution block, R Perissinotto & L Clennell; ibidem 28 Apr 2019, R Perissinotto & L Clennell (IZCAS); ibidem 15 May 2019, R Perissinotto & L Clennell (IZCAS); Macau, 27 May 2019, Kit Chang; Our Lady of Carmel’s Parish [Little Taipa], 3 May 2019 21:45, Eric Kwan (https://www.inaturalist.org/observations/24446701); ibidem 3 May 2019 21:46, Kit Chang (https://www.inaturalist.org/observations/24501567); ibidem 4 Apr 2020 12:30, Kit Chang (https://www.inaturalist.org/observations/43052257); ibidem 24 Apr 2020 12:37, Kisu Wong (https://www.inaturalist.org/observations/43313431); St. Francis Xavier’s Parish [Coloane], 24 May 2020 23:45, Kit Chang (https://www.inaturalist.org/observations/47149914); ibidem 24 May 2020 19:18, Kisu Wong (https://www.inaturalist.org/observations/54388784); Taipa, “Our Lady of Hope” Wetland, 18 Jun 2020 22:36, Eric Kwan (https://www.inaturalist.org/observations/50069191); St. Lazarus’ Parish [Guia Hill], 24 Jul 2020 22:55, Kit Chang (https://www.inaturalist.org/observations/54172350); Taipa Grande, 15 May 2021, Lynette Clennell (https://www.inaturalist.org/observations/78919645).

######## Remarks.

In Macau, adults are active throughout the spring and summer and range in total length 7–8.5 mm and 2–3 mm in maximum width. Activity appears to be mainly during night time, when specimens are readily attracted to artificial lights. No information is available in the literature on the larval food plant(s) of this species.

####### Genus Sophronica

Taxon classificationAnimaliaColeopteraCerambycidae

Blanchard, 1845: 160.

48C0D7DE-2F0B-56E3-91EB-61F295C8FBC3

######## Type species.

*Sophronica calceata* Chevrolat, 1855

####### Sophronica apicalis

Taxon classificationAnimaliaColeopteraCerambycidae

(Pic, 1922)

4F11B6C1-DA90-5D36-BDA6-BAA956B95CEC

Fig. 34

Phunginus apicalis Pic, 1922: 15. TL: Vietnam (Tonkin); TD: MNHN

######## Distribution.

Palaearctic Region: India (Uttarakhand); Nepal (Danilevsky 2020). Oriental Region: Thailand (https://www.thailandnatureproject.com/sophronica-apicalis.html).

######## Macau records.

Coloane Village, 1 May 2021, on wall under light in ablution block, R Perissinotto & L Clennell (IZCAS).

######## Remarks.

This is a new record for China. The specimen observed at Coloane exhibits a total length of 9 mm and a maximum width of 2.5 mm. Adult activity is presumably nocturnal and the specimen in question was obviously attracted to artificial light. No information is available in the literature on the larval food plant(s) or general biology of this species.

**Figure 34.**
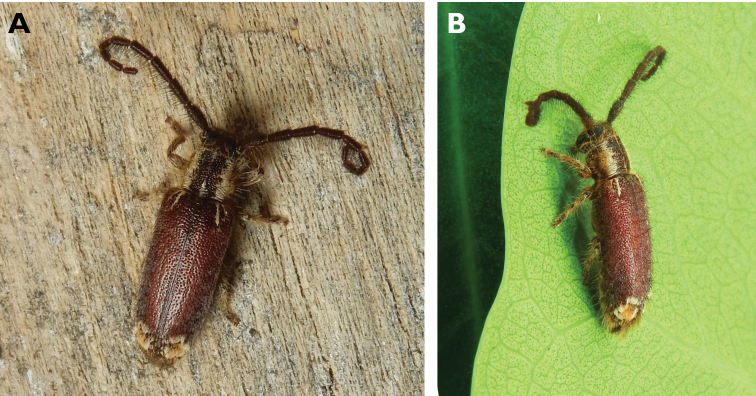
*Sophronica apicalis* (Pic, 1922): dorsal (**A**) and lateral (**B**) views of specimen observed at Coloane Village on 1 May 2021 (photographs: LC).

####### Genus Zotalemimon

Taxon classificationAnimaliaColeopteraCerambycidae

Pic, 1925: 29.

2D782CD7-5F08-5267-96E6-5F9CACFFE9B9

######## Type species.

*Zotalemimon apicale* Pic, 1925 (= *Sybra posticata* Gahan, 1895).

####### Zotalemimon ciliatum

Taxon classificationAnimaliaColeopteraCerambycidae

(Gressitt, 1942)

1AAEC85F-3643-5D9D-99C0-07E64E1C1DB0

Fig. 35

Donysia ciliata Gressitt, 1942: 212. TL: China (Guangdong, Honan Island); TD: SYSU.

######## Distribution.

Palaearctic Region: China (Fujian, Guangdong, Hainan, Hong Kong, Yunnan) (Yiu 2009; Lin and Yang 2019; Danilevsky 2020).

######## Macau records.

Great Taipa, 16 Mar 2019, under light in ablution block, R Perissinotto; Coloane Village, 3 Apr 2020, on the wall in ablution block, R Perissinotto.

######## Remarks.

In Macau, adults appear to be active only in the spring and range in total length 10.5–12 mm and 3–4 mm in maximum width. Adults are nocturnal and attracted to artificial lights. Known larval host plants include *Dendrocalamus latiflorus* and *Xylosma* sp. (Hua 2002; Yiu 2009).

**Figure 35.**
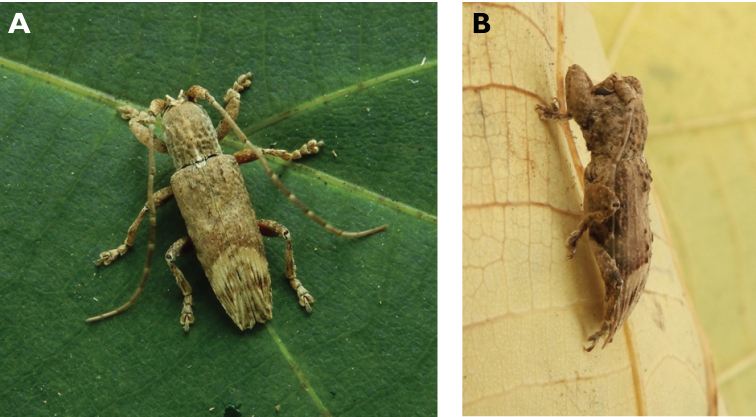
*Zotalemimon ciliatum* (Gressitt, 1942): dorsal (**A**) and lateral (**B**) views of specimens observed on Little Taipa (3 May 2019) and Great Taipa (15 May 2019), respectively (photographs: LC).

###### Tribe Dorcaschematini J. Thomson, 1860

####### Genus Olenecamptus

Taxon classificationAnimaliaColeopteraCerambycidae

Chevrolat, 1835: 134.

F151B911-9E08-5275-80AA-DB48478C8204

######## Type species.

*Olenecamptus serratus* Chevrolat, 1835 (= *Saperda biloba* Fabricius, 1801).

####### Olenecamptus taiwanus

Taxon classificationAnimaliaColeopteraCerambycidae

L. S. Dillon & E. S. Dillon, 1948

B4C9E68C-4098-542A-8E73-5C7739E9553D

Fig. 36

Olenecamptus bilobus taiwanus L. S. Dillon & E. S. Dillon, 1948: 229, pl. X, fig. 9. TL: China (Taiwan); TD: AMNH

######## Distribution.

Palaearctic Region: China (Guangdong, Guangxi, Hainan, Hong Kong, Taiwan, Yunnan); Japan (Yiu 2009; Lin and Yang 2019; Danilevsky 2020).

######## Macau records.

Taipa, University of East Asia Campus, 10 Sep 1991, on Block I, “*Olenecamptus bilobus*” (Easton 1991: 110); Macau, University of East Asia, 23 Apr 1990, ER Easton leg (UMEC); no data “*Olenecamptus bilobus tonkinus* Dillon et Dillon, 南方粉天牛15 mm” (Pun and Batalha 1997: 65, fig. 103); 1♂, Coloane, 28 Jun 1988, WW Pun, *Olenecamptus bilobus tonkinus* (CIAM); 1♀, ibidem 2 May 1994, PF Cheong, *Olenecamptus bilobus tonkinus* WW Pun det. (CIAM); Little Taipa, 23 Apr 2019, at monument spotlight, R Perissinotto & L Clennell (IZCAS); ibidem 19 Sep 2019, dead on trunk of *Ficus microcarpa*, R Perissinotto (MACT); Coloane Village, 26 May 2020, in mosquito electric trap, R Perissinotto & L Clennell (MACT); ibidem 26 May 2020, on branch of *Ficus rumphii*, R Perissinotto; ibidem 2 May 2021, Lynette Clennell (https://www.inaturalist.org/observations/76790090).

######## Remarks.

In Macau, adults are active from late spring to late summer and range in total length 11.5–20 mm and 2.5–5 mm in maximum width. According to Yiu (2009), in Hong Kong the larvae of this species complete their development inside dead branches of *Ficus* spp. trees. In Macau, adults were repeatedly found on the trunk of large *F. rumphii* and *F. microcarpa* trees on Little Taipa Hill (RP pers. obs.). Known host plants for the species include *Artocarpus* sp., *Bauhinia* sp., *Ficus infectoria*, *Hevea brasiliensis*, *Mangifera indica*, *Mangifera* sp., *Morus alba* and *Morus* sp. (Lin and Yang 2019).

**Figure 36.**
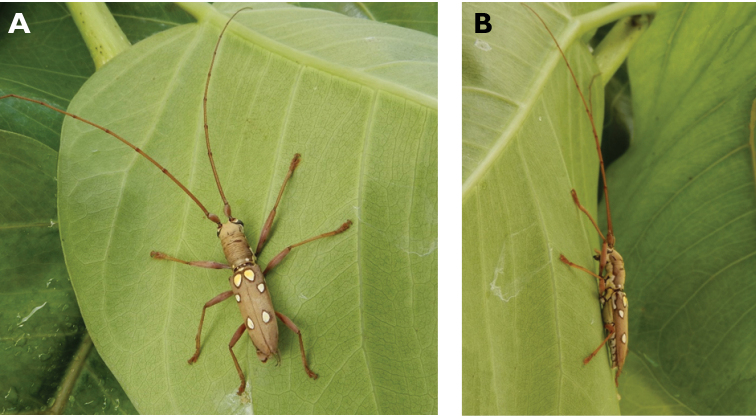
*Olenecamptus taiwanus* L. S. Dillon & E. S. Dillon, 1948: dorsal (**A**) and lateral (**B**) views of specimen observed at Coloane Village on 26 May 2020 (photographs: LC).

###### Tribe Exocentrini Pascoe, 1864

####### Genus Exocentrus

Taxon classificationAnimaliaColeopteraCerambycidae

Dejean, 1835: 339.

43676FBB-F522-5A95-A534-06FDEBB8156B

######## Type species.

*Cerambyx balteatus* Fabricius sensu Dejean 1835 (= *Cerambyx lusitanus* Linnaeus, 1767).

####### Exocentrus alboguttatus subconjunctus

Taxon classificationAnimaliaColeopteraCerambycidae

Gressitt, 1940

5B5B00BC-7E27-5F96-8BBC-2FF6D32F274D

Fig. 37

Exocentrus alboguttatus subconjunctus Gressitt, 1940a: 184. TL: China (Hainan); TD: SYSU

######## Distribution.

Palaearctic Region: China (Guangxi, Hainan, Hong Kong) (Yiu 2009; Lin and Yang 2019; Danilevsky 2020).

######## Macau records.

Great Taipa, 29 Apr 2019, at light in ablution block, R Perissinotto & L Clennell (IZCAS); ibidem 9 May 2019, R Perissinotto & L Clennell (IZCAS); ibidem 15 May 2019 on dead tree branch, R Perissinotto & L Clennell (IZCAS); ibidem 13 Jun 2019, on mosquito trap, R Perissinotto & L Clennell (IZCAS); Little Taipa, 26 Apr 2019, at monument spotlight, R Perissinotto & L Clennell (IZCAS); Coloane Village, 2 Jun 2019, on floor in ablution block (MACT); ibidem 13 May 2020, R Perissinotto & L Clennell (MACT, × 2); ibidem 8 Jun 2020, R Perissinotto & L Clennell (IZCAS); St. Francis Xavier’s Parish [Coloane], 24 May 2020 2:08, Kit Chang (https://www.inaturalist.org/observations/47082171); ibidem 24 May 2020 19:32, Kisu Wong, (https://www.inaturalist.org/observations/54388816); ibidem 30 Apr 2021, Lynette Clennell (https://www.inaturalist.org/observations/76032774); Taipa, Evora Str., 6 Jun 2020 1:38, Eric Kwan (https://www.inaturalist.org/observations/48558660); Our Lady of Carmel’s Parish [Great Taipa], 28 May 2020 00:37, Kit Chang (https://www.inaturalist.org/observations/47611059).

######## Remarks.

In Macau, adults are active from late spring till early summer and range in total length 4.5–7.5 mm and 1.5–3 mm in maximum width. They are readily attracted to artificial lights at night, but are also active during daytime when they can be observed crawling and mating on dead tree twigs and branches. In nearby Hong Kong, where in the past this species has been erroneously reported as *E. guttulatus subconjunctus*, the larval host plants include *Acacia farnesiana*, *Mallotus* spp. and *Morus alba* (Yiu 2009; Yiu and Yip 2011).

**Figure 37.**
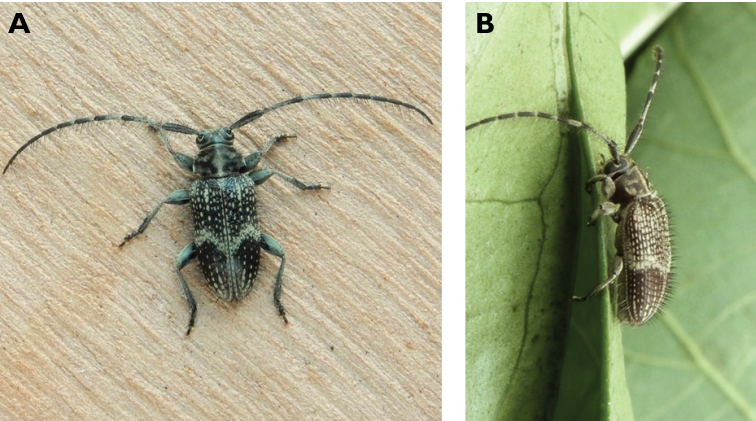
*Exocentrus alboguttatus subconjunctus* Gressitt, 1940: dorsal (**A**) and lateral (**B**) views of specimens observed on Great Taipa Hill (3 Jun 2018) and Coloane Village (25 May 2020), respectively (photographs: LC).

####### Exocentrus formosofasciolatus

Taxon classificationAnimaliaColeopteraCerambycidae

Kusama & Tahira, 1978

1B47575A-2B6D-5535-B579-9C728BF799AA

Fig. 38

Exocentrus (Camptomyne) formosofasciolatus Kusama & Tahira, 1978: 17, figs 7, 7 p. TL: China (Taiwan); TD: NSMT.

######## Distribution.

Palaearctic Region: China (Taiwan) (Lin and Yang 2019; Danilevsky 2020).

######## Macau records.

Great Taipa, 13 May 2019, in mosquito trap, R Perissinotto & L Clennell (MACT × 2); ibidem 13 Jun 2019, R Perissinotto & L Clennell (IZCAS); Taipa Village, 15 May 2019 on dead tree branch, R Perissinotto & L Clennell (IZCAS); Little Taipa, 13 May 2019, at monument spotlight, R Perissinotto & L Clennell (IZCAS).

######## Remarks.

In Macau, adults seem to be active only in late spring and range in total length 4–5 mm and 1.5–2 mm in maximum width. Like in its congeneric species above, individuals are active both during the daytime and at night, when they are readily attracted to artificial lights. No information is available on its larval food plant(s).

**Figure 38.**
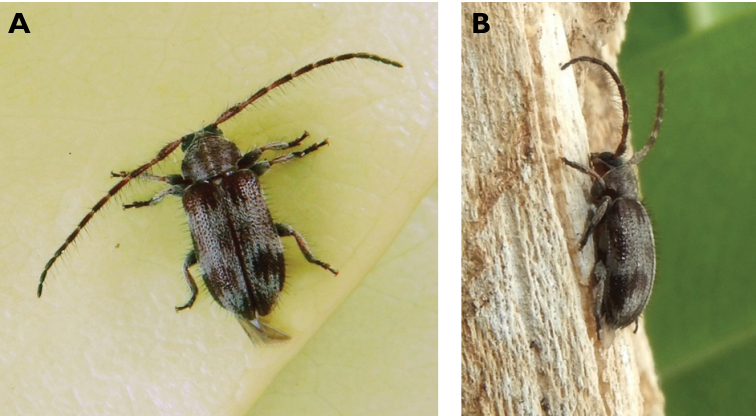
*Exocentrus formosofasciolatus* Kusama & Tahira, 1978: dorsal (**A**) and lateral (**B**) views of specimen observed on Great Taipa Hill on 13 May 2019 (photographs: LC).

###### Tribe Homonoeini J. Thomson, 1864

####### Genus Bumetopia

Taxon classificationAnimaliaColeopteraCerambycidae

Pascoe, 1858: 252.

1F3E98F6-DB09-55A5-A133-FD26B9E5589F

######## Type species.

*Bumetopia oscitans* Pascoe, 1858.

####### Bumetopia oscitans

Taxon classificationAnimaliaColeopteraCerambycidae

Pascoe, 1858

6E8F73F2-E3F2-526C-A0FC-71D60709E3A6

Fig. 39

Bumetopia oscitans Pascoe, 1858: 252. TL: China (Hong Kong); TD: NHMUK

######## Distribution.

Palaearctic Region: China (Hong Kong, Shaanxi, Taiwan); South Korea (Lin and Yang 2019; Danilevsky 2020).

######## Macau records.

1♂, Little Taipa, 25 Mar 2021, on branch of *Cinnamomum burmannii*, R Perissinotto (IZCAS).

######## Remarks.

The only specimen observed in Macau during this study exhibits a total length of 13 mm and a maximum width of 4 mm. In Hong Kong, adults have been reported feeding on *Miscanthus* sp. (Yiu 2009).

**Figure 39.**
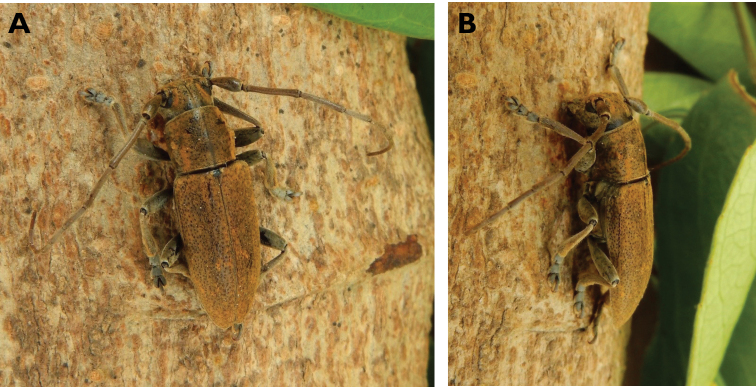
*Bumetopia oscitans* Pascoe, 1858: dorsal (**A**) and lateral (**B**) views of specimen observed on Little Taipa Hill on 25 Mar 2021 (photographs: LC).

###### Tribe Lamiini Latreille, 1825

####### Genus Anoplophora

Taxon classificationAnimaliaColeopteraCerambycidae

Hope, 1839: 43.

7F5E4FCB-F6F9-5332-A5A8-B2FBCF834DA9

######## Type species.

*Anoplophora stanleyana* Hope, 1839.

####### Anoplophora chinensis chinensis

Taxon classificationAnimaliaColeopteraCerambycidae

(Forster, 1771)

6808841F-317C-58E4-82D0-7192E9208A10

Fig. 40

Cerambyx chinensis Forster, 1771: 39. TL: China; TD: LSLU

######## Distribution.

Palaearctic Region: Austria (introduced); China (Anhui, Beijing, Fujian, Gansu, Guangdong, Guangxi, Guizhou, Hainan, Hebei, Henan, Hong Kong, Hubei, Hunan, Jangsu, Jiangxi, Jilin, Liaoning, Shaanxi, Shandong, Shanghai, Sichuan, Taiwan, Yunnan, Zhejiang); Croatia (introduced); France (introduced); Germany (introduced); Italy (introduced); Netherlands (introduced); Turkey (introduced); South Korea (Yiu 2009; Lin and Yang 2019; Danilevsky 2020). Oriental Region: Indonesia; Malaysia; Myanmar; Philippines; Vietnam (Lingafelter and Hoebeke 2002).

######## Macau records.

Taipa, University of East Asia Campus, 22 May 1991, near tennis courts under street lamp (Easton 1991: 110; 1993: 49); Macau, University of East Asia, no data, ER Easton leg (UMEC x3); no data, “*Anoplophora chinensis* (Forster), 星天牛34 mm ” (Pun and Batalha 1997: 64, fig. 94); 1♂, Coloane, 16 Jun 1992, Tai Ip, *Anoplophora chinensis* (CIAM); 1♀, ibidem 25 May 1995, Tai Ip, *Anoplophora chinensis* (CIAM); 1♂, ibidem 19 May 1994, *Melia azedarach*, Tai Ip, *Anoplophora chinensis* WW Pun det. (CIAM); 1♀, Taipa, 14 Apr 1993, *Casuarina equisetifolia*, WM Ng, *Anoplophora chinensis* (CIAM); Taipa Village, 23 Apr 2018, on trunk of *Leucaena leucocephala*, R Perissinotto & L Clennell; Taipa Central, 2 May 2019, dead on floor, R Perissinotto & L Clennell; Coloane Village, 20 Apr 2020, fresh elytron on road, R Perissinotto & L Clennell (MACT); ibidem 26 Apr 2020, female on tree trunk, R Perissinotto & L Clennell (MACT); ibidem 15 Jun 2019, on trunk of *Mallotus paniculatus*, R Perissinotto & L Clennell (MACT); Taipa, Museum Houses, 10 May 2020 14:05, Kisu Wong (https://www.inaturalist.org/observations/52588617); Coloane Village, 30 Apr 2021, Lynette Clennell (https://www.inaturalist.org/observations/76032800); ibidem 1 May 2021 13:02, Lynette Clennell (https://www.inaturalist.org/observations/76100043); Macau University of Science & Technology, 1 May 2021 10:43, Amanda Wan (https://www.inaturalist.org/observations/76454987).

######## Remarks.

Easton (1993) reported that this species was common in Macau during the period 1991–1993. Despite having a very wide distribution range and being regarded as a pest and invasive species in some countries, it is now a rather scarce occurrence in Macau, where adults are active only from late spring to early summer. It ranges in total length 24–35 mm and 10.5–13 mm in maximum width. This species is often referred to as “the citrus longhorn beetle” (Easton 1991, 1993) and its larvae are considered a serious pest of citrus in Hong Kong and adjacent mainland China (Hill et al. 1982). In Macau, where citrus trees are very scarce, it has been suggested that larvae may complete their growth in wood of *Melia azedarach* (Easton 1993). The larvae of this species are actually extreme opportunists in their diet and consume a large variety of woody plants, including horticultural species, and adults are therefore often encountered in city gardens and farms (Yiu 2009). Among the best known host plants are *Acer saccharinum*, *Alnus firma*, A. hirsuta f. glabra, *Atalantia buxifolia*, Betula platyphylla var. japonica, *Broussonetia papyrifera*, *Castanea* sp., *C. crenata*, *Citrus* sp., *C. junos*, *C. unshiu*, *Cryptomeria japonica*, *Ficus carica*, *Hibiscus* sp., *H. syriacus*, *Juglans* sp., *J. mandshurica*, *Lagerstroemia indica*, *Mallotus japonicus*, *Malus pumila*, *Melia azedarach*, *Momordica charantia*, *Morus* sp., *M. alba*, *Platanus occidentalis*, *P. orientalis*, *Poncirus trifoliata*, *Populus* spp., *Prunus* spp., *Psidium guajava*, *Punica granatum*, Pyrus pyrifolia var. culta, *P. ussuriensis*, *Rosa* sp., *R. multiflora*, *R. rugosa*, *Salix* sp., *S. babylonica*, *S. koreensis*, *Styrax japonicas* and Ulmus davidiana var. japonica (Lim et al. 2014; Lin and Yang 2019).

**Figure 40.**
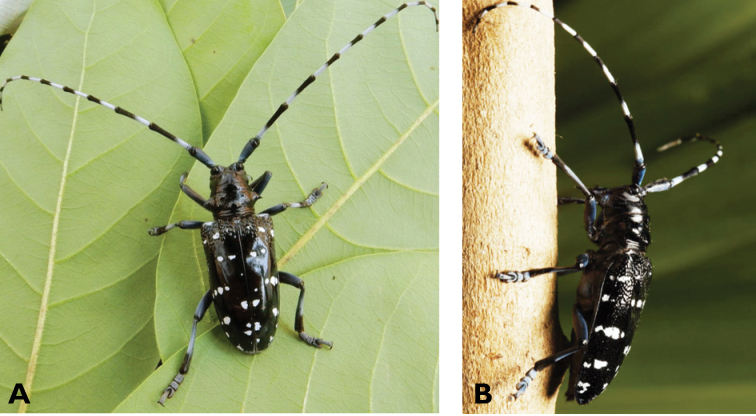
*Anoplophora chinensis chinensis* (Forster, 1771): dorsal (**A**) and lateral (**B**) views of specimens observed at Coloane Village on 26 Apr 2020 and Taipa Village on 10 May 2020, respectively (photographs: **A** LC **B** Kisu Wong).

####### Genus Blepephaeus

Taxon classificationAnimaliaColeopteraCerambycidae

Pascoe, 1866: 249.

C710A058-F357-555C-BC72-FF150A1F8D73

######## Type species.

*Monohammus succintor* Chevrolat, 1852.

####### Blepephaeus subcruciatus

Taxon classificationAnimaliaColeopteraCerambycidae

(White, 1858)

610BE247-D58D-5AE1-B652-FDF74074FA9A

Fig. 41

Monohammus subcruciatus White, 1858: 410. TL: China (Hong Kong); TD: NHMUK

######## Distribution.

Palaearctic Region: China (Guangdong, Guangxi, Hainan, Hong Kong) (Yiu 2009; Lin and Yang 2019; Danilevsky 2020).

######## Macau records.

Guia Hill, 12 May 2019, near light in ablution block, R Perissinotto & L Clennell (IZCAS).

######## Remarks.

Only one specimen was observed in Macau during the entire study period, exhibiting a total length of 20 mm and a maximum width of 6 mm. Adults seem to be active in late spring and mainly at night, being attracted to artificial lights. There is no published information on its biology.

**Figure 41.**
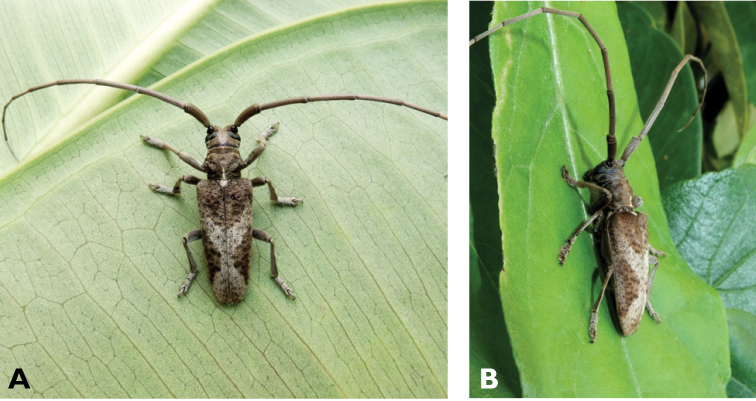
*Blepephaeus subcruciatus* (White, 1858): dorsal (**A**) and lateral (**B**) views of specimen observed on Guia Hill on 12 May 2019 (photographs: LC).

####### Blepephaeus succinctor

Taxon classificationAnimaliaColeopteraCerambycidae

(Chevrolat, 1852)

BA5312AD-C77E-5F6C-AA06-DB95A3DB27DF

Fig. 42

Monohammus succinctor Chevrolat, 1852: 417. TL: China (Shanghai); TD: NHMUK

######## Distribution.

Palaearctic Region: China (Guangdong, Guangxi, Hainan, Hunan, Hong Kong, Jiangsu, Jiangxi, Shaanxi, Shanghai, Sichuan, Taiwan, Xizang, Yunnan, Zhejiang); India (Arunachal Pradesh, Sikkim); Nepal (Yiu 2009; Lin and Yang 2019; Danilevsky 2020). Oriental Region: Bangladesh; India; Laos, Malaysia; Thailand, Vietnam (Mitra et al. 2017).

######## Macau records.

Guia Hill, 4 May 2019, crushed on pavement, R Perissinotto & L Clennell (IZCAS); Coloane Heights, under statue spotlight, 11 May 2019, R. Perissinotto & L Clennell (IZCAS); ibidem 31 May 2020, R Perissinotto & L Clennell; Great Taipa, 4 Jun 2019, at light in ablution block, R Perissinotto & L Clennell; St. Francis Xavier’s Parish [Coloane], 24 May 2020 2:22, Kit Chang (https://www.inaturalist.org/observations/47082181); ibidem 7 May 2021, Lynette Clennell (https://www.inaturalist.org/observations/77838218).

######## Remarks.

In Macau, adults are active only in late spring and range in total length 22–27 mm and 6–9 mm in maximum width. They are mainly nocturnal and readily attracted to artificial lights. In nearby Hong Kong, larvae utilise a wide variety of food plants, including *Adenanthera miscrosperma*, *Citrus reticulata*, *Melia azedarach*, *Morus alba*, and *Vernicia fordii* (Yiu 2009). Other host plants include *Acacia confusa*, *Adenanthera pavonina*, *Albizia* sp., *Bambusa* sp., *Casuarina equisetifolia*, *Cinnamomum camphora*, *Citrus* sp., *Cunninghamia lanceolata*, *Firmiana simplex*, *Juglans regia*, *Olea europaea*, *Paulownia* sp., *Quercus* sp. and *Styphnolobium japonicum* (Lin and Yang 2019).

**Figure 42.**
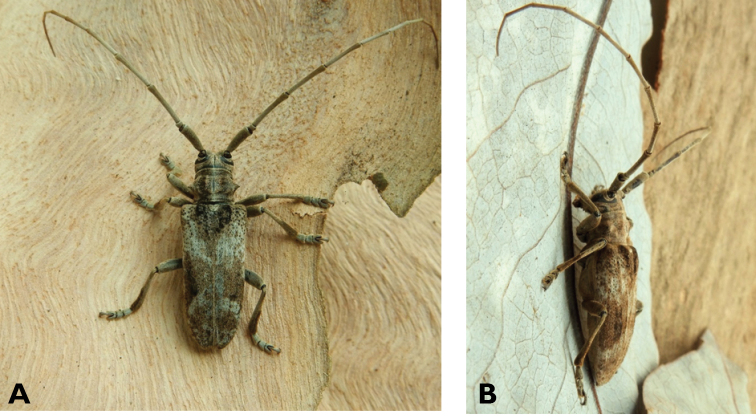
*Blepephaeus succinctor* (Chevrolat, 1852): dorsal (**A**) and lateral (**B**) views of specimens observed on Great Taipa Hill (4 Jun 2019) and Coloane Heights (31 May 2020), respectively (photographs: LC).

####### Genus Eutaenia

Taxon classificationAnimaliaColeopteraCerambycidae

J. Thomson, 1857a: 184.

9AF95199-BC9C-5211-909E-C6D8A3EC3EF5

######## Type species.

*Ceroplesis javeti* J. Thomson, 1857 (= *Lamia trifasciella* White, 1850).

####### Eutaenia tanoni

Taxon classificationAnimaliaColeopteraCerambycidae

Breuning, 1962

3DA55130-0173-58BC-9F67-C8FE060A9493

Fig. 43

Eutaenia tanoni Breuning 1962a:18. TL: Laos; TD: BPBM

######## Distribution.

Palaearctic Region: China (Guangxi) (Huang et al. 2002). Oriental Region: Laos (Breuning 1962a; Rondon and Breuning 1970).

######## Macau records.

Coloane, Cheoc Van, on coastal vegetation, 18 May 2019, R Perissinotto & L Clennell (IZCAS); ibidem 19 May 2020, R Perissinotto & L Clennell; ibidem 22 May 2020, R Perissinotto & L Clennell (MACT); Coloane, Aldeia Road, 16 May 2020 17:55, Annie Lao (https://www.inaturalist.org/observations/46079399).

######## Remarks.

Since this species was originally described on the basis of a single specimen, Rondon and Breuning (1970) suggested that it may have represented a natural hybrid between the more common and widely distributed *Eutaenia trifasciella* (White, 1850) and *E. corbetti* Gahan, 1893. It differs from the closely related *E. trifasciella* from Hong Kong mainly by having the apical four antennomeres with basal parts lightly testaceous rather than completely black and the black marking on pronotum extending to both anterior and posterior margins, instead of forming only a middle transverse black stripe. In Macau, adults are active only in late spring and range in total length 20.5–23 mm and 6–7 mm in maximum width. They are strictly diurnal and feed on the bark of coastal shrubs (RP & LC pers. obs.).

**Figure 43.**
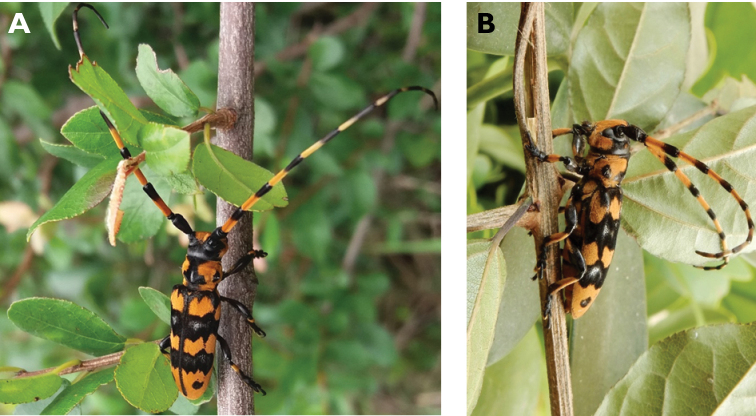
*Eutaenia tanoni* Breuning 1962: dorsal (**A**) and lateral (**B**) views of specimens observed along the Coloane coast on 19 May 2020 (photographs: LC).

####### Genus Monochamus

Taxon classificationAnimaliaColeopteraCerambycidae

Dejean, 1821: 106.

D4EA7F3F-E937-56E4-8B20-A69B76E21D1E

######## Type species.

*Cerambyx sutor* Linnaeus, 1758.

####### Monochamus alternatus alternatus

Taxon classificationAnimaliaColeopteraCerambycidae

Hope, 1842

4973F24E-EF7B-584A-A731-F6828C7EE01B

Fig. 44

Monohammus alternatus Hope, 1842: 61. TL: China (Zhejiang); TD: OXUM
Monohammus tesserula White, 1858: 408. TL: China (Hong Kong): TD: NHMUK

######## Distribution.

Palaearctic Region: China (Anhui, Beijing, Fujian, Guangdong, Guangxi, Guizhou, Hebei, Henan, Hong Kong, Hubei, Hunan, Jiangsu, Jiangxi, Shaanxi, Shandong, Sichuan, Taiwan, Xizang, Yunnan, Zhejiang); South Korea (Yiu 2009; Lin and Yang 2019; Danilevsky 2020). Oriental Region: Laos; Vietnam (Akbulut et al. 2017).

######## Macau records.

1♀, Coloane, 19 Apr 2001, CM Chan, *Monochamus alternatus* Hope ♀ (CIAM); 1♂, ibidem 25 Apr 2001, CM Chan, *Monochamus alternatus* Hope ♂ (CIAM); 1♂, ibidem 26 Apr 2001, CM Chan, *Monochamus alternatus* Hope ♂ (CIAM); Coloane Heights, A-Mà statue, 22 May 2020, R Perissinotto; ibidem 30 May 2020, dead under spot-light, R Perissinotto & L Clennell (IZCAS); St. Francis Xavier’s Parish [Coloane], 24 May 2020 22:52, Kit Chang (https://www.inaturalist.org/observations/47149824); ibidem 24 May 2020 9:13, Kisu Wong (https://www.inaturalist.org/observations/542858480).

######## Remarks.

In Macau, adults are active mainly at night and only in late spring; they range in total length 18–21 mm and 6–7.5 mm in maximum width. In Hong Kong, larvae reportedly bore into *Pinus massoniana* and carry the pine-wood nematode *Bursaphelenchus xilophilus*, which is a pest of pine plantations (Yiu 2009). Other larval food plants include *Abies firma*, *A. holophylla*, *Cedrus deodara*, *C. libani*, *Cryptomeria japonica*, *Juniperus* sp., *J. chinensis*, *Larix* sp., *Larix gmelinii*, *Malus asiatica*, *M. pumila*, *Morinda umbellata*, *Picea* sp., *P. excelsa*, *P. morinda*, *Pinus armandii*, *P. banksiana*, *P. densiflora*, *P. elliottii*, *P. khasya*, *P. koraiensis*, *P. luchuensis*, *P. massoniana*, *P. rigida*, *P. strobus*, *P. taeda*, *P. thunbergii*, *P. yunnanensis* and *Quercus* sp. (Lim et al. 2014; Lin and Yang 2019).

**Figure 44.**
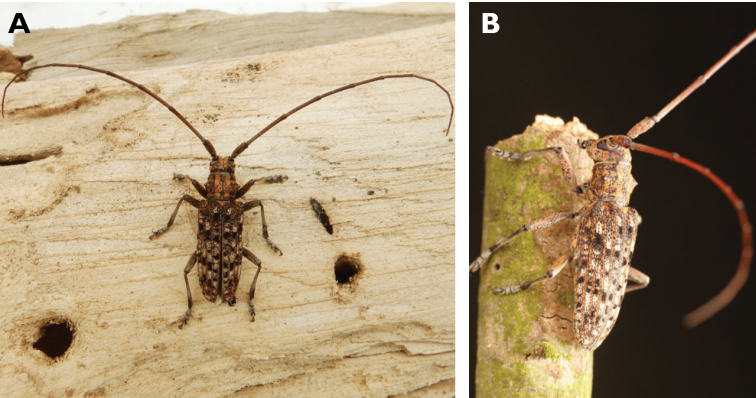
*Monochamus alternatus alternatus* Hope, 1842: dorsal (**A**) and lateral (**B**) views of specimens observed on the Coloane Heights on 22 May 2020 and on 24 May 2020, respectively (photographs: **A** LC **B** Kisu Wong).

###### Tribe Mesosini Mulsant, 1839

####### Genus Coptops

Taxon classificationAnimaliaColeopteraCerambycidae

Audinet-Serville, 1835: 64.

94B652FA-079B-5156-AD81-07E148A92C53

######## Type species.

*Coptops parallela* Audinet-Serville, 1835 (= *Lamia aedificator* Fabricius, 1793).

####### Coptops licheneus

Taxon classificationAnimaliaColeopteraCerambycidae

Pascoe, 1865

18E306D1-BD2F-5452-87FA-49A4906031DE

Fig. 45

Coptops lichenea Pascoe, 1865: 118. TL: Malaysia (Malacca); TD: NHMUK

######## Distribution.

Palaearctic Region: China (Fujian, Guangdong, Guangxi, Hainan, Hong Kong, Yunnan); Nepal (Yiu 2009; Lin and Yang 2019; Danilevsky 2020). Oriental Region: Laos; Malaysia (Malacca); Myanmar (Lin and Yang 2019).

######## Macau records.

Coloane, Hác-Sá, 28 Apr 2019, on dead tree branch, R Perissinotto & L Clennell (IZCAS); Coloane Heights, 3 May 2019, on dead tree trunk, R Perissinotto; ibidem 15 May 2020, R Perissinotto; ibidem 11 Jul 2020, R Perissinotto; Coloane Village, 2 Jun 2020, under light in ablution block, R Perissinotto & L Clennell (MACT); ibidem 5 Jun 2020, on dead tree, R Perissinotto & L Clennell (MACT); St. Francis Xavier’s Parish [Coloane], 25 Apr 2020 11:21, Kisu Wong (https://www.inaturalist.org/observations/43868250); ibidem 27 Apr 2020 00:16, Kit Chang (https://www.inaturalist.org/observations/43868602); ibidem 28 May 2020 2:33, Kit Chang (https://www.inaturalist.org/observations/47612775); ibidem 12 Jun 2020 2:36, Kit Chang (https://www.inaturalist.org/observations/49251853); ibidem 12 Jun 2020 2:44, Kit Chang (https://www.inaturalist.org/observations/49251860); ibidem 4 Apr 2021 15:08, Lynette Clennell (https://www.inaturalist.org/observations/72875242); ibidem 5 Apr 2021 11:21, Wai (https://www.inaturalist.org/observations/72974838).

######## Remarks.

In Macau, adults are active from late spring to mid-summer and range in total length 15–18 mm and 6–7.5 mm in maximum width. Individuals are readily attracted to artificial lights at night, but are also active during the day while crawling and mating on dead tree surfaces. In Hong Kong, *Mangifera indica* and *Derris* spp. have been reported as food plants for the larvae of this species (Yiu 2009). Other host plants include *Derris trifoliata*, *Hevea brasiliensis*, *Mangifera indica*, *Quercus* sp., *Shorea* sp. and *Terminalia* sp. (Lin and Yang 2019).

**Figure 45.**
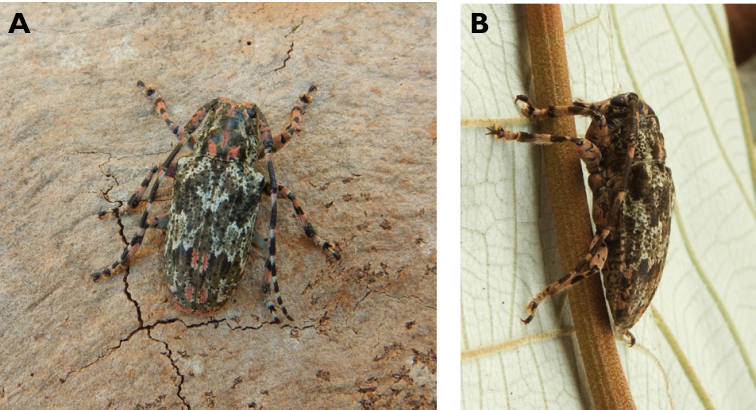
*Coptops licheneus* Pascoe, 1865: dorsal (**A**) and lateral (**B**) views of specimens observed on Coloane Heights, on 11 Jul 2020 and 26 May 2020, respectively (photographs: LC).

###### Tribe Pteropliini J. Thomson, 1860

####### Genus Desisa

Taxon classificationAnimaliaColeopteraCerambycidae

Pascoe, 1865: 163.

3CC6E81B-89F1-51CD-AFE4-48EE5804BBC4

######## Type species.

*Praonetha subfasciata* Pascoe, 1862.

####### Desisa subfasciata

Taxon classificationAnimaliaColeopteraCerambycidae

(Pascoe, 1862)

91D8FAE2-CE9F-5D1D-8EDB-AB144E180C6F

Fig. 46

Praonetha subfasciata Pascoe, 1862: 348. TL: Cambodia; TD: NHMUK

######## Distribution.

Palaearctic Region: China (Guangdong, Guangxi, Hainan, Henan, Hong Kong, Hubei, Jiangsu, Jiangxi, Yunnan, Zhejiang); India (Uttarakhand); Nepal (Yiu 2009; Lin and Yang 2019; Danilevsky 2020). Oriental Region: Cambodia; Laos; Vietnam (Hua 2002).

######## Macau records.

Coloane, A-Má Cultural Village, 28 Apr 2019, R Perissinotto & L Clennell (IZCAS); ibidem 6 May 2020, R Perissinotto; Coloane Village, 14 May 2020, at light in ablution block, R Perissinotto & L Clennell (MACT); ibidem 24 May 2020, on dead tree trunk, R Perissinotto & L Clennell; ibidem 26 May 2020, in mosquito trap, R Perissinotto & L Clennell (MACT); ibidem 31 May 2020, on mosquito trap, R Perissinotto & L Clennell (IZCAS); Great Taipa, 30 Apr 2019, on floor in ablution block, R Perissinotto & L Clennell (IZCAS); St. Francis Xavier’s Parish [Coloane], 16 May 2020 21:42, Kit Chang (https://www.inaturalist.org/observations/46100622); ibidem 3 May 2020 00:54, Kit Chang (https://www.inaturalist.org/observations/47765453); ibidem 16 May 2020 20:50, Kisu Wong (https://www.inaturalist.org/observations/53851800); ibidem 19 May 2021, Lynette Clennell (https://www.inaturalist.org/observations/79472969); ibidem 22 May 2021 8:33, Kit Chang (https://www.inaturalist.org/observations/79764029); Coloane Heights, Walking Trail, 16 May 2020 20:31, Eric Kwan (https://www.inaturalist.org/observations/46146716); Macau, 18 Jun 2020 22:13, Kelvin Joshua Che (https://www.inaturalist.org/observations/50638425).

######## Remarks.

In Macau, adults are active only in mid to late spring and range in total length 10–15 mm and 4–6 mm in maximum width. They are both nocturnal, being readily attracted to artificial lights, and diurnal, crawling and mating on dead tree branches and trunks. In Hong Kong, larvae have been found boring into various trees, including *Mallotus philippensis*, *Morus alba* and *Prunus persica* (Yiu 2009). Other reported host plants include *Bauhinia vahlii* and *Prunus armeniaca* (Lin and Yang 2019).

**Figure 46.**
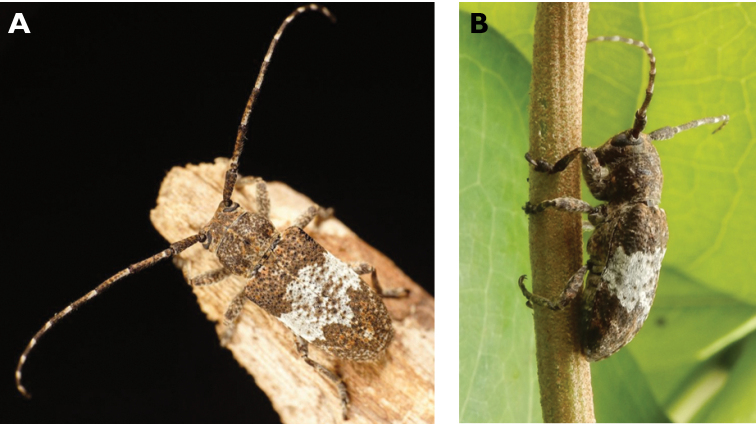
*Desisa subfasciata* (Pascoe, 1862): dorsal (**A**) and lateral (**B**) views of specimens observed on the Coloane Heights on 16 May 2020 and on 24 May 2020, respectively (photographs: **A** Kisu Wong **B** LC).

####### Genus Mispila

Taxon classificationAnimaliaColeopteraCerambycidae

Pascoe, 1864: 58.

BD83A461-ED5A-513A-BBE4-03C5062A77B1

######## Type species.

*Mispila venosa* Pascoe, 1864.

####### Mispila tholana

Taxon classificationAnimaliaColeopteraCerambycidae

(Gressitt, 1940)

A3A9F96A-8C60-533D-B4CA-F796D6522DFF

Fig. 47

Enispia tholana Gressitt, 1940a: 157, pl. 4, fig. 11. TL: China (Hainan); TD: CASF.

######## Distribution.

Palaearctic Region: China (Hainan, Yunnan) (Lin and Yang 2019; Danilevsky 2020).

######## Macau records.

Coloane Heights, A-Má Cultural Village, 28 Apr 2019, on wall near artificial light, R Perissinotto & L Clennell (IZCAS); St. Francis Xavier’s Parish [Coloane], 18 Jun 2020 22:53, Kit Chang (https://www.inaturalist.org/observations/50057362); ibidem 27 Jun 2020 2:03, Kit Chang (https://www.inaturalist.org/observations/51012816).

######## Remarks.

In Macau, adults are active only in the spring and during night time, when they are attracted to artificial lights. The only specimen that could be measured exhibited a total length of 9 mm and a maximum width of 3 mm. There is no information in the literature on the biology of this species.

**Figure 47.**
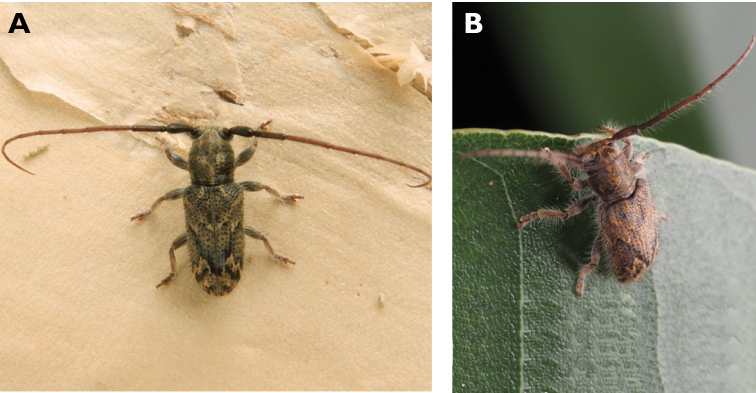
*Mispila tholana* (Gressitt, 1940): dorsal (**A**) and lateral (**B**) views of specimens observed on the Coloane Heights on 28 Apr 2019 and on 18 Jun 2020, respectively (photographs: **A** LC **B** Kit Chang).

####### Genus Prosoplus

Taxon classificationAnimaliaColeopteraCerambycidae

Blanchard, 1853: 290.

1AA7F69A-97DA-50CE-82C7-5A3955DBA09C

######## Type species.

*Leiopus sinuatofasciatus* Blanchard, 1853

####### Prosoplus bankii

Taxon classificationAnimaliaColeopteraCerambycidae

(Fabricius, 1775)

0CFFDF1F-01A7-517B-A30D-B1B8A4B77D14

Fig. 48

Lamia bankii Fabricius, 1775: 176. TL: South Africa (“Cap Bonae Spei”); TD: NHMUK.

######## Distribution.

Palaearctic Region: China (Guangdong, Hainan, Taiwan); Japan (Lin and Yang 2019; Danilevsky 2020). Oriental Region: Indonesia; Philippines; Thailand; Vietnam (Lin and Yang 2019). Also widely distributed in the Afrotropical, Australian and Pacific regions (Lin and Yang 2019).

######## Macau records.

Coloane, Tin Hau Temple, 14 Jun 2019, R Perissinotto & Lynette Clennell (IZCAS); Coloane Village, 27 May 2020, on mosquito trap, R Perissinotto & L Clennell (IZCAS); ibidem Coloane, 22 May 2020, at light in ablution block, R Perissinotto & L Clennell; Coloane Village, 2 May 2021, Lynette Clennell (https://www.inaturalist.org/observations/76790089).

######## Remarks.

In Macau, adults seem to be active only in late spring and range in total length 8–13 mm and 3–5 mm in maximum width. Individuals have so far only been found around artificial lights, indicating a predominant nocturnal activity. Hua (2002) reported as host plant for this species *Anacardium* sp., *Ananas comosus* and *Mangifera indica*.

**Figure 48.**
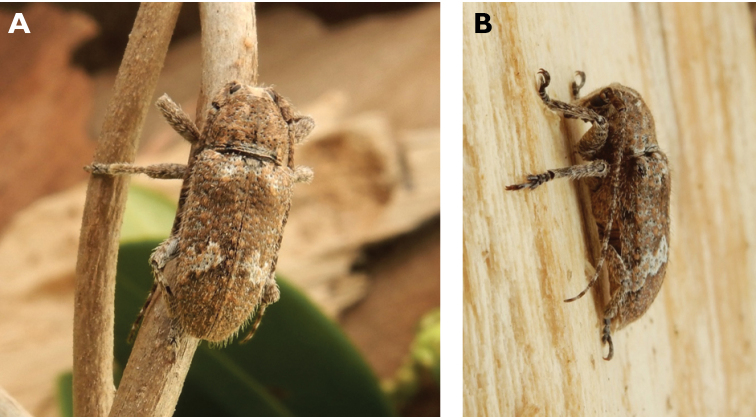
*Prosoplus bankii* (Fabricius, 1775): dorsal (**A**) and lateral (**B**) views of specimens observed at Coloane Village on 27 May 2020 and 22 May 2020, respectively (photographs: LC).

####### Genus Pterolophia

Taxon classificationAnimaliaColeopteraCerambycidae

Newman, 1842c: 370 [NP].

E11E0711-08B7-5091-915E-C318967FA276

######## Type species.

*Mesosa bigibbera* Newman, 1842.

####### Pterolophia kaleea inflexa

Taxon classificationAnimaliaColeopteraCerambycidae

Gressitt, 1940

5213A2EF-CBAA-59EC-99DC-5365A1DEDE0F

Fig. 49

Pterolophia kaleea inflexa Gressitt, 1940b: 11, pl. 1, fig. 3. TL: China (Guangdong); TD: SYSU.

######## Distribution.

Palaearctic Region: China (Fujian, Guangdong, Sichuan, Taiwan) (Lin and Yang 2019; Danilevsky 2020).

######## Macau records.

Great Taipa, 21 May 2019, at light in ablution block, R Perissinotto & L Clennell (IZCAS).

######## Remarks.

Only one female specimen was found during the census period and this exhibited a total length of 6.5 mm and a maximum width of 2 mm. The species appears to be mainly nocturnal and attracted to artificial lights. Hua (2002) reported as host plants for this species *Sophora* sp.

**Figure 49.**
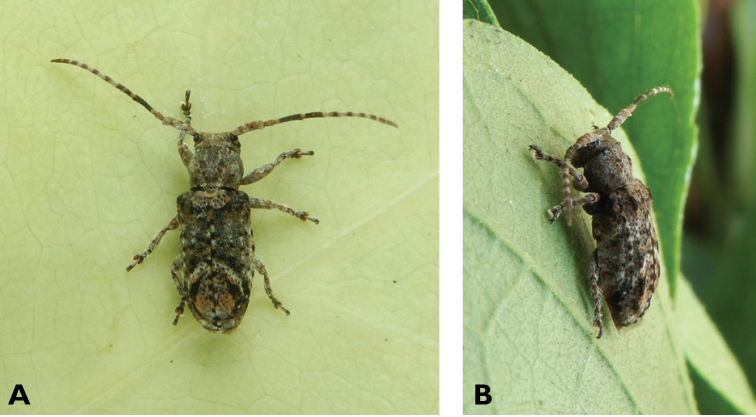
*Pterolophia kaleea inflexa* Gressitt, 1940: dorsal (**A**) and lateral (**B**) views of specimen observed on Great Taipa Hill on 21 May 2019 (photographs: LC).

####### Pterolophia consularis

Taxon classificationAnimaliaColeopteraCerambycidae

(Pascoe, 1866)

3B39E1F5-FBD2-5E8B-8507-FB1D82E0291F

Fig. 50

Praonetha consularis Pascoe, 1866: 240. TL: Malaysia (Malacca); TD: NHMUK
Pterolophia cervina Gressitt, 1939: 74. TL: China (Guangdong); TD: SYSU

######## Distribution.

Palaearctic Region: China (Guangdong, Guangxi, Guizhou, Hainan, Hong Kong, Yunnan); Bhutan; India (Sikkim) (Yiu 2009; Lin and Yang 2019; Danilevsky 2020). Oriental Region: Laos; Malaysia (Malacca); Myanmar; Indonesia (Sumatra); Vietnam (Kariyanna et al. 2017). Afrotropical Region: Madagascar (Kariyanna et al. 2017).

######## Macau records.

Coloane Village, 1 Jun 2020, on mosquito trap in ablution block, R Perissinotto & L Clennell (IZCAS); ibidem 2 May 2021, Lynette Clennell (https://www.inaturalist.org/observations/76790081).

######## Remarks.

The only two specimens observed in Macau exhibited a total length of 9–9.5 mm and a maximum width of 4 mm. Its general morphology matches rather well that of *Pterolophia cervina* Gressit, 1939 from Guangdong, which was recently considered as a synonym of *Praonetha consularis* Pascoe, 1866 (Weigel et al. 2013). It also resembles closely P. (Mimoron) brevegibbosa Pic, 1926 from Lantau Island, Hong Kong (Hayashi 1982, pl. 2, fig. 6). So, it is possible that all of them actually represent the same species, with the name P. (P.) consularis being the most senior. However, if the type species from Malaysia is different, then P. (M.) brevegibbosa will be the senior name for the species from Macau, Guangdong, Hong Kong and Hainan Island. Yiu (2009) reported that larvae of this species in Hong Kong bore into plants of *Zea mays* and Hua (2002) listed as host plant also *Casuarina equisetifolia*.

**Figure 50.**
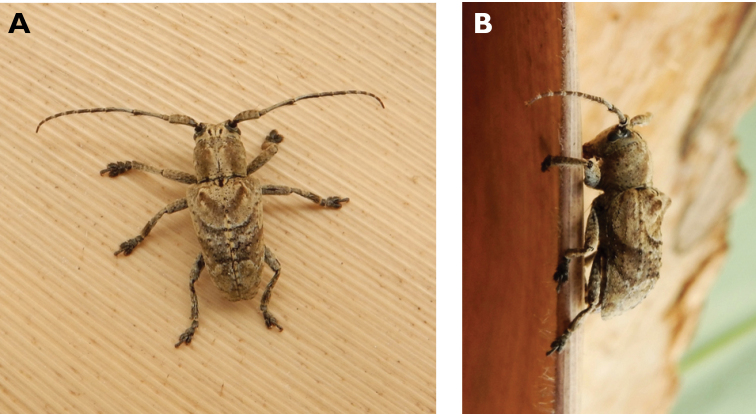
*Pterolophia consularis* (Pascoe, 1866): dorsal (**A**) and lateral (**B**) views of specimen observed at Coloane Village on 1 Jun 2020 (photographs: LC).

####### Subgenus Hylobrotus

Taxon classificationAnimaliaColeopteraCerambycidae

Lacordaire, 1872: 538.

730817FE-87A5-5B47-8CFE-4BA9D6A337A4

######## Type species.

*Hylobrotus ploemi* Lacordaire, 1872.

####### Pterolophia (Hylobrotus) annulata

Taxon classificationAnimaliaColeopteraCerambycidae

(Chevrolat, 1845)

E4CE970B-8CA9-5429-B77F-1187DB2928BF

Fig. 51

Coptops annulata Chevrolat, 1845: 99. TL: China (Macau); TD: NHMUK.
Praonetha bowringii Pascoe, 1865: 170. TL: China (Hong Kong); TD: NHMUK. Synonymised by Gressitt, 1939: 73.

######## Distribution.

Palaearctic Region: China (Fujian, Guangdong, Guangxi, Guizhou, Hainan, Hebei, Henan, Hong Kong, Hubei, Hunan, Jiangsu, Jiangxi, Shaanxi, Shanghai, Sichuan, Taiwan, Yunnan, Zhejiang); India (Sikkim); Japan; Nepal; North & South Korea (Yiu 2009; Lin and Yang 2019; Danilevsky 2020). Oriental Region: Myanmar; Thailand; Vietnam (Duffy 1968; Hua 2002).

**Figure 51.**
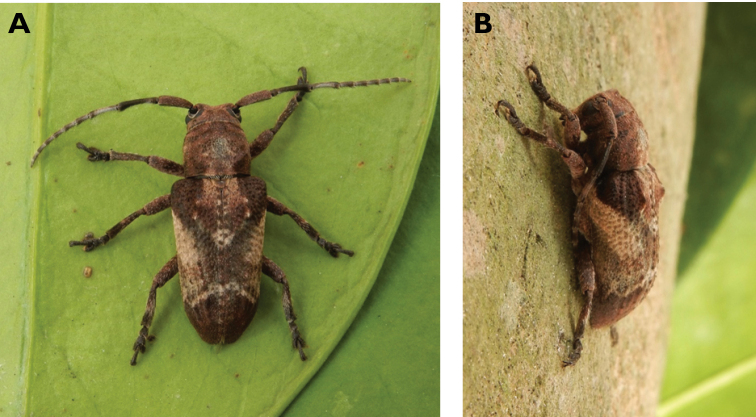
Pterolophia (Hylobrotus) annulata (Chevrolat, 1845): dorsal (**A**) and lateral (**B**) views of specimen observed at Coloane Village on 30 Apr 2020 (photographs: LC).

######## Macau records.

Coloane, 28 Jan 1992, *Morus alba*, A Fai, *Pterolophia annulata* (CIAM); No data, “*Pterolophia annulata* (Chevrolat), 桑坡天牛12 mm ” (Pun and Batalha 1997: 65, fig. 105); 1♀, Cotai Ecological Zone, 1^st^ zone, 9 Oct 2013, leg. Feng-Long Jia (SYSU); Coloane Village, 15 May 2019, in mosquito trap, R Perissinotto & L Clennell (IZCAS × 2); ibidem 31 Mar 2019, at light in ablution block, R Perissinotto; ibidem 30 Apr 2020, on dead tree branch, R Perissinotto & L Clennell (IZCAS); ibidem 13 May 2020, on mosquito trap, R Perissinotto & L Clennell (MACT); Coloane Heights, Tin Hau temple, 25 Mar 2021, crushed on pavement, R Perissinotto & L Clennell (MACT); Coloane, A-Má Statue, 20 Oct 2020 15:01, under spot-light, Lynette Clennell (https://www.inaturalist.org/observations/63084216); St. Francis Xavier’s Parish [Coloane], 24 May 2020 1:45, Kit Chang (https://www.inaturalist.org/observations/47082155); ibidem 24 May 2020 2:09, Kit Chang (https://www.inaturalist.org/observations/47082175); ibidem 28 May 2020 2:54, Kit Chang (https://www.inaturalist.org/observations/47612790); ibidem 12 Jun 2020 23:00, Kelvin Joshua Che (https://www.inaturalist.org/observations/49412284); ibidem 12 Mar 2021, Lynette Clennell (https://www.inaturalist.org/observations/71103753); ibidem 1 May 2021, Lynette Clennell (https://www.inaturalist.org/observations/76374763); Macau, Luis de Camoes Garden, 10 Jun 2020 10:30, Eric Kwan (https://www.inaturalist.org/observations/49141547).

######## Remarks.

In Macau, adults are active from early spring till mid-autumn and range in total length 11–15 mm and 4–6 mm in maximum width. The species is mainly nocturnal and promptly attracted to artificial lights. In Hong Kong, larvae of this species bore into wood of *Morus alba* (Yiu 2009). Elsewhere, larval host plants include also *Albizia julibrissin*, *Celtis sinensis*, *Ficus pumila*, *Machilus thunbergii*, *Pinus massoniana* and *Prunus persica* (Hua 2002; Lim et al. 2014).

###### Tribe Saperdini Mulsant, 1839

####### Genus Glenea

Taxon classificationAnimaliaColeopteraCerambycidae

Newman, 1842d: 301.

39314980-016B-5247-AB3F-6421044F4645

######## Type species.

*Saperda novemguttata* Guérin-Méneville, 1831, designated by Thomson 1879: 1.

####### Subgenus Stiroglenea

Taxon classificationAnimaliaColeopteraCerambycidae

Aurivillius, 1920

9B252251-D323-581C-8A47-0CE5A9A77C56

Glenea Sg. Stiroglenea Aurivillius, 1920: 30.

######## Type species.

*Lamia cantor* Fabricius, 1787.

####### Glenea (Stiroglenea) cantorcantor

Taxon classificationAnimaliaColeopteraCerambycidae

(Fabricius, 1787)

13C13DB1-F292-5F9B-8EAA-2C94B962599D

Fig. 52

Lamia cantor Fabricius, 1787: 142. TL: China; TD: ZMUC

######## Distribution.

Palaearctic Region: China (Guangdong, Guangxi, Guizhou, Hainan, Hong Kong, Jiangxi, Yunnan, Zhejiang) (Yiu 2009; Lin and Yang 2019; Danilevsky 2020). Oriental Region: India; Laos; Philippines; Thailand; Vietnam (Kariyanna et al. 2017).

**Figure 52.**
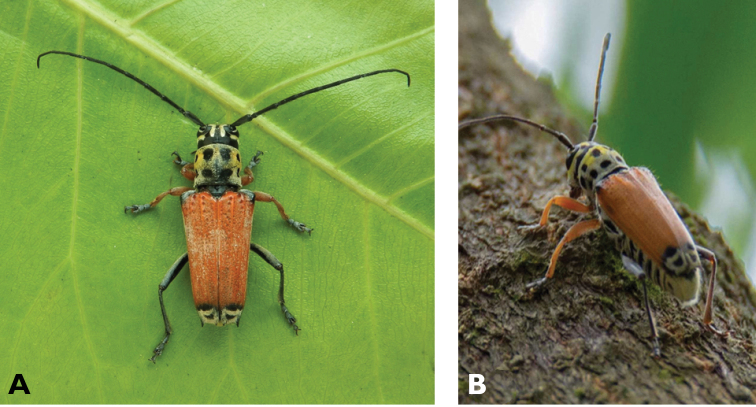
Glenea (Stiroglenea) cantor cantor (Fabricius, 1787): dorsal (**A**) and lateral (**B**) views of specimens observed on Coloane Heights on 23 Sep 2020 and 15 Sep 2019, respectively (photographs: **A** LC **B** Eric Kwan).

######## Macau records.

1♀, Coloane, 20 Jul 1994, Tai Ip, *Glenea cantor* (CIAM); No data, “*Glenea cantor* (Fabricius), 眉斑并脊天牛15 mm” (Pun and Batalha 1997: 64, fig. 99); Coloane, 15 Sep 2019, Eric Kwan; Coloane, Seac Pai Van Road, 23 Sep 2020 11:23, Lynette Clennell (https://www.inaturalist.org/observations/60909845) (IZCAS); ibidem, inside Seac Pai Van Park, 28 May 2021 8:10, Macau Friend (https://www.inaturalist.org/observations/80630954).

######## Remarks.

This species has been recorded only three times in Macau during the current census, in late spring and late summer. Adult activity is mainly during the hottest part of the day, when individuals promptly take off in flight when disturbed in the forest undergrowth, or display thanatosis if captured (Yiu 2009; pers. obs.). The only specimen that could be measured had a total length of 13 mm and a maximum width of 4 mm. The host plants known so far in its wide distribution range include *Aesculus chinensis*, *Bombax ceiba*, *B. malabaricum*, *Castanea mollissima*, *Ceiba pentandra*, *Excentrodendron hsiemmu*, *Melastoma candidum*, *Melia azedarach*, *Paulownia* sp. and *Quercus* sp. (Hua 2002; Yiu 2009; Lin and Yang 2019).

####### Genus Oberea

Taxon classificationAnimaliaColeopteraCerambycidae

Dejean, 1835: 351.

7F749D0B-2758-526A-9EA7-23ACE3FB98B2

######## Type species.

*Cerambyx linearis* Linnaeus, 1760.

####### Oberea ferruginea

Taxon classificationAnimaliaColeopteraCerambycidae

(Thunberg, 1787)

46055516-5965-5C86-AFB8-3FACC53FB5E3

Fig. 53

Saperda ferruginea Thunberg, 1787: 57. TL: Unknown; TD: UZIU.
Oberea semiargentata Pic, 1923: 15. TL: China (Guangdong); TD: MNHN.
Oberea notativentris Pic, 1924: 30. TL: China (Guangdong); TD: MNHN. [RN] Synonymised by Breuning 1962b: 159.

######## Distribution.

Palaearctic Region: China (Fujian, Gansu, Guangdong, Guangxi, Hubei, Hunan, Shaanxi, Yunnan); India (Sikkim); Nepal (Lin and Yang 2019; Danilevsky 2020). Oriental Region: Laos; Malaysia; Myanmar; Vietnam (Hua 2002).

**Figure 53.**
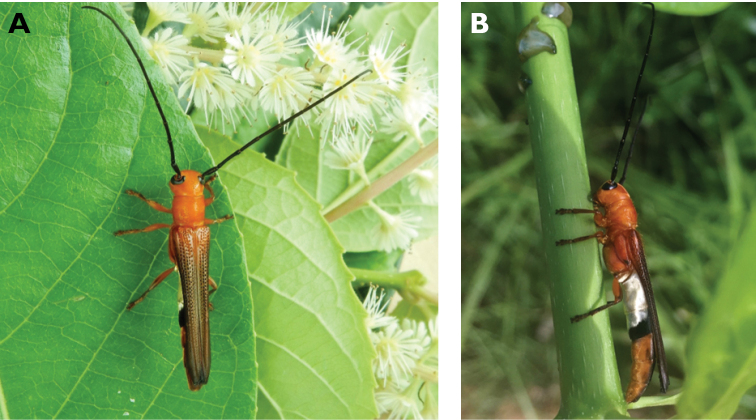
*Oberea ferruginea* (Thunberg, 1787): dorsal (**A**) and lateral (**B**) views of specimens observed on Little Taipa Hill (11 Oct 2018) and around Hác-Sá Dam (4 Jul 2020), respectively (photographs: **A** LC **B** Annie Lao).

######## Macau records.

1♂, Taipa, 14 Jul 1992, Dr Easton, *Oberea ferruginea* (CIAM); Trilho da Taipa Grande, 19 Sep 1995, hovering over false tea, ER Easton, J Bizarro & T Novo leg (UMEC); No data, “*Oberea ferruginea* Thunberg, 短足筒天牛16 mm” (Pun and Batalha 1997: 65, fig. 102); 1♀, Coloane, 18 Jul 1996, KW Ho, *Oberea ferruginea* (CIAM); 1♂, ibidem 20 Jul 1994, WM Ng, *Oberea ferruginea* (CIAM); 1♀, ibidem 25 Apr 2000, ML Lei (CIAM); 1♂, ibidem 8 Aug 2002, KL Tang (CIAM); Little Taipa Hill, 26 Sep 2018, R Perissinotto & L Clennell; ibidem 11 Oct 2018, R Perissinotto & L Clennell; Taipa, Lou Lim Ieok Road, 4 Apr 2019 R Perissinotto & L Clennell (IZCAS); ibidem 23 Apr 2019, on shrub leaves, R Perissinotto & L Clennell (IZCAS); ibidem 26 Oct 2018, R Perissinotto & L Clennell (MACT); Great Taipa, 20 Apr 2020, R Perissinotto; Great Taipa, 13 Oct 2020, dead on shrub leaf, R Perissinotto & L Clennell (IZCAS); Coloane Heights, 15 May 2020, dead on trail path, R Perissinotto (MACT); St. Francis Xavier’s Parish [Coloane], 24 Oct 2019 12:15, Stanley Chan (https://www.inaturalist.org/observations/34898344); ibidem 23 Mar 2021 10:21, Kit Chang (https://www.inaturalist.org/observations/71862552); Coloane, Hác-Sá Dam, 4 Jul 2020 15:43, Annie Lao (https://www.inaturalist.org/observations/51888124); Macau, Lou Lim Loc Garden, 25 Aug 2020 14:19, Eric Kwan (https://www.inaturalist.org/observations/57519347); Coloane, 13 May 2021, Lynette Clennell (https://www.inaturalist.org/observations/78657008).

######## Remarks.

In Macau, adults are active during daytime from early spring till mid-autumn and range in total length 16–23 mm and 2.5–4 mm in maximum width. The larvae of this species are known stem-borers of a variety of plants, including *Bambusa* spp., *Schima superba* and *Vernicia fordii* (Hua 2002).

####### Oberea walkeri

Taxon classificationAnimaliaColeopteraCerambycidae

Gahan, 1894

E8EA1F31-DE48-5433-963D-8D0977F9B2E3

Fig. 54

Oberea walkeri Gahan, 1894: 487. TL: China (Hong Kong); TD: NHMUK

######## Distribution.

Palaearctic Region: China (Fujian, Guangdong, Guangxi, Guizhou, Hainan, Henan, Hong Kong, Hunan, Jiangxi, Shaanxi, Sichuan, Xizang, Yunnan, Zhejiang); India (Sikkim) (Yiu 2009; Lin and Yang 2019; Danilevsky 2020). Oriental Region: Laos; Myanmar; Vietnam (Kurihara 2009).

######## Macau records.

Coloane Heights, A-Má Cultural Village, 28 Apr 2019, R Perissinotto & L Clennell (IZCAS); Great Taipa, Barbeque Park, 9 May 2019, R Perissinotto & L Clennell (IZCAS); Coloane, 20 Apr 2021, Lynette Clennell (https://www.inaturalist.org/observations/74708252) .

######## Remarks.

This species is rather scarce in Macau, having been observed only three times and only in mid spring. Adults are active during daytime and range in total length 14–18 mm and 2.5–4 mm in maximum width. The only larval host plant reported so far for this species is *Sassafras tzumu* (Hua 2002).

**Figure 54.**
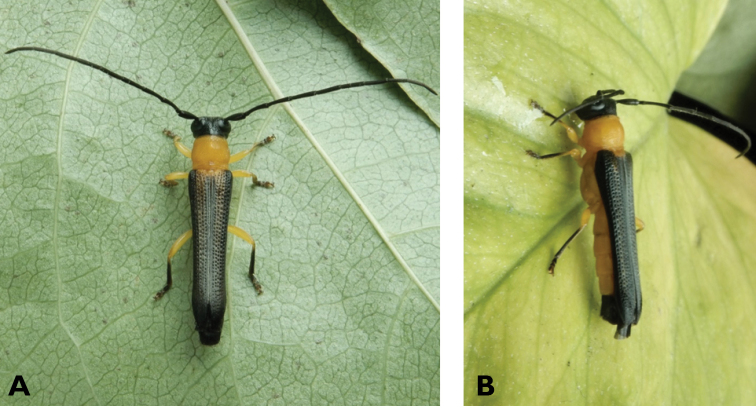
*Oberea walkeri* Gahan, 1894: dorsal (**A**) and lateral (**B**) views of specimens observed on Great Taipa Hill (9 May 2019) and on Coloane Heights (28 Apr 2019), respectively (photographs: LC).

## Discussion

A total of 52 longhorn beetle species was recorded during this census, 2.6 times more than previously reported in the literature for this area (Easton 1991, 1992, 1993; Pun and Batalha 1997; Lin and Yang 2019). Among these, two are new records for China and one also for the entire Palaearctic Region. These findings are undoubtedly due mainly to the exceptional observation efforts undertaken in this study during the past 3.5 years, with diurnal and nocturnal visits to key areas conducted on a daily basis. By comparison, in nearby Hong Kong a total of 137 species has been recorded thus far (Yiu 2009; Yiu and Yip 2011; Lin and Aston 2014). This is expected, as the total surface area of the Hong Kong SAR is ca. 36 × larger than that of Macau and exhibits a much larger diversity of vegetation types and habitats (Dudgeon and Corlett 1994). It is noteworthy that despite their close geographic proximity, these two regions of the Pearl River Delta actually exhibit a distinct difference in their longhorn beetle composition, as already pointed out for instance in the historical reports by Easton (1991, 1992, 1993). It is also likely that some species do not have viable resident populations in Macau and that their occasional presence there may be due to stray specimens landing randomly during their dispersal flights from either the Hong Kong islands or the Chinese mainland (i.e., Guangdong Province).

There are, however, still species that while previously recorded from Macau were not encountered during the current census. These include *Imantocera penicillata* (Hope, 1831), *Aristobia approximator* (Thomson, 1865), *Apriona germarii* (Hope, 1831), *Batocera horsfieldi* (Hope, 1839), *Pothyne rugifrons* Gressitt, 1940 (Easton 1991, 1992, 1993; Pun and Batalha 1997) and Pterolophia (Pterolophia) crassipes (Wiedemann) (Hua 2002; Lin and Yang 2019). *Imantocera penicillata* was reported in the accounts by both Easton (1991, 1993) and Pun and Batalha (1997), with the former author observing this species attracted by artificial lights to the Taipa university buildings and to breadfruit or jackfruit trees (*Artocarpus* spp.) on the island of Coloane (Easton 1993). Indeed, several old specimens of this species are still housed in the UMEC and CIAM collections, as testimony of its historical presence in the region. *Pothyne rugifrons* and *B. horsfieldi* were only reported in Pun and Batalha (1997), and a few specimens of both species collected in the 1980s–1990s at Coloane are currently housed in the CIAM collection. The first species is known to occur also in nearby Hong Kong, while *B. horsfieldi* has not been recorded there yet (Yiu 2009; Yiu and Yip 2011) but is known to occur throughout mainland China (Lin and Yang 2019). On the other hand, *A. germarii* and *A. approximator*, reported in Pun and Batalha (1997) and Easton (1992), respectively, both appear to represent erroneous identifications. They do not occur in south-eastern China and therefore the correct species involved are actually *A. rugicollis* Chevrolat, 1852 and *A. reticulator* (Fabricius 1781), respectively, which are well known from nearby Hong Kong albeit reported in the past literature as either *A. germarii* the first, or with its invalid synonym of “*A. testudo* (Voet, 1778)” the second (Yiu 2009; Yiu and Yip 2011). Similarly, Pterolophia (P.) crassipes (Wiedemann, 1823) was first mentioned from Macau by Gemminger and Harold (1873), and subsequently also included in the catalogues of Hua (2002) and Lin and Yang (2019). However, it was not found in any of the more recent studies, including this survey, and therefore it is presumed that its initial Macau identification may have been erroneous.

On the more concerning side, this census has also revealed that while longhorn species diversity in Macau is remarkably higher than previously reported, the relative abundance and frequency of occurrence of most species is actually extremely low. This is an unfortunate development that is currently being reported from across the whole world, as the so-called “insect apocalypse” (Jarvis 2018; Cardoso et al. 2020). This drastic global reduction in insect abundance and biomass has been attributed to several compounding causes, chiefly habitat loss and fragmentation, intensive use of pesticides, light, air, and noise pollution as well as climate change (Samways et al. 2020).

In Macau, all these factors are exacerbated by high human population density and sophisticated infrastructure. Most of the beetle species observed during this study are predominantly nocturnal in their adult activity, and, therefore readily attracted to artificial lights. Thus, like in all nocturnal insects their orientation and navigation are disrupted when light pollution interferes with the natural light from the moon or stars they generally use for these purposes (Cardoso et al. 2020). Changes in natural light/dark cycles also de-synchronise vital activities, such as feeding and egg-laying, and cause temporal mismatches in mutualistic interactions (Owens and Lewis 2018). To add to this, UV-light mosquito traps, like those deployed in all public ablution blocks in Macau, attract and electrocute a wide variety of non-target insects, including all the nocturnal longhorn beetles that can pass through the protective grid of these traps (RP & LC pers. obs.). An inordinate proportion of specimens observed during this census were also found crushed by vehicles or pedestrians on roads and paths under artificial illumination, killed by thermal shock on the surface of incandescent spot-lights or otherwise dismembered by insectivore birds at illuminated sites in the early morning hours.

As virtually all the longhorn beetles found in this region are xylophagous or saproxylic, with larval development depending entirely on availability of dead or dying trees that are preferably still standing (Nieto and Alexander 2010), adequate management of the remaining forest patches is of critical importance in Macau. Unfortunately, these habitats which are already extremely reduced and fragmented are under increasing pressure from recreational infrastructure development and aesthetic sanitation. Dead, damaged, and diseased trees are systematically removed and shredded for compost, or alternatively cut to small pieces and left on the ground. While the latter option may offer some habitat space for a limited number of xylophagous and saproxylic species, the vast majority of them will be prevented from colonising this wood, as ground-based predators such as ants, spiders and centipedes will rapidly take over. These trees, which are often veteran, are normally replaced with young trees, but these are planted in a plantation-type manner, with ample space between each other and all understorey continuously removed in between. Trees are also regularly pruned of their lower branches, in order to accelerate their growth in height. To quote from one of the latest “Scientists’ Warning to Humanity” publications: “forest recovery entails more than just the trees, but also the epiphytes, a natural understorey, dead wood, and leaf litter... restoration should aim at a natural age structure, including veteran trees... as forests have high structural diversity, possess many microhabitats, and create sheltered microclimates allowing many species to co-exist under optimal conditions” (Samways et al. 2020).

## Supplementary Material

XML Treatment for Philus

XML Treatment for Philus antennatus

XML Treatment for Philus pallescens pallescens

XML Treatment for Aegolipton

XML Treatment for Aegolipton marginale

XML Treatment for Cephalallus

XML Treatment for Cephalallus unicolor unicolor

XML Treatment for Chelidonium

XML Treatment for Chelidonium argentatum

XML Treatment for Embrikstrandia

XML Treatment for Embrikstrandia unifasciata

XML Treatment for Polyzonus

XML Treatment for Polyzonus sinensis

XML Treatment for Ceresium

XML Treatment for Ceresium elongatum elongatum

XML Treatment for Ceresium longicorne

XML Treatment for Ceresium sinicum ornaticolle

XML Treatment for Ceresium zeylanicum

XML Treatment for Trirachys

XML Treatment for Trirachys indutus

XML Treatment for Rhytidodera

XML Treatment for Rhytidodera integra

XML Treatment for Chlorophorus

XML Treatment for Chlorophorus annularis

XML Treatment for Chlorophorus macaumensis macaumensis

XML Treatment for Demonax

XML Treatment for Demonax bimaculicollis

XML Treatment for Perissus

XML Treatment for Perissus indistinctus

XML Treatment for Stromatium

XML Treatment for Stromatium longicorne

XML Treatment for Kuegleria

XML Treatment for Kuegleria annulicornis

XML Treatment for Nysina

XML Treatment for Nysina rufescens asiatica

XML Treatment for Pyrestes

XML Treatment for Pyrestes haematicus

XML Treatment for Purpuricenus

XML Treatment for Purpuricenus temminckii sinensis

XML Treatment for Xystrocera

XML Treatment for Xystrocera globosa

XML Treatment for Rondibilis

XML Treatment for Rondibilis undulata

XML Treatment for Apomecyna

XML Treatment for Apomecyna longicollis longicollis

XML Treatment for Apomecyna saltator

XML Treatment for Ropica

XML Treatment for Ropica dorsalis

XML Treatment for Sybra

XML Treatment for Sybra marmorea

XML Treatment for Sybra posticalis

XML Treatment for Batocera

XML Treatment for Batocera rubus rubus

XML Treatment for Pseudoterinaea

XML Treatment for Pseudoterinaea bicoloripes

XML Treatment for Sophronica

XML Treatment for Sophronica apicalis

XML Treatment for Zotalemimon

XML Treatment for Zotalemimon ciliatum

XML Treatment for Olenecamptus

XML Treatment for Olenecamptus taiwanus

XML Treatment for Exocentrus

XML Treatment for Exocentrus alboguttatus subconjunctus

XML Treatment for Exocentrus formosofasciolatus

XML Treatment for Bumetopia

XML Treatment for Bumetopia oscitans

XML Treatment for Anoplophora

XML Treatment for Anoplophora chinensis chinensis

XML Treatment for Blepephaeus

XML Treatment for Blepephaeus subcruciatus

XML Treatment for Blepephaeus succinctor

XML Treatment for Eutaenia

XML Treatment for Eutaenia tanoni

XML Treatment for Monochamus

XML Treatment for Monochamus alternatus alternatus

XML Treatment for Coptops

XML Treatment for Coptops licheneus

XML Treatment for Desisa

XML Treatment for Desisa subfasciata

XML Treatment for Mispila

XML Treatment for Mispila tholana

XML Treatment for Prosoplus

XML Treatment for Prosoplus bankii

XML Treatment for Pterolophia

XML Treatment for Pterolophia kaleea inflexa

XML Treatment for Pterolophia consularis

XML Treatment for Subgenus Hylobrotus

XML Treatment for Pterolophia (Hylobrotus) annulata

XML Treatment for Glenea

XML Treatment for Subgenus Stiroglenea

XML Treatment for Glenea (Stiroglenea) cantorcantor

XML Treatment for Oberea

XML Treatment for Oberea ferruginea

XML Treatment for Oberea walkeri
